# Molecular Characterization of Vitellogenin and Vitellogenin Receptor of *Bemisia tabaci*

**DOI:** 10.1371/journal.pone.0155306

**Published:** 2016-05-09

**Authors:** Santosh Kumar Upadhyay, Harpal Singh, Sameer Dixit, Venugopal Mendu, Praveen C. Verma

**Affiliations:** 1 Department of Botany, Panjab University, Chandigarh, 160014, India; 2 CSIR-National Botanical Research Institute, Council of Scientific and Industrial Research, Rana Pratap Marg, Lucknow, 226001, India; 3 Fiber & Biopolymer Research Institute (FBRI), Department of Plant and Soil Science, Texas Tech University, Food Technology Building, Lubbock, TX, 79409–2122, United States of America; Volcani Center, ISRAEL

## Abstract

Vitellogenin (Vg) plays vital role in oocytes and embryo development in insects. Vg is synthesized in the fat body, moves through haemolymph and accumulates in oocytes. Vitellogenin receptors (VgR) present on the surface of oocytes, are responsible for Vg transportation from haemolymph to oocytes. Here, we cloned and characterized these genes from *Bemisia tabaci* Asia1 (BtA1) species. The cloned *BtA1Vg* and *BtA1VgR* genes consisted of 6,330 and 5,430 bp long open reading frames, which encoded 2,109 and 1,809 amino acid (AA) residues long protein. The BtA1Vg protein comprised LPD_N, DUF1943 and VWFD domains, typical R/KXXR/K, DGXR and GL/ICG motifs, and polyserine tracts. BtA1VgR protein contained 12 LDLa, 10 LDLb and 7 EGF domains, and a trans-membrane and cytoplasmic region at C-terminus. Phylogenetic analyses indicated evolutionary association of BtA1Vg and BtA1VgR with the homologous proteins from various insect species. Silencing of *BtA1VgR* by siRNA did not affect the transcript level of *BtA1Vg*. However, BtA1Vg protein accumulation in oocytes was directly influenced with the expression level of *BtA1VgR*. Further, *BtA1VgR* silencing caused significant mortality and reduced fecundity in adult whiteflies. The results established the role of BtA1VgR in transportation of BtA1Vg in oocytes. Further, these proteins are essential for fecundity, and therefore these can be potential RNAi targets for insect control in crop plants.

## Introduction

Whitefly (*Bemisia tabaci)* is globally considered as a polyphagous agricultural pest and consists of more than 24 morphologically indistinguishable species [1]. They had caused severe direct and indirect damage to many crops by feeding and transmitting viruses to plants, resulting in major loss in crop yield and millions of dollars annually [2]. Whiteflies are significantly loosing susceptibility to different classes of insecticides and showing resistance to pymetrozine and endosulfan [3,4]. Hence, understanding the biology of whitefly is essential to develop precise alternative methods for their control in crop plants.

Vitellin (Vn) is a storage protein in eggs of oviparous animals including insects, which is produced from precursor protein [vitellogenin-(Vg)]. Vg is produced in fat body of insect by extensive structural alterations such as phosphorylation, lipidation, proteolytic cleavage, and glycosylation, etc. of the protein before their secretion and transfer into the ovaries [5]. Vitellin provides nutrition in oogenesis for egg and embryonic development. Vitellogenin receptors (VgR) play a decisive role in vitellogenesis and uptake of vitellogenin by oocytes during its development [6]. During vitellogenesis, production of yolk resources is essential for egg maturation and helps in embryo development after egg laying. Till now, the Vgs and VgRs have been identified from many insect species [5–16]. All the identified VgRs belong to low-density lipoprotein receptor (LDLR) super family and comprise common structural elements like cysteine-rich ligand-binding repeats (LBRs), cysteine-rich epidermal growth factor precursor (EGFP) like repeats linked by a single transmembrane domain, a short carboxyl-terminal cytoplasmic tail and cysteine-poor spacer regions [17]. Despite of common structural elements there are differences in their physiological role [18,19].

The expression of vitellogenin in *B*. *tabaci* is dynamic and varies during different developmental stages, but the roles of VgR and Vg during the reproduction and growth is still not clear. Further, *B*. *tabaci* is a complex insect species which differ in their physiology, genetic composition, mating behavior, fecundity and several other characteristics [1,20]. Therefore the characterization of genes needs to be established in each species to better understand the molecular mechanism. Understanding the interaction of vitellogenin and its receptors is essential for revealing the mechanism of reproduction in insects. It will also be useful in developing new strategies of insect pest control. Here, we report the molecular characterization of Vg and VgR of *B*. *tabaci* Asia1 species, which is one of the most prevalent species found in India [21]. The BtA1Vg and BtA1VgR encoding genes were cloned and used for *in-silico* and molecular characterization, and compared with the other insects. The expression analysis at different developmental stages and evolutionary relation between the insects Vgs and VgRs were also studied. Further, we analyzed the role of BtA1VgR in transportation of BtA1Vg from haemolymph to oocytes, and effect of their reduced expression on the survival of *B*. *tabaci* by utilizing the RNA interference tool.

## Materials and Methods

### Insect rearing and collection

*Bemisia tabaci* Asia1 species culture was maintained on cotton plants in laboratory as described earlier [22]. Sequences of cytochrome oxidase I and ITS1 genes were used to determine the purity of culture [23]. Different developmental stages of whitefly were collected, frozen using liquid nitrogen and stocked at -80°C for later use. About 500 eggs, 300 nymphs and 200 adults were collected and used at different stages of the study. Since the laboratory culture was used in this study, which did not required any specific permission.

### RNA isolation and cDNA synthesis

Total RNA was isolated from each sample separately using Tri reagent as per the standard protocol (Sigma, USA). DNA-free Kit (Ambion, USA) was used to remove the DNA contamination from the RNA preparations. The RNA samples integrity was analysed on 2100 Bioanalyzer (Agilent Technologies, USA). To clone the genes, cDNA was synthesized using SMARTer^™^ RACE cDNA Amplification Kit (Clontech, USA) following the manufacturer’s protocol. However for the expression analysis, cDNA was synthesized using the First Strand cDNA Synthesis Kit (Invitrogen, USA).

### Database mining for sequences encoding vitellogenin and vitellogenin receptor

The protein sequences of vitellogenin and vitellogenin receptor of *Drosophila melanogaster*, *Nilaparvata lugens* and *Blattella germanica*, were downloaded from NCBI database (S1 and S2 Tables). The above sequences were used for tbastn search against transcriptome shotgun assembly (TSA) sequences of *B*. *tabaci* at NCBI (http://blast.ncbi.nlm.nih.gov/Blast.cgi). Further, these sequences were also searched against the local transcriptome database generated from *B*. *tabaci* Asia1 during earlier study [23]. The matched sequences were retrieved and their identity was further confirmed by blastx search against non-redundant protein database at NCBI.

### Cloning of complete *vitellogenin* and *vitellogenin receptor* genes

The identified TSA sequences were used to design gene specific primers from the conserved region to get the entire gene sequence by RACE (Rapid Amplification of cDNA Ends). The TSA sequences used for primer designing were GAPQ01001336.1 and GAPP01001385.1 for *vitellogenin*, and EZ959208.1 and EZ943568.1 for *vitellogenin receptor*. Since these genes are very large in size, primers were designed using the two TSA sequences for RACE as well as, in between those TSA sequences to ease the amplification and sequencing of the gene (Table 1). RACE (5’ and 3’) was performed using the RACE Kit following the standard protocol (Clontech, USA). Amplified DNA fragment was cloned and sequenced. Complete gene sequence was obtained by assembling the both 5’ and 3’ RACE sequences.

**Table 1:** Primers used in the cloning and expression analysis of *vitellogenin* (Vg) and *vitellogenin receptor* (VgR) genes.

**Primers used for RACE**		
**Gene**	**5’ RACE (5’-3’)**	**3’ RACE (5’-3’)**
**Vitellogenin**		
GSP1	TCTTGGGGTTAAGAAGATCGACG	GAGACAACAGTCACGGCCAAAAG
NGSP1	CGGATTTCTTGGCGGCGTTGC	CGAATTCAACTTCGAACCCGCC
GSP2	TTCATAGTCCTCTTCGCTGCTGC	CCAACGGTGACAGCCGCAAGAT
NGSP2	GCTGGATGAGGAGCTGGAAGAG	GCTCACCCCAAGATCCTCGAAG
**Vitellogenin receptor**		
GSP1	GATCACGGCCTTGAAAATCAACAG	GCGCTGTCCTCCACAACAAAGAC
NGSP1	GATTTCCGTGATCAATTGTGAGACC	CCCAGCTGATGCGGTCATGTAC
GSP2	CATCATTGCATACAAGATCATAAGTG	GCTCATATTCCCAATGTGTGCCAT
NGSP2	GCCAGGCTCGCACATGAACTCC	GATGATTTCAGTCTAGTCTGCAAC
**Primers used for amplification between selected TSA sequences**		
	**Forward primer (5’-3’)**	**Reverse Primer (5’-3’)**
**Vitellogenin**	CTCTTCCAGCTCCTCATCCAGC	GGCGGGTTCGAAGTTGAATTCG
**Vitellogenin receptor**	GACTTCTATGAGTTTGTAACGACA	AGTGATGCATTTCCCATTATTGC
**Primers used for transcript expression analysis**		
***Vitellogenin***	GCTCGTTTCCCAGACAAGG	CTCAGCGAAAGCAAGGAGAG
***Vitellogenin receptor***	GCAGAAGAGGGTGAAGGAC	CCAATAGACATGTTGACCATC

### Characterization of vitellogenin and vitellogenin receptor

Open reading frame of each gene was obtained by ORF finder (http://www.ncbi.nlm.nih.gov/gorf/orFig.cgi), which was reconfirmed by blast search against NCBI database. Expasy translate tool (http://web.expasy.org/translate/) was used to obtain the encoding protein sequence for each gene. The gene sequences for *BtA1Vg* and *BtA1VgR* were submitted to NCBI sequence database. Expasy MW/pI tool (http://web.expasy.org/compute_pi/) was used for determining the theoretical molecular mass and pI of proteins. The sequence similarities with other available insect’s vitellogenin and vitellogenin receptor sequences were analyzed by blastP search at NCBI databases (http://blast.ncbi.nlm.nih.gov/Blast.cgi). Domain architecture and conserved domains were identified using Scan-Prosite (http://prosite.expasy.org/scanprosite/), SMART (http://smart.embl-heidelberg.de/) and InterProScan (http://www.ebi.ac.uk/Tools/pfa/iprscan/) online tools. Presence of signal peptide was predicted by using SignalP 4.1 Server (http://www.cbs.dtu.dk/services/SignalP/). The transmembrane regions were predicted by TMHMM server v2.0 (http://www.cbs.dtu.dk/services/TMHMM/). TMHMM server predicts transmembrane protein topology with hidden Markov model. Cellular localization was predicted by PSORT II (http://psort.hgc.jp/form2.html). The ConSurf server (http://consurf.tau.ac.il/overview.html) was used for estimating the evolutionary conservation of amino acid positions in proteins. Multiple sequence alignments were performed using clustalW (http://www.ebi.ac.uk/Tools/msa/clustalw2/) and muscle with the known sequences of selected insects representing each order and other *B*. *tabaci* species. For phylogenetic analysis, sequences were aligned using muscle [24], aligned fragments were extracted and phylogenetic tree was generated by a Neighbor Joining method using MEGA6 with bootstrap of 1000 replicates [25]. Maximum likelihood analysis was also performed to reanalyze the results.

### Expression analysis

Total RNA was isolated from egg, nymph and adult insects and used for cDNA synthesis as described above. Equal quantity of cDNA from different developmental stages was used to perform relative expression analysis of *BtA1Vg* and *BtA1VgR* mRNA by using real time PCR machine (GeneAmp 5700 real time PCR machine, Applied Biosystems, USA) using SYBR Green (Invitrogen, USA). Primers used for expression analysis are listed in Table 1. The expression analysis was performed in three biological replicates and expression of *actin* gene was used as internal control as described earlier [26,27].

To compare the level of BtA1Vg protein accumulation, ovary and haemolymph samples were collected following the methods described [28]. Total soluble protein was collected and analyzed onto the 10% SDS-PAGE following the standard protocol. The BtA1Vg protein could be easily detected at ~240 kDa due to relatively large quantity. The identity was confirmed by mass spectrometry analysis of trypsinized protein on MALDI-TOF-TOF platform (model 4800, ABsciex, USA) following the earlier established protocol [29]. Since vitellogenins are lipophosphoglycoproteins, the protein band was further confirmed by staining with Sudan Black B solution (Sigma), Methyl Green (GelCode Phosphoprotein Staining Kit, Thermo Scientific) and Periodic acid-Schiff’s reagent (Glycoprotein detection kit, Sigma), separately (S1 Fig). The quantity of protein between different stages was compared using the standard densitometry of the vitellogenin band after staining with different solutions in triplicates as described above.

### *In-vitro* synthesis of siRNA and feeding

A fragment (143 bp) of *BtA1VgR* gene was amplified using the same primers used for expression analysis. The amplified fragment was cloned in between the T7 promoters as described [22]. Double stranded RNA (dsRNA) was synthesized using MEGAscript^®^RNAi Kit (Ambion, USA) and digested into the siRNA by RNAseIII enzyme. To study the effect of *BtA1VgR* siRNA on the expression of BtA1VgR and BtA1Vg, the siRNA was purified and fed in different concentration (5, 10, 20 and 40 μg/ml) to the adult insects following the earlier established protocol [22]. An unrelated siRNA synthesized from a plant gene *Allium sativum* agglutinin was used as control. The survival of insect was calculated after every 24 h for three days. Afterward, equal number of control and *BtA1VgR* siRNA (40 μg/ml) fed surviving insects were released on potted cotton plants separately. Pots were covered with nylon mesh and placed at 26±2°C and 80% relative humidity. The leaves were detached from the plants after 20 days and egg laying patterns were analyzed under light microscope (Leica MZ-125). The statistical analyses were performed using SPSS program (version 10). One-way ANOVA was used to analyse the data and means were compared using Duncan’s multiple range test (DMRT).

## Results and Discussion

### Isolation of v*itellogenin* and *vitellogenin receptor* genes

Vg plays important role in the life cycle of insects by providing nutrition during oocytes and embryonic development [30]. Vg biosynthesis is usually regulated by juvenile hormone in insects [31]. It is synthesized by fat body and secreted into the hemolymph, where it interacts with VgR and sequestered by competent oocytes [32]. Molecular characterization of Vg and VgR had been performed in several insects [33]. In the last decade, some genomic information has been available for different species of *B*. *tabaci* [23,34]. A few studies are also performed to characterize Vg and VgR in *B*. *tabaci* [14,28], however the detailed molecular characterization is still required in various species. We have also observed significant expression of *Vg* and *VgR* transcripts in *B*. *tabaci* Asia1 during transcriptome analysis [23]. Therefore, we surveyed the NCBI database for the Vg and VgR information, cloned the genes from *B*. *tabaci* Asia1 species and characterized.

The tblastn search of Vg and VgR protein sequences of *D*. *melanogaster*, *N*. *lugens* and *B*. *germanica* (S1 and S2 Tables) against TSA database resulted into the identification of homologous mRNA sequences for each gene. Most of the top blast hit mRNA sequences were common between the blast results for each gene of the above insects. The mRNA sequences, like GAPQ01001336.1, GARQ01026514.1, GARQ01020976.1, GAPQ01015403.1 and GAPP01001385.1 showed homology with Vg; and HP650038.1, HP784971.1, EZ959208.1 and EZ943568.1 with VgR, were selected for our study. The blastx search against NCBI non-redundant protein database further confirmed the homology of these sequences with respective genes. These sequences were used for gene specific primers designing (Table 1) and full length gene sequence of BtA1Vg and BtA1VgR was obtained by RACE and deposited to NCBI (Table 2, S1 and S5 Files).

**Table 2:** Details of whitefly vitellogenin and vitellogenin receptor sequences.

Gene	Accession number	ORF length	Protein Length (AA)	Molecular Mass (kDa)	pI	Cellular localization
**Vitellogenin**	KR818561	6330	2109	239.50	8.68	Nuclear
**Vitellogenin receptor**	KR818562	5430	1809	201.27	4.91	Extracellular

### Vitellogenin

Insect’s Vg synthesizes as big as ~200 kDa precursor protein from 6–7 kb mRNA. After proteolytic cleavage by endoproteases, the precursor protein splits into large (140–190 kDa) and small (40–60 kDa) subunits, which assembled and secreted as 400–600 kDa large oligomeric protein into the hemolymph of insects [5,12]. The cloned *BtA1Vg* was also a large gene with 6330 bp open reading frame, which encoded 2109 amino acid (AA) residue long protein of ~239 kDa calculated molecular mass (Table 2). The blastP search of protein sequence against NCBI-nr protein database showed maximum identity (~70%) with the Vgs sequences of other *B*. *tabaci* species, as expected. The protein consisted of a 28 AA long signal peptide at N-terminus and presumed to be nuclear in nature as detected by SignalP 4.1 and PSORT II Server, respectively. Gene ontology analysis indicated the role of protein as lipid transport (GO:0006869) and lipid transporter activity (GO:0005319) in biological process and molecular function categories, respectively. Similar role of Vg is also reported in earlier studies [35].

ConSurf blast of protein sequence against the known proteins showed the degree of conservation and functional residues. BtA1Vg showed very high degree of conservation with the insects Vgs as already been reported in earlier studies [33]. Several conserved and exposed functional residues were also predicted, which were earlier reported as characteristic feature of insect Vgs (S2 File). Occurrence of polyserine tracts were reported as one of the remarkable feature of insect Vgs. Though, it is mostly present at N-terminus of the Vgs, but in certain insects (like cockroach and mosquito) it was reported from C-terminus also [5]. In BtA1Vg, polyserine tracks were observed at both N and C-terminus of the protein (S1 File). These polyserine regions are expected to serve as phosphorylation sites [12]. These phosphorylated polyserine tracts signify high negative charge, which possibly encourage the binding of vital metal ions (like Ca^2+^ and Fe^3+^) or increase the solubility of the Vg [36,37]. Further, reduced uptake of dephosphorylated Vg is reported by oocytes, which suggested the role of phosphorylated residues in interaction between Vg and VgR on the oocyte surface [38].

Presence of the tetra residue motifs R/KXXR/K, DGXR and GL/ICG are reported as conserved and important feature of Vg. The R/KXXR/K motif acts as consensus cleavage site for subtilisin-like endoproteases, which plays important role in maturation of primary Vg gene product [39]. This motif is mostly found conserved at the N-terminus, but in certain insects (especially in hemimetabola), it is present at C-terminus or sometime in the center also. Further, it was reported to be flanked by polyserine tracts [5,12,13,40,41]. Since *B*. *tabaci* belongs to the hemimetabola group, this motif was present at both N and C-terminus of BtA1Vg, and was flanked by polyserine residues also (S1 File). The DGXR and GL/ICG motifs are conserved at C-terminus part of insect Vgs [5,40] and these motifs were also found in BtA1Vg at similar positions (S1 File). It was earlier presumed that the GL/ICG motif along with the DG residues of DGXR motif and conserved cysteine residues are necessary for proper functioning of Vg during embryo development [40].

**Fig 1:**
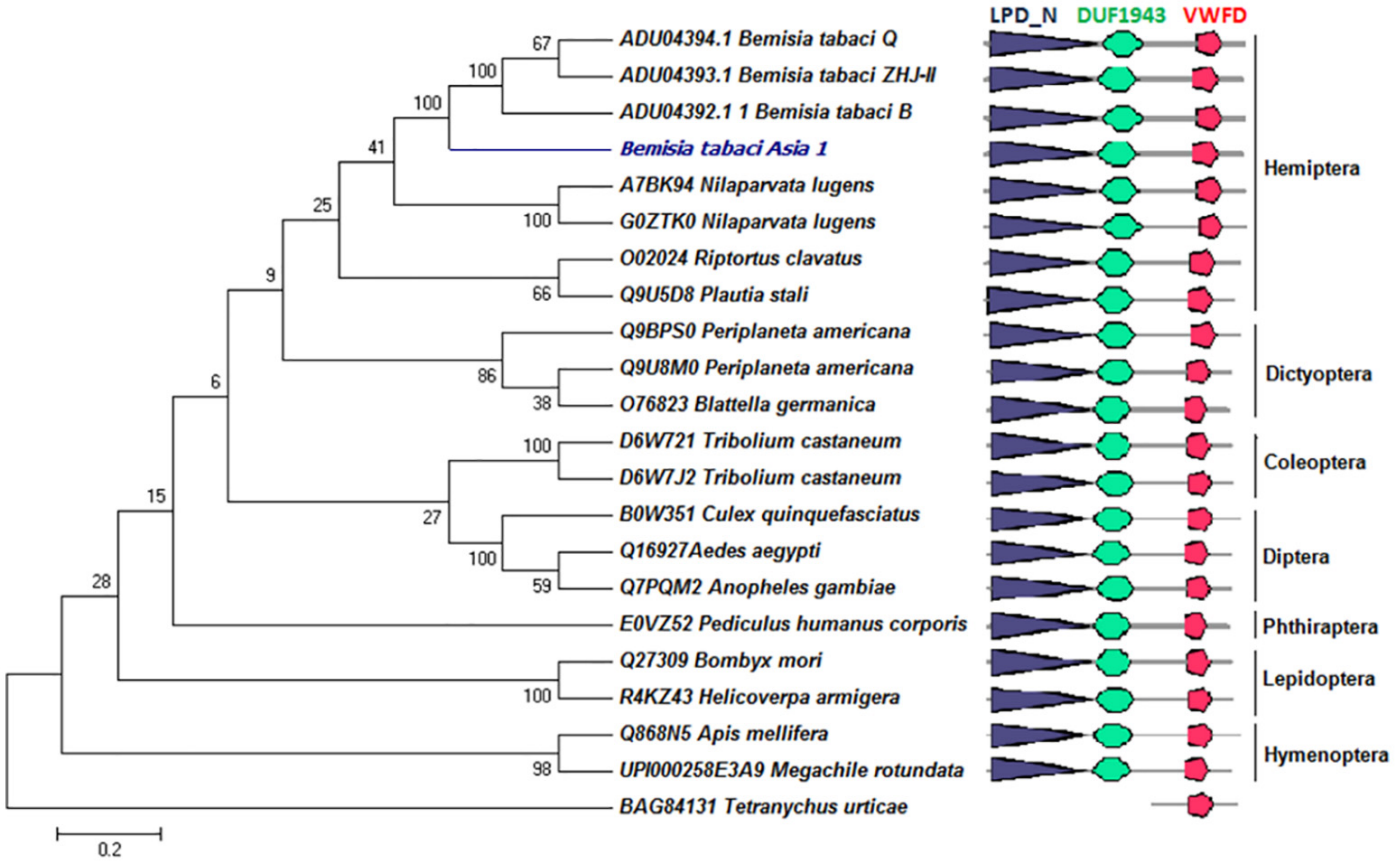
Domain architecture and phylogenetic analysis of insect’s vitellogenins (Vgs). Similar domain architecture is present in insect’s vitellogenins of different orders. Phylogenetic tree was constructed using the sequences of different insect orders. *Tetranychus urticae* (mite) sequence was used as outlier. Figure shows order wise grouping of the insect’s vitellogenins, and BtA1Vg is grouped with other hemipteran insects as expected.

Domain architecture analysis by SMART and Scan-Prosite server confidentially predicted the presence of two major domains viz. vitellogenin/ LPD_N (N-terminal lipoprotein domain, 32–913 AA) and VWFD (Von Willerand Factor type D domain, 1632–1844 AA) and an additional domain DUF1943 (945–1235 AA) in BtA1Vg (Fig 1, Table 3, S3 File). Similar analysis was done with the Vgs sequences from various orders of insects and a mite *Tetranychus urticae*. We found comparable domain organization in insect Vgs (Fig 1). However, only VWFD domain was present in *T*. *urticae*. Scan-Prosite score for vitellogenin (PS51211) and VWFD (PS51233) domains in different insects is shown in S3 Table.

**Table 3:** Confidently predicted domains of *Bemisia tabaci* Asia1 vitellogenin (BtA1Vg) and vitellogenin receptor (BtA1VgR) proteins by SMART server.

Protein	Name	Start	End	E-value
**Vitellogenin (BtA1Vg)**	**Signal peptide**	1	28	NA
	**LPD_N**	32	913	1.41e-281
	**DUF1943**	945	1235	9.93e-21
	**VWFD**	1632	1844	0.0000116
**Vitellogenin Receptor (BtA1VgR)**	**Signal peptide**	1	34	N/A
	**LDLa**	36	74	5.3e-9
	**LDLa**	81	120	1.59e-12
	**LDLa**	122	160	2.73e-11
	**LDLa**	166	207	1.61e-8
	**EGF**	214	247	0.0382
	**EGF_CA**	248	287	8.05e-10
	**LY**	356	398	4.74e-7
	**LY**	403	446	5.08e-7
	**LY**	447	489	3.7e-9
	**LY**	490	531	0.00000933
	**EGF**	560	600	3.71
	**LY**	628	670	47.9
	**LY**	671	713	0.00032
	**LY**	761	805	0.000485
	**EGF**	879	918	0.0063
	**LDLa**	922	959	1.02e-10
	**LDLa**	960	998	3.05e-9
	**LDLa**	1000	1038	3.81e-10
	**LDLa**	1040	1077	1.56e-15
	**LDLa**	1082	1117	3.5e-9
	**LDLa**	1132	1169	9.09e-8
	**LDLa**	1170	1208	2.57e-7
	**LDLa**	1223	1260	0.000263
	**EGF**	1261	1297	0.041
	**EGF_CA**	1298	1337	5.44e-7
	**LY**	1407	1448	0.0252
	**LY**	1450	1499	0.00274
	**LY**	1500	1542	1.33e-10
	**EGF_like**	1616	1650	34.3
	**Transmembrane region**	1678	1700	N/A

**Fig 2:**
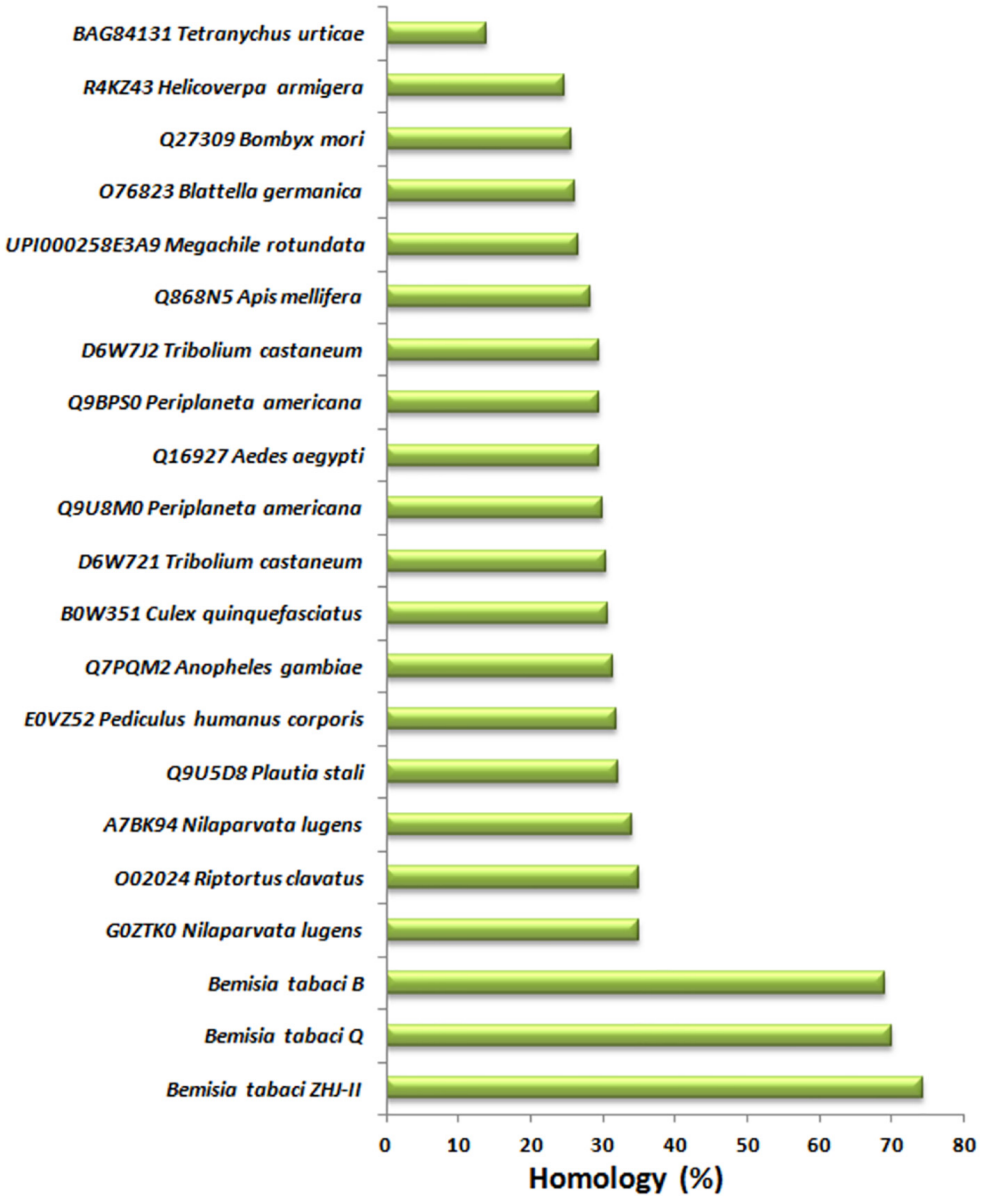
Percent homology of *Bemisia tabaci* Asia 1 vitellogenin with other insects. *B*. *tabaci* Asia 1 vitellogenin shows the highest similarity with the vitellogenins of other *B*. *tabaci* species, followed by other hemipteran insects.

To analyze the percent homology and conservation of different motifs and important amino acids residues in insects Vgs, multiple sequence alignment was performed using the sequences from various orders of insects. BtA1Vg showed the highest homology (74%) with *B*. *tabaci* ZHJII Vg, followed by *B*. *tabaci* Q (70%) and *B*. *tabaci* B (69%) (Fig 2). Further analysis in other insect families showed homology with hemipteran insects like *N*. *lugens* (35%) and *R*. *clavatus* (35%) and the lowest homology was with *Tetranychus urticae* (13.85%). Similar pattern of homology has been earlier reported between the different groups of insects [5].

**Fig 3:**
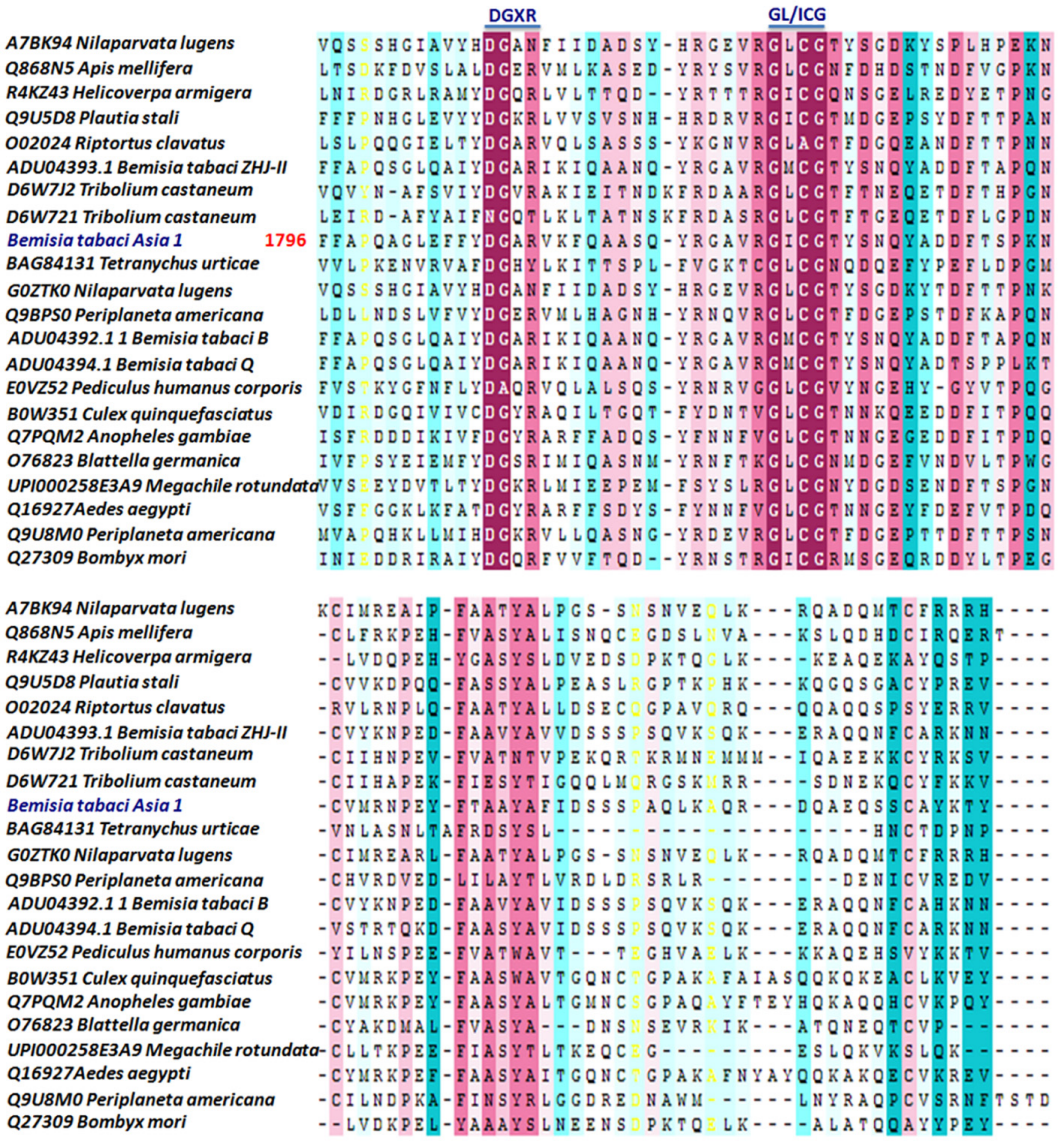
Multiple sequence alignment of the C-terminal part of insect’s vitellogenins (Vgs). Number indicates the amino acid position of *Bemisia tabaci* Asia 1 vitellogenin (BtA1Vg). The conserved DGXR and GL/ICG motifs are underlined.

We found very high degree of conservation in important motifs like DGXR and GL/ICG across the different order of insects (Fig 3) as reported earlier in case of insects and other organisms [5, 42]. Certain amino acids residues like SH-group containing (C), hydrophobic (P), hydrophilic (Y), basic (R) and acidic (D and E), were also highly conserved in insect Vgs, especially at C-terminus (Fig 3, S4 File). It was earlier proposed that the C residue at conserved position help in maintaining the specific structure of DGXR and GL/ICG motifs for appropriate function of protein during development [5]. However the exact roles of other conserved amino acid residues are still not known.

The phylogenetic relationship between the Vgs of 22 different insect species from various orders was studied using the neighbor-joining method with MEGA 6. We performed this analysis using the full length protein sequences, because earlier it was reported that entire Vg sequences show excellent phylogenetic inferences, which is in agreement with the phylogenetic classification of insects [5,13]. All other positions containing gaps and missing data were eliminated. There were a total of 177 positions in the final dataset for evolutionary analyses. The phylogenetic tree clustered the Vgs in order wise manner of the insects as well as clearly demarcated the holo and hemi-metabolous insects Vgs (Fig 1) as demonstrated in earlier studies [5]. The *B*. *tabaci* Vgs were clustered together along with the other hemipteran insects. Similar to the earlier phylogenetic relationship, the current tree also reflected the phylogenetic bounding between insects Vgs, even after the diverse existence.

### Vitellogenin receptor

The Vg accumulation in insect’s oocytes is mediated by a member of the low density lipoprotein receptor (LDLR) family, known as vitellogenin receptor (VgR) [43]. The VgRs are structurally related proteins reported from several vertebrate and invertebrate organisms [6, 44]. Several VgRs are characterized from different insect species like *Drosophila*, *Aedes*, *Spodoptera*, *Nilaparvata* and others [6,44,45]. However, the VgR protein from *B*. *tabaci* was not characterized. Therefore, here we cloned the gene encoding VgR protein from *B*. *tabaci* Asia1 species as described above and characterized. The insects VgRs are synthesized as large ~180–215 kDa proteins, encoded by ~5.5–7 kb long mRNA coding region [6,44]. The isolated *BtA1VgR* mRNA consisted of 5430 bp long open reading frame, which encoded ~201 kDa protein of 1809 AA residues (Table 2, S5 File). The identity of the isolated gene and protein was further confirmed by blastx and blastp search at NCBI database, respectively. The protein showed highest identity with the VgR protein sequence of other *B*. *tabaci* species (ADM34986.1), followed by *Acyrthosiphon pisum* (XP_008180459.1) as expected, due to their similar taxonomic position.

The 34 AA residue long signal peptide was detected at N-terminus of BtA1VgR and it was presumed as extracellular protein similar to the other reported VgRs [44,45]. Gene ontology search predicted the role of protein in molting cycle (GO:0042303) and sterol transport (GO:0015918) in biological process, and calcium ion binding (GO:0005509), protein binding (GO:0005515) and sterol transporter activity (GO:0015248) in molecular function category.

**Fig 4:**
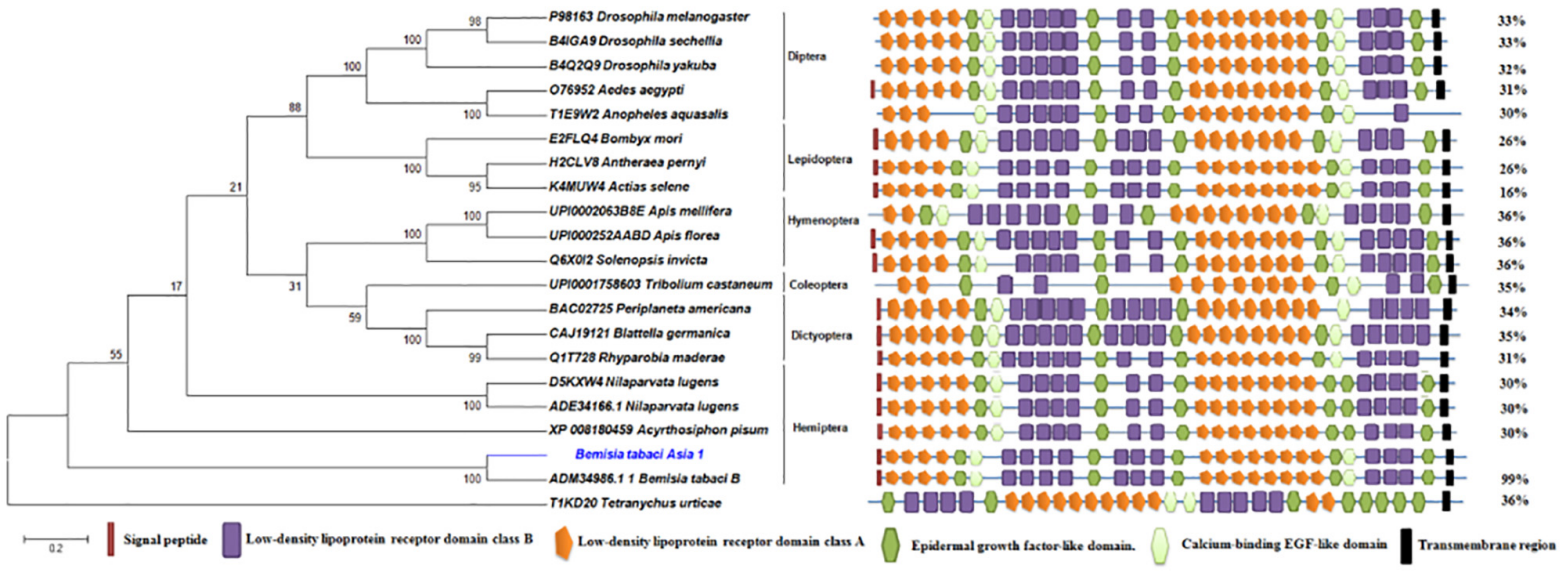
Domain architecture and phylogenetic analysis of insect’s vitellogenin receptors (VgRs). Figure shows comparable domain architecture in insect’s VgRs of different orders. Phylogenetic tree was constructed using the sequences of different insect orders. *Tetranychus urticae* (mite) sequence was used as outlier. Figure shows order wise grouping of the insect’s VgRs, and BtA1VgR is grouped with other hemipteran insects as expected. The values (%) on the right side show the overall identity compared to the BtA1VgR.

The structural organization of insect VgRs consist of five major modular domains (i) low density lipoprotein receptor class A (LDLa) repeats or ligand binding type A repeats (LBRs) with six cysteine residues each, (ii) epidermal growth factor (EGF)-like repeats with/ without calcium binding region, also having six cysteine residues each; (iii) low density lipoprotein receptor class B (LDLb) repeats or YWTD motif containing repeats, (iv) a single transmembrane region and (v) cytoplasmic region containing sequence motifs NPXF [6,12,13,44,45]. Domain architecture analysis by SMART and Scan-Prosite server showed the presence of above described five typical insects VgR specific domains in BtA1VgR also. We observed 12 LDLa domains in two patches, 10 LDLb domains in three patches, 7 EGF domains in between the LDLa and LDLb domains, 1 transmembrane region and a cytoplasmic region at C-terminus (Fig 4, Table 3, S6 File). The domain organization was compared with VgRs of the insects of various orders. We found comparable patterns in each insect VgRs, though the number of different domains was variable (Fig 4). Comparable domains were also present in mite *T*. *urticae*, but the domains arrangement was different from insects. Similar observations have earlier been reported also [6].

ConSurf blast and multiple sequence alignment of the BtA1VgR with the known VgRs of various orders of insect enlightened the depth of conservation of important motifs and functional amino acid residues across the taxa. The BtA1VgR was also found highly conserved across the various orders of insects (S7 and S8 Files) as reported for other insects VgRs [6,44,45]. The domains, conserved motifs and residues of BtA1VgR are highlighted with various colors in S8 File.

The typical characteristic of the presence of six cysteine residues in each LDLa was also observed in BtA1VgR. These residues are reported to be involved in di-sulfide bond formation in C1–C3, C2–C5, and C4–C6 fashion to maintain the structure of protein [17]. Another motif of cluster of acidic residues (CDxxxDCxDGSDE) was also found conserved in BtA1VgR between the 4-6th cysteine of each LDLa domain. Fass et al. [46] reported that these acidic clusters are involved in chelating the calcium ions, which play critical role in folding LDLa domains [46,47,48].

Similar to the other insects VgRs, BtA1VgR consisted of seven EGF domains, three of them were single and four in the group of two (Fig 4). Each EGF domain contained six conserved cysteine residues, which are also reported to be involved in di-sulfide bond formation as in case of LDLa. However the pattern of bonds (i.e. C1–C3, C2–C4, and C5–C6) differs in both the domains [49].

The LDLb domains or YWTD repeats containing domains are known to form β-propeller structure by involving 6 repeats of YWXD motif. This motif could also be detected in BtA1VgR in each LDLb domains except first domain, where it was YFTD in BtA1VgR as well as in most of the aligned insects VgR sequences (S8 File). It has been reported in earlier studies also, that the YWXD motif usually absent in first repeat [6].

Most of the insect VgRs consist of a short stretch (~30 AA residues) of S and/or T residue enriched O-linked sugar domain (OLSD) immediately outside of the plasma membrane [8,12,13]. We observed 27 AA residues long OLSD in BtA1VgR, which consisted of eight S and T (four each) residues (S6 File). A similar number of such residues were also present in other insect VgR sequences (S8 File) except *Drosophila*, in which the OLSD is reported as absent [10].

The BtA1VgR consisted of 23 AA residues long transmembrane domain (TMD) enriched by several hydrophobic residues like glycine (G), alanine (A), leucine (L), isoleucine (I), phenylalanine (F), and valine (V) (Table 3, S6 and S8 Files). Similar TMD is reported from other insects also [6]. The TMD forms α-helical structure and holds the VgR in the plasma membrane [19].

The insect VgRs and other LDLR family receptor possess a cytoplasmic domain with NPXY or LL/I or other variant of NPXY motif as an internalization signal [12,13,32,50]. The BtA1VgR consisted of NPAF, NPLQ and LI motifs (S6 and S8 Files), which probably act as internalization signal. The NPAF and NPLQ motifs are earlier reported for the similar function in *Drosophila* and *Bombyx mori* [36,51].

The evolutionary relationship of BtA1VgR with other insects VgRs was inferred using the neighbor-Joining method in MEGA 6 as described above [25]. The analysis involved 21 amino acid sequences from various order of insects. All positions containing gaps and missing data were eliminated. There were a total of 746 positions in the final dataset for the analysis. BtA1VgR was grouped with the VgR of *B*. *tabaci* B, and was in the proximity of other hemipteran insects VgRs (Fig 4). This might be due to their similar taxonomic status. Comparably, other VgRs sequences were also clustered as per the taxonomic position of the related insects. Similar evolutionary relationship between insect VgRs is reported in other studies also [44,45]. The phylogenetic tree reflected the evolutionary bounding between the VgRs of the different groups of insects.

### Transcript expression analysis in developmental stages

**Fig 5:**
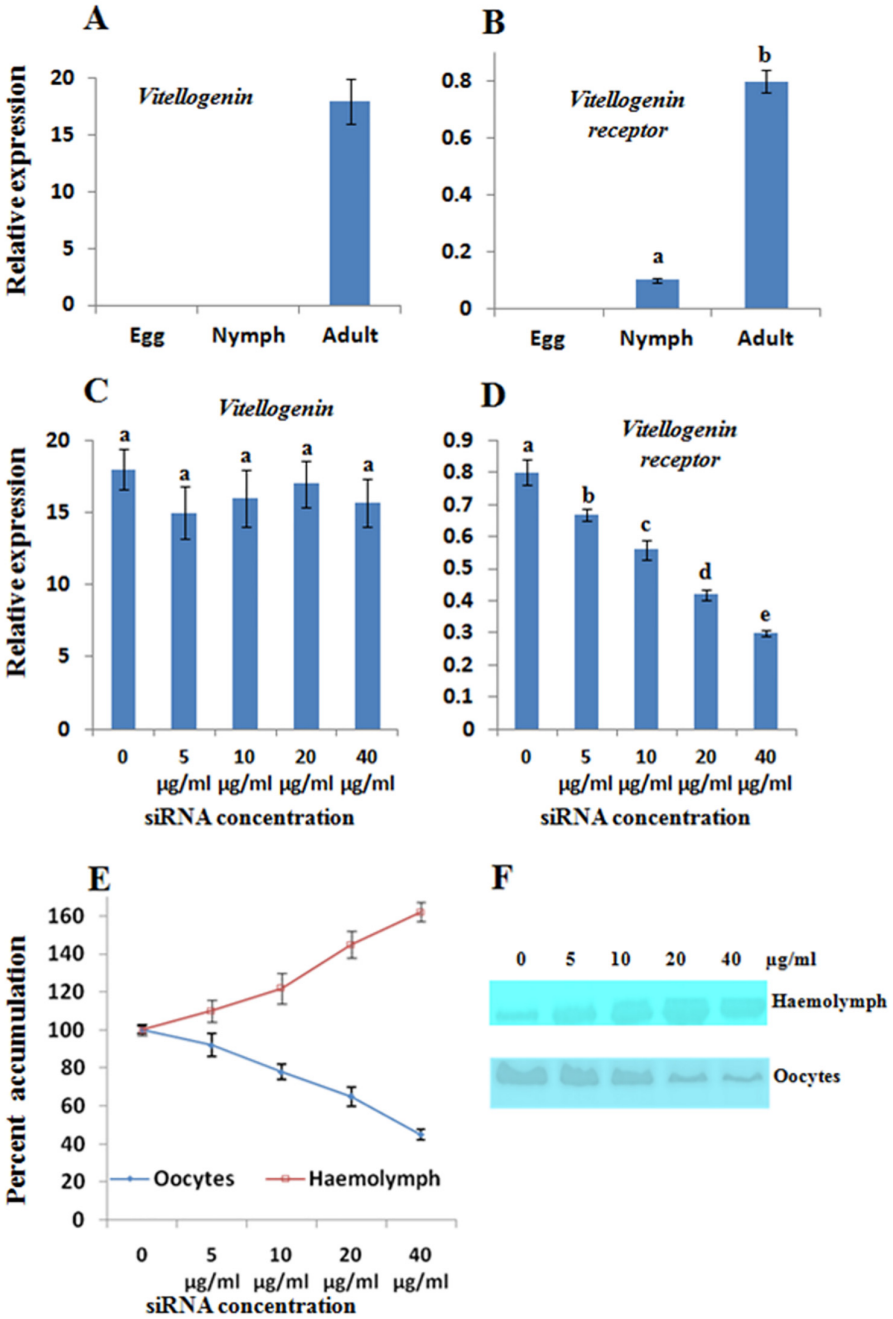
Expression analysis of BtA1Vg and BtA1VgR. The transcript expression was normalized with the *actin* expression. (A) Transcript expression of *BtA1Vg* in egg, nymph and adult. (B) Transcript expression of *BtA1VgR* in egg, nymph and adult. (C) Transcript expression of *BtA1Vg* in adult insects after 3 days of feeding of various concentration of siRNA. (D) Transcript expression of *BtA1VgR* in adult insects after 3 days of feeding of various concentration of siRNA. (E) Comparative accumulation of BtA1Vg protein in oocyte and haemolymph of adult insects after 3 days of feeding of various concentration of siRNA. The normal quantity was considered as 100% and relative accumulation was calculated in percent change. All the experiments were performed in three biological replicates and statistical analysis was performed. The different letters above each bar indicates significantly different data according to DMRT, preceded by a one-way ANOVA having p-value<0.05.

Expression analysis of *BtA1Vg* and *BtA1VgR* mRNA was performed during egg, nymph and adult stage by real time PCR. The level of expression was normalized with the *actin* mRNA, an internal control [26]. There was a very high expression of *BtA1Vg* mRNA in adult insects, which was ~18 fold higher than the *actin* mRNA (Fig 5a). However, we could not detect any expression in egg and nymph stage. The result was as per expectation, because Vg is usually synthesized in fat body, transported to the haemolymph and then to the oocytes [32], therefore *BtA1Vg* mRNA expression was not required in egg and nymph stages. Similar expression pattern is reported in other insects as well [52]. The *BtA1VgR* mRNA expression was detected in nymph and adult insects, however it was deficient in eggs (Fig 5b). The expression in adults was ~8 fold higher than the nymphs. Since, VgRs are localized on the surface of oocytes, the expression in adult was usual. Moreover, the different developmental stages of nymphs were collected together for expressions analyses; therefore, we could detect some expression of *BtA1VgR* in nymph as well. It is reported that the expression of VgR started at later stages of nymphs in several insects [44,45].

### Silencing of vitellogenin receptor established interaction with vitellogenin

We opted an indirect method of interaction analysis of BtA1Vg and BtA1VgR by reducing the expression of *VgR* using siRNA. Since the transportation of Vg from haemolymph to oocytes is completely dependent upon the mediation of VgR [6], we presumed that the reduced expression of VgR might directly affect the accumulation of Vg in oocytes. Further, we have earlier reported significant RNA interference activity in *B*. *tabaci* after oral delivery of different siRNA molecules [22]. Therefore we fed different quantity of *BtA1VgR* siRNA to *B*. *tabaci* for three days, and then analyzed the expression of *BtA1VgR* and *BtA1Vg* transcripts, and accumulation of BtA1Vg protein in haemolymph and oocytes. We found a decreasing trend in expression of *BtA1VgR* with increase in concentration of siRNA. About 50% decrease in *BtA1VgR* mRNA level was observed with 40 μg/ml concentration of siRNA (Fig 5d). Similar trend has been earlier reported in case of other genes [22]. However, feeding of *BtA1VgR* siRNA did not affect the mRNA level of *BtA1Vg* (Fig 5c), but significantly affected the accumulation of BtA1Vg protein in oocytes (Fig 5e). The transportation of BtA1Vg protein was affected as presumed. Therefore, we observed the decreased quantity of BtA1Vg in oocytes, and increased quantity in haemolymph (Fig 5e and 5f). The results supported the role of BtA1VgR in transportation of BtA1Vg from haemolymph to oocytes. Further, increased concentration of BtA1Vg in haemolymph and almost similar expression of BtA1Vg mRNA, indicated that the expression of *BtA1Vg* gene is not regulated by feedback inhibition mechanism.

### Silencing of vitellogenin receptor reduced survival and fecundity of *B*. *tabaci*

**Fig 6:**
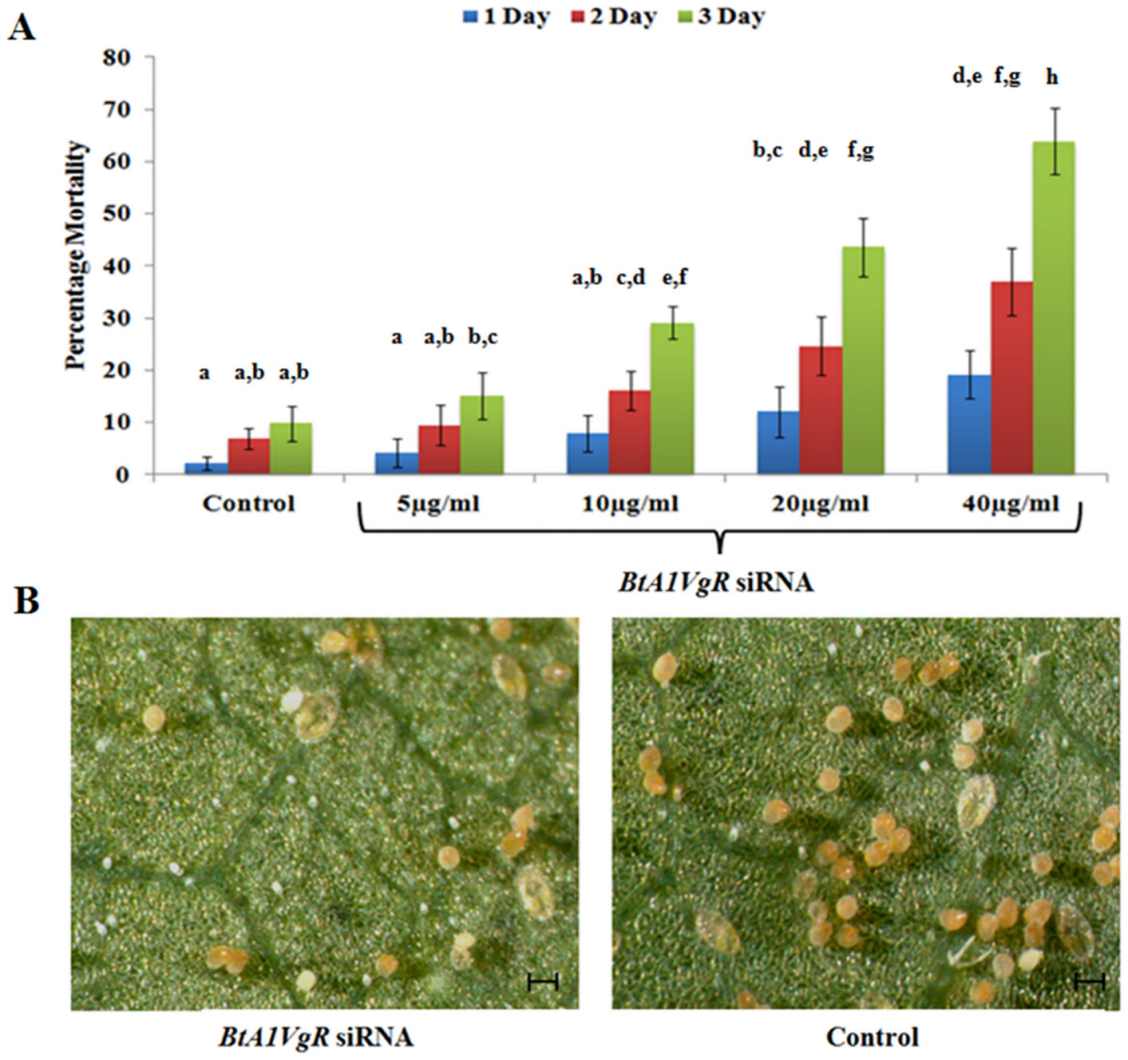
Insect Bio-assay and fecundity analysis in *BtA1VgR* siRNA fed *B*. *tabaci*. (A) Percentage mortality of insects after feeding of various concentration of *BtA1VgR* siRNA at different time interval. (B) Light microscope imaging of cotton leaf after 20 days of releasing of control and *BtA1VgR* siRNA fed insects. The different letters above each bar indicates significantly different data as per DMRT, preceded by a one-way ANOVA having p-value<0.05.

RNAi is reported as an effective tool for the control of *B*. *tabaci* infestation on plants, which is probably due to the high expression of its machinery [22,26,27]. Further, Vgs and VgRs are related to the fecundity of insects [6]. Therefore, survival and fecundity of *B*. *tabaci* was also analyzed after feeding of various concentration of *BtA1VgR* siRNA. Silencing of *BtA1VgR* significantly decreases the survival rate of insects. Percentage mortality of insects was increasing as the concentration of siRNA increases. After three days, percentage mortality of insects was 15.21±4.49, 29.11±3.03, 43.58±5.64 and 63.83±6.35 in 5, 10, 20 and 40 μg/ml concentration of siRNA, respectively, where as only 9.87±3.36% mortality was observed in control (Fig 6A). After three days of bioassay, the surviving insects of control and *BtA1VgR* siRNA (40 μg/ml) were released on cotton plant and analyzed after 20 days. Severe reduction in egg count (67.74±12.55%) and distorted egg laying pattern was observed on the leaves containing *BtA1VgR* siRNA fed insects (Fig 6B). The results suggested that these genes can be targeted for the control of *B*. *tabaci* on crop plants through RNAi.

## Conclusions

In the present study, we cloned and characterized the genes encoding Vg and VgR proteins of *B*. *tabaci* Asia1. We demonstrated that both the protein showed comparable domain organization and conservation to the related proteins of other insects from various orders. Most of the important structural and functional motifs and amino acids residues were found conserved in the BtA1Vg and BtA1VgR also, as reported in earlier studies. The phylogenetic analysis showed the evolutionary relationship of both the proteins with their homologs across the taxa, and showed similar pattern as they are related in the taxonomic potions. The mRNA expression pattern was also conserved for both the genes in insect, we found higher expression of both the genes in adult insects, as they play critical role in oocyte and embryo development. Silencing of *BtA1VgR* by siRNA established the interaction of these two proteins and their essential role in survival and fecundity in *B*. *tabaci*. Present study enlightens several aspects of molecular characterization of BtA1Vg and BtA1VgR. Since these are essential proteins for insect development, siRNA mediated silencing of these genes can be useful in controlling the *B*. *tabaci* when expressed in crop plants.

## Supporting Information

S1 Fig Identification of vitellogenin by various methods of staining and MS/MS analysis. Figure shows the (A) Coomassie Brilliant Blue, (B) Methyl Green, (C) Schiff’s reagent and (D) Sudan Black B stained gel of total protein extracted from adult *Bemisia tabaci*. The arrow head shows the band of vitellogenin. Figure (E) shows the peptide sequences obtained by MS/MS analysis of vitellogenin.
(TIF)

S1 Table List of vitellogenin sequences used in blastx. (DOC)

S2 Table List of vitellogenin receptor sequences used in the present study. (DOC)

S3 Table Scan-Prosite score for common domains of vitellogenin in selected insects. (DOC)

S1 File Nucleotide and protein sequence of vitellogenin with the characteristic features highlighted in various colours. Poly serine tracts, consensus subtilisin-like endoproteases cleavage sites R/KXXR/K, GL/ICG motif and DGXR motif are highlighted by yellow, green, blue and purple colours, respectively.
(DOC)

S2 File ConSurf blast result of whitefly (Bemisia tabaci Asia1) vitellogenin. (DOC)

S3 File Protein sequence whitefly (Bemisia tabaci Asia 1) vitellogenin with colour demarcation of important domains. Blue and red colour show vitellogenin (PS51211) and VWFD (PS51233) domains, respectively. N-terminus signal peptide is bold and underlined.
(DOC)

S4 File Multiple sequence alignment of full length vitellogenin protein sequences of selected insects from each order. LPD_N, DUF1943, VWFD domains are underlined by blue, green and red colours, respectively.
(PDF)

S5 File Nucleotide and protein sequence of vitellogenin receptor of Bemisia tabaci Asia 1. (DOC)

S6 File Protein sequence whitefly (Bemisia tabaci Asia 1) vitellogenin receptor with colour demarcation of important domains. Orange, green, red, blue and purple colour fonts show LDLa, EGF, calcium binding EGF, LDLb and O-Linked sugar domains, respectively. N-terminus signal peptide is bold and underlined. Transmembrane and cytoplasmic regions are highlighted in gray and yellow colours, respectively.
(DOC)

S7 File ConSurf blast result of whitefly (Bemisia tabaci Asia 1) vitellogenin receptor. (DOC)

S8 File Multiple sequence alignment of full length vitellogenin receptor protein sequences of selected insects from each order. Important domains are labeled and highlighted.
(PDF)
