# Supplementary figures and images for: Molecular Characterization of Vitellogenin and Vitellogenin Receptor of Bemisia tabaci
Source: PLoS One. 2016 May 9;11(5):e0155306. doi: 10.1371/journal.pone.0155306 (PMC4861306; doi:10.1371/journal.pone.0155306)

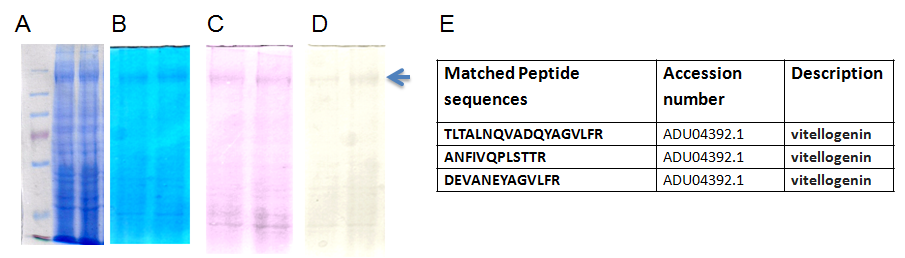

Supplement: S1 Fig — Figure shows the (A) Coomassie Brilliant Blue, (B) Methyl Green, (C) Schiff’s reagent and (D) Sudan Black B stained gel of total protein extracted from adult Bemisia tabaci. The arrow head shows the band of vitellogenin. Figure (E) shows the peptide sequences obtained by MS/MS analysis of vitellogenin. (TIF) [file pone.0155306.s001.tif]
