# Supplementary material for: Molecular Characterization of Vitellogenin and Vitellogenin Receptor of Bemisia tabaci
Source: PLoS One. 2016 May 9;11(5):e0155306. doi: 10.1371/journal.pone.0155306 (PMC4861306; doi:10.1371/journal.pone.0155306)
Supplement: S1 Table — (DOC) [file pone.0155306.s002.doc]

**S1 Table**. List of vitellogenin sequences used in blastx.

| **S. No.** | **Insect** | **Accession No.** |
| --- | --- | --- |
| **1** | *Blattella germanica* | CAA06379 |
| **2** | *Nilaparvata lugens* | BAF75351 |
