# Supplementary material for: Molecular Characterization of Vitellogenin and Vitellogenin Receptor of Bemisia tabaci
Source: PLoS One. 2016 May 9;11(5):e0155306. doi: 10.1371/journal.pone.0155306 (PMC4861306; doi:10.1371/journal.pone.0155306)
Supplement: S3 Table — (DOC) [file pone.0155306.s004.doc]

**S3 Table**. Scan-Prosite score for common domains of vitellogenin in selected insects.

| **Insects** | **Domains** | |
| --- | --- | --- |
|  | **Vitellogenin (PS51211)** | **VWFD (**[**PS51233**](http://prosite.expasy.org/cgi-bin/prosite/nicedoc.pl?PS51233)**)** |
| ***Bemisia tabaci Asia 1*** | 38.16 | 22.27 |
| ***ADU04394.1 Bemisia tabaci Q*** | 32.90 | 19.76 |
| ***ADU04392.1 1 Bemisia tabaci B*** | 38.50 | 25.58 |
| ***ADU04393.1 Bemisia tabaci ZHJ-II*** | 38.82 | 25.78 |
| ***Q16927Aedes aegypti*** | 38.8 | 23.04 |
| ***Q9U8M0 Periplaneta americana*** | 42.75 | 29.69 |
| ***Q9BPS0 Periplaneta americana*** | 41.04 | 26.38 |
| ***Q868N5 Apis mellifera*** | 42.94 | 30.47 |
| ***Q27309 Bombyx mori*** | 36.42 | 23.77 |
| ***A7BK94 Nilaparvata lugens*** | 36.13 | 14.93 |
| ***B0W351 Culex quinquefasciatus*** | 36.99 | 28.03 |
| ***D6W721 Tribolium castaneum*** | 38.54 | 22.13 |
| ***D6W7J2 Tribolium castaneum*** | 36.65 | 19.10 |
| ***E0VZ52 Pediculus humanus corporis*** | 38.31 | 22.48 |
| ***G0ZTK0 Nilaparvata lugens*** | 36.03 | 25.86 |
| ***O02024 Riptortus clavatus*** | 42.56 | 34.11 |
| ***O76823 Blattella germanica*** | 35.64 | 29.38 |
| ***Q7PQM2 Anopheles gambiae*** | 37.26 | 29.97 |
| ***Q9U5D8 Plautia stali*** | 35.62 | 34.10 |
| ***R4KZ43 Helicoverpa armigera*** | 30.09 | 22.50 |
| ***UPI000258E3A9 Megachile rotundata*** | 25.88 | 31.04 |
| ***BAG84131 Tetranychus urticae*** | -- | 22.79 |
