# Supplementary material for: Molecular Characterization of Vitellogenin and Vitellogenin Receptor of Bemisia tabaci
Source: PLoS One. 2016 May 9;11(5):e0155306. doi: 10.1371/journal.pone.0155306 (PMC4861306; doi:10.1371/journal.pone.0155306)
Supplement: S1 File — Poly serine tracts, consensus subtilisin-like endoproteases cleavage sites R/KXXR/K, GL/ICG motif and DGXR motif are highlighted by yellow, green, blue and purple colours, respectively. (DOC) [file pone.0155306.s005.doc]

**Supplementary file 1.** Nucleotide and protein sequence of vitellogenin with the characteristic features highlighted in various colours. Poly serine tracts, consensus subtilisin-like endoproteases cleavage sites R/KXXR/K, GL/ICG motif and DGXR motif are highlighted by yellow, green, blue and purple colours, respectively.

| 1 atgattccagtaagagacttctcctcttctgtgatcatgtggact  M I P V R D F S S S V I M W T  46 cccgctttattgtgcctgctggtcgcagccgccaatgcccaatac  P A L L C L L V A A A N A Q Y  91 gggtggaaaaatggaaacctctacaaatacgaaatcaacggacgc  G W K N G N L Y K Y E I N G R  136 accctgaccgccctgaaccaagtcgccgaccaatacgccggagtc  T L T A L N Q V A D Q Y A G V  181 ttattcagagccaacttctacgtccaacccttctccagtgacaga  L F R A N F Y V Q P F S S D R  226 ctgtctgcctacatccaaaatgccgagaccgctcaagttcacgct  L S A Y I Q N A E T A Q V H A  271 gagctgccaagtggatatgaatctcacatcccctccagccagttg  E L P S G Y E S H I P S S Q L  316 aactacaagagtatgcccctcagccacgagccattcgaaatttac  N Y K S M P L S H E P F E I Y  361 ctgaagaagggagtcgtctccaaccttcgcgtcaacaagaatgtc  L K K G V V S N L R V N K N V  406 tccgactgggaacttaacatcatcaaggctgttgtgagccaaatc  S D W E L N I I K A V V S Q I  451 caagttgacacccaaggtcaaaacttgaagaaatccagccacaac  Q V D T Q G Q N L K K S S H N  496 caactccccaaggaaaacaagccctacggtgtttacaagaccatg  Q L P K E N K P Y G V Y K T M  541 gaagactccgtcaccggtgaatgtgaaactctctacgatgtctca  E D S V T G E C E T L Y D V S  586 cctctgccagaaatcaccctccaaaccaaaccctggttggttcct  P L P E I T L Q T K P W L V P  631 ttccccaacttccgtgaaaacggacaattcatcgacatcgtcaag  F P N F R E N G Q F I D I V K  676 accaccaactacagcaaatgtgaagaacgttctgcttaccacttc  T T N Y S K C E E R S A Y H F  721 ggtatcactggtctgaccaactggaaacccgccagcaaccaaatg  G I T G L T N W K P A S N Q M  766 ggacaattcctctcccgctccaacatcaaccgtgtcgtcatttct  G Q F L S R S N I N R V V I S  811 ggaaacgtgaaatactacaccatccaatcctctgtttccaccaac  G N V K Y Y T I Q S S V S T N  856 aaaatcgtcatcagtccacagatgtacgaatcacagaagggaatg  K I V I S P Q M Y E S Q K G M  901 gtcatcagtgtcatgaacatgaccctggcttccttccaccaagcc  V I S V M N M T L A S F H Q A  946 aatggatctccccgcagcgtcaacaactaccgcaaggtcaacaac  N G S P R S V N N Y R K V N N  991 ttagtctacgactacatggccgcttctcctaacgcatacgctcaa  L V Y D Y M A A S P N A Y A Q  1036 cactacaacaacaacggtgcttccagcagctcctccagctccagc  H Y N N N G A S S S S S S S S  1081 tctgactcttccagctctagctccagctccagcagcagctcttcc  S D S S S S S S S S S S S S S  1126 tcttccagctccagcagcagctcctccagctccgaagaagaatac  S S S S S S S S S S S E E E Y  1171 taccgcaacaagaactacaacaacaagcacaacaacaatgccaac  Y R N K N Y N N K H N N N A N  1216 aacaacgacaacaacaacaagaacgaaaacaaccaccacaacgga  N N D N N N K N E N N H H N G  1261 gatgccaacgcctctcgcaaccgttcccgcagagacttgtctcaa  D A N A S R N R S R R D L S Q  1306 tacaacaatggaaacaacaacaacaacggaaacaacgacaatgac  Y N N G N N N N N G N N D N D  1351 gccgaatacgaaaagcgcaacggccacaacggccacaacggccac  A E Y E K R N G H N G H N G H  1396 aacggccacaacggccacaacggccacaacggaaagaacgtcgac  N G H N G H N G H N G K N V D  1441 ggtagctccagctccagcagctccgaggaaaacgaccgttacaac  G S S S S S S S E E N D R Y N  1486 aacggcaaattcgccagctttgcccgccaccacggatctggatcc  N G K F A S F A R H H G S G S  1531 tcctcctccagcagctcctctgactcctccgactcttccagctcc  S S S S S S S D S S D S S S S  1576 tccagctcctctagctcctccagctcttccagctcctcatccagc  S S S S S S S S S S S S S S S  1621 tctgaagacaacagctcctttggatcttccgtctccagcagcagc  S E D N S S F G S S V S S S S  1666 gaagaggactatgaaccacgtccaagcatgtacaaggctcctcaa  E E D Y E P R P S M Y K A P Q  1711 actcctttcttcccctacttcatcggaaactacggtaacagtatc  T P F F P Y F I G N Y G N S I  1756 caatccgctaaacaagttaacggagttgctcttgcccgtaagctc  Q S A K Q V N G V A L A R K L  1801 gcccaagaaattgctgaggaattgaacgacccacgtcaaattact  A Q E I A E E L N D P R Q I T  1846 caaaaaagcactttggctaaattcaacatgttggttgaggaactc  Q K S T L A K F N M L V E E L  1891 agaaccctggacgcgaaacaaatggaacaagcttcccaagagctt  R T L D A K Q M E Q A S Q E L  1936 catttcaactccgcccaggcctccagccacagccgtcaagatgct  H F N S A Q A S S H S R Q D A  1981 ctgaaatctctcgcctggaaatccttctgtgatgccttagttgaa  L K S L A W K S F C D A L V E  2026 gccggtaccggacctgcctttttgcaaatccaaaagatcattgaa  A G T G P A F L Q I Q K I I E  2071 caccaacaagtctccgacgccgaagccgctcgcatgatcagccgt  H Q Q V S D A E A A R M I S R  2116 cttccagtcaccgctcgtttcccagacaaggaatacatgaactct  L P V T A R F P D K E Y M N S  2161 ttcttcaactttgtcagatccaacaatgttcaacaccagaaccaa  F F N F V R S N N V Q H Q N Q  2206 ctcaacgaaactgctctccttgctttcgctgagctttgccgtaaa  L N E T A L L A F A E L C R K  2251 gccgatgtcaacgccagaaacgcccacaactactaccctgtccac  A D V N A R N A H N Y Y P V H  2296 gtttatggacgtgtcctcccagagcatgccaaggctgttgctcac  V Y G R V L P E H A K A V A H  2341 caataccttccctactatgaacaaaacctcaagagagccgttgcc  Q Y L P Y Y E Q N L K R A V A  2386 aacggtgacagccgcaagatccaagcttacatccgtgccattgga  N G D S R K I Q A Y I R A I G  2431 aactttgctcaccccaagatcctcgaagttttcgagccctacctt  N F A H P K I L E V F E P Y L  2476 gaaggaaaggtcccaatctccaacttccaacgcactgtcatggtc  E G K V P I S N F Q R T V M V  2521 ctctcccttaacgaacttgcacgcgtctaccccaaccttgcccgc  L S L N E L A R V Y P N L A R  2566 aatgtcctcttcaagatctaccaaaacacccaagaaaaccaagaa  N V L F K I Y Q N T Q E N Q E  2611 gtccgtgttgccgctgtcttcttaatcttcggaaccaacccatct  V R V A A V F L I F G T N P S  2656 gcccagaccctccaacgtatggctcaattcacctacgaagaccag  A Q T L Q R M A Q F T Y E D Q  2701 gaccaacaggtcaacgctgccgtctcctctgccatccgcaacgcc  D Q Q V N A A V S S A I R N A  2746 gccaagaaatccgctggaatccgcgaagaactcgctgaagccgcc  A K K S A G I R E E L A E A A  2791 caatccgccgtcgatcttcttaaccccaagacctacgggctccaa  Q S A V D L L N P K T Y G L Q  2836 ttctccaagaagtggctccgtgactacattgtcaaagaagaaaac  F S K K W L R D Y I V K E E N  2881 cttgcttacagtgtttacgctgacaccatccaaggcgatgactct  L A Y S V Y A D T I Q G D D S  2926 ctattccccaatcaatattatgccgctttcttccgtcatgttgga  L F P N Q Y Y A A F F R H V G  2971 ggattcaacaaacgcgttgcttccttccgtgctttcgcctctagt  G F N K R V A S F R A F A S S  3016 gctagtgacttgtacgacagagttgctgactccttctacttcgcc  A S D L Y D R V A D S F Y F A  3061 gaacaataccaagacaagtccttcgagaaattctccaagtactct  E Q Y Q D K S F E K F S K Y S  3106 gccgaagaaatcttcaagaacttcaacttcaaggctgactacccc  A E E I F K N F N F K A D Y P  3151 aaggaattggaagcttacttccaatactacttcttaggatccaag  K E L E A Y F Q Y Y F L G S K  3196 caatactccttcatcaacgaggaaatcttcaaccaaatccccaga  Q Y S F I N E E I F N Q I P R  3241 gatttggaaagtgctcttaacaaggctgccaacggatactccttc  D L E S A L N K A A N G Y S F  3286 aacaacaccaagttctacaacgacttcgctcttaccattggattc  N N T K F Y N D F A L T I G F  3331 ccaaccgccaccggtcttcccttctcctacaccatcaagctccca  P T A T G L P F S Y T I K L P  3376 actctcctcaacttcggaggagaagttaaggctaaggtccaagga  T L L N F G G E V K A K V Q G  3421 ttcaaggctgacaacaacaagttccgcatccccgaagccgtcaat  F K A D N N K F R I P E A V N  3466 gttaccgctgccatcgatgtcacttactccaccaagttggaaacc  V T A A I D V T Y S T K L E T  3511 aagttcggattcgttactccattcgaccaccaacgctacgttgcc  K F G F V T P F D H Q R Y V A  3556 ggagttgacaagaacatcaacttcaacctcccccttaagttcaac  G V D K N I N F N L P L K F N  3601 gtcaacctcgatgtttacaacaccaaggctgaaatcatcgtcaag  V N L D V Y N T K A E I I V K  3646 ccattgaacaaccaacacgaacaaagagtcttccactacagctcc  P L N N Q H E Q R V F H Y S S  3691 tacccctacaccgctttctacagcatcttcgacttcgctcctgtc  Y P Y T A F Y S I F D F A P V  3736 caattcaacaagaacatgaagaagatccaaaccaacaaccacaag  Q F N K N M K K I Q T N N H K  3781 aacgaatacaaccaagcctttggaaatgacaaattcggactcaac  N E Y N Q A F G N D K F G L N  3826 ttccgtgccaactacaagggagactaccaatactttgatttcgct  F R A N Y K G D Y Q Y F D F A  3871 actttctacaactacttccaacgcaacgacctcgtcaccttcttc  T F Y N Y F Q R N D L V T F F  3916 ttctacccatgggctgaacaagaaatcaagcaaaacgacttcaac  F Y P W A E Q E I K Q N D F N  3961 ttctacttcaacccatctgcttctgacaacaaggccgccaaattc  F Y F N P S A S D N K A A K F  4006 accttcaactacgcctccaaatacgccgctaaggaacaagccgac  T F N Y A S K Y A A K E Q A D  4051 cacgagagcagaaacgccaacaccaacgatgccgtcacctctaac  H E S R N A N T N D A V T S N  4096 aacaagcccgacagcgaagaaagattgaacgaattcgtccgcaaa  N K P D S E E R L N E F V R K  4141 tcctacgccggaatcaacagtgccttcgtcaacgccttcgacttc  S Y A G I N S A F V N A F D F  4186 tctgcccaattccttggacaaaaggaagccgactacgtttgcacc  S A Q F L G Q K E A D Y V C T  4231 tttgccttcgcccgcagcccagttgctgagaaatctcgtttcctc  F A F A R S P V A E K S R F L  4276 ttctacggacactacaacactgccaacaacaagaaacaacagtgc  F Y G H Y N T A N N K K Q Q C  4321 gctttccacgcctccgctgagatgcccaatgttccattgaccaac  A F H A S A E M P N V P L T N  4366 cctgctgccgccatgaaggctgagccagcatccaagatctacgct  P A A A M K A E P A S K I Y A  4411 aacttcaagttcggagaatctttcgaaaatgccgccaaggttcac  N F K F G E S F E N A A K V H  4456 ttcaacgctaacttgaagcagagctctgaacgccgtcaattcctc  F N A N L K Q S S E R R Q F L  4501 cgcaacaacgccctttacaagcaatgcgaatccgaaatggaacgt  R N N A L Y K Q C E S E M E R  4546 ggacaatacttcctccctgcttgccgtaacttcaccgttgctgac  G Q Y F L P A C R N F T V A D  4591 aacagaatgaacgaatactactacaacttcaacttccaaaacatc  N R M N E Y Y Y N F N F Q N I  4636 cctgaatacttcaagaactacacctaccaagccttcgccttcgcc  P E Y F K N Y T Y Q A F A F A  4681 cgtcacatgggataccaataccaaagcgaaaacgtcgtcaaccca  R H M G Y Q Y Q S E N V V N P  4726 cactacaaacccaacgaaattgaaggattcttcaagttctcaccc  H Y K P N E I E G F F K F S P  4771 agcttcagatacgccaacttctctttcgcctctcccgccttatcc  S F R Y A N F S F A S P A L S  4816 gctgccttcgacaatgtccccgtaaacccatacttcgccgccatc  A A F D N V P V N P Y F A A I  4861 ttcgctccccacccaacttacaccgccttcgacttcttcatgcaa  F A P H P T Y T A F D F F M Q  4906 gaaactttcagatccaaataccaagctgcctgtgtcgctgacaag  E T F R S K Y Q A A C V A D K  4951 ggattcgccaccacttttgacaacagaactttccccgctcacttc  G F A T T F D N R T F P A H F  4996 caaaacaactggtacgtcctcatggcctacatgaacagaaacaac  Q N N W Y V L M A Y M N R N N  5041 tactacaacaacaacttcaaccaatacctccaacaaaacaagaac  Y Y N N N F N Q Y L Q Q N K N  5086 caacacagctacagagactacaacgagaagagattctactccgct  Q H S Y R D Y N E K R F Y S A  5131 gtcctcgccagagacaacagtcacggccaaaaggaattgaaggtt  V L A R D N S H G Q K E L K V  5176 gtcctgaacaacggtgaatacgaattcaacttcgaacccgcctcc  V L N N G E Y E F N F E P A S  5221 caaaacgctggattctccaactccttctccgctagcaaccccgcc  Q N A G F S N S F S A S N P A  5266 gccaaggtccaattcaacaaggaagaacaacacgtccaatacaag  A K V Q F N K E E Q H V Q Y K  5311 tacatgaacgacttcttcgacaagaacggaaagatcttcgcccaa  Y M N D F F D K N G K I F A Q  5356 ttctacgctcttcccgatggaaccattcgcttcttcgcccctcaa  F Y A L P D G T I R F F A P Q  5401 gctggtcttgaattcttctacgatggtgctcgcgtcaaattccaa  A G L E F F Y D G A R V K F Q  5446 gccgccagccaataccgtggtgccgtccgtggtatctgtggaacc  A A S Q Y R G A V R G I C G T  5491 tactccaaccaatacgccgatgacttcaccagccccaagaactgc  Y S N Q Y A D D F T S P K N C  5536 gtcatgagaaacccagaatacttcaccgccgcctacgccttcatc  V M R N P E Y F T A A Y A F I  5581 gactcctcctctcccgcccaactcaaggcccaacgcgaccaagct  D S S S P A Q L K A Q R D Q A  5626 gaacagagctcctgcgcctacaagacctacttggccggaaactac  E Q S S C A Y K T Y L A G N Y  5671 gtcagccgcaacgaaggacaaaacggaaacaagtactacaagtac  V S R N E G Q N G N K Y Y K Y  5716 aacaacaacgacaagtactacgaatccgcctacaagaactccaaa  N N N D K Y Y E S A Y K N S K  5761 tactacgatgccgccaggtacaaccaccaatacaacccatactac  Y Y D A A R Y N H Q Y N P Y Y  5806 cagaacaagaagtacgcccgcaacgaagacgcttcctactccagc  Q N K K Y A R N E D A S Y S S  5851 agcagcagctcttcctcctccagctctgacagttcctcctcttcc  S S S S S S S S S D S S S S S  5896 tcctccatggacaactcctactactacaacaacaacggtaacaac  S S M D N S Y Y Y N N N G N N  5941 aacgacaacaacaaccgcaacaacaaccgcaacaagaaccgcaac  N D N N N R N N N R N K N R N  5986 ggatcctccagcagttcctccagcagcagctcctcctccagccca  G S S S S S S S S S S S S S P  6031 agcatggagagctacgaacagagaaaccaaaacggaccatccatc  S M E S Y E Q R N Q N G P S I  6076 cacaagctttaccgctccatgaacgaaggagacaagacctgcttc  H K L Y R S M N E G D K T C F  6121 tccgtcaactccatcccaacttgcagatacccatacaagccccaa  S V N S I P T C R Y P Y K P Q  6166 ggtggagccaacaaagagattgacttctactgtgtccccagaaac  G G A N K E I D F Y C V P R N  6211 agcgaagaagctcaatacttcgagaagctcatgaagaagggagtt  S E E A Q Y F E K L M K K G V  6256 aaccccagccaactttcttccaagaaagccaacaaccaattcaag  N P S Q L S S K K A N N Q F K  6301 gtcaacatccctgaatactgtgttgcttaa 6330  V N I P E Y C V A * |  |
| --- | --- |
