# Supplementary material for: Molecular Characterization of Vitellogenin and Vitellogenin Receptor of Bemisia tabaci
Source: PLoS One. 2016 May 9;11(5):e0155306. doi: 10.1371/journal.pone.0155306 (PMC4861306; doi:10.1371/journal.pone.0155306)
Supplement: S2 File — (DOC) [file pone.0155306.s006.doc]

**Supplementary file 2:** ConSurf blast result of whitefly (*Bemisia tabaci* Asia1) vitellogenin.

**The conservation scale:**

| 1  2  3  4  5  6  7  8  9   | **Variable** | **Average** | **Conserved** | | --- | --- | --- | |
| --- | --- | --- | --- |

**e - An exposed residue according to the neural-network algorithm.**
**b - A buried residue according to the neural-network algorithm.**
**f - A predicted functional residue (highly conserved and exposed).**
**s - A predicted structural residue (highly conserved and buried).**
**X - Insufficient data - the calculation for this site was performed on less than 10% of the sequences.**

1          11         21         31         41         
**MIPVRDFSSS VIMWTPALLC LLVAAANAQY GWKNGNLYKY EINGRTLTAL**
**ebebeebbbb bbbbbbbbbb bbbbbbebee beeeeebbee ebeeebbebb**

51         61         71         81         91         
**NQVADQYAGV LFRANFYVQP FSSDRLSAYI QNAETAQVHA ELPSGYESHI**
**eebbeebbeb bbebbbebbe beeeebbbbb eebebbebbb ebeeebeebb**

101        111        121        131        141        
**PSSQLNYKSM PLSHEPFEIY LKKGVVSNLR VNKNVSDWEL NIIKAVVSQI**
**ebbebebeeb ebeeeebebb beeebbeebe beeebbebeb ebbebbbbeb**

151        161        171        181        191        
**QVDTQGQNLK KSSHNQLPKE NKPYGVYKTM EDSVTGECET LYDVSPLPEI**
**ebeeeeeebe eeeeeebeee eeeeebbeee eeebeeebee beebbebeeb**

201        211        221        231        241        
**TLQTKPWLVP FPNFRENGQF IDIVKTTNYS KCEERSAYHF GITGLTNWKP**
**ebeeeebbbe beebeeebeb bebbebeeee ebeeebbebb bbbbbeebee**

251        261        271        281        291        
**ASNQMGQFLS RSNINRVVIS GNVKYYTIQS SVSTNKIVIS PQMYESQKGM**
**bbeebbebbe ebebebbbbb bebebbbbee eebbeebbbb bebeeeeeeb**

301        311        321        331        341        
**VISVMNMTLA SFHQANGSPR SVNNYRKVNN LVYDYMAASP NAYAQHYNNN**
**bbbbbbbbbb bbeeeeeeee beeebeebee bbbbbbebee ebbbeebeee**

351        361        371        381        391        
**GASSSSSSSS SDSSSSSSSS SSSSSSSSSS SSSSSSEEEY YRNKNYNNKH**
**eeeeeeeeee eeeeeeeeee eeeeeeeeee eeeeeeeeeb beeeeeeeee**

401        411        421        431        441        
**NNNANNNDNN NKNENNHHNG DANASRNRSR RDLSQYNNGN NNNNGNNDND**
**eeeeeeeeee eeeeeeeeeb eeebbeeebe eebeeeeeee eeeeeeeeee**

451        461        471        481        491        
**AEYEKRNGHN GHNGHNGHNG HNGHNGKNVD GSSSSSSSEE NDRYNNGKFA**
**eeeeeeeeee beeeeeeeee eeeeebeeee eeeeeeeeee eeeeeeeebb**

501        511        521        531        541        
**SFARHHGSGS SSSSSSSDSS DSSSSSSSSS SSSSSSSSSS SEDNSSFGSS**
**bbbeeebeee eeeeeeeeee eeeeebbeeb ebebeebeeb eeeeeeeeee**
                                                 **f**

551        561        571        581        591        
**VSSSSEEDYE PRPSMYKAPQ TPFFPYFIGN YGNSIQSAKQ VNGVALARKL**
**eeeeeeeeee eeeeeeeeee eebbbbbbbb eeeeeeeeee eebbeebeeb**
 **f**       **f**  **f**    **ff**   **f**  **s**       **f**  **f**  **f**

601        611        621        631        641        
**AQEIAEELND PRQITQKSTL AKFNMLVEEL RTLDAKQMEQ ASQELHFNSA**
**beebbeebee eeeeeeeeeb eebebbbbbb ebbeeeebee beeebeeeee**
   **s**               **f**             **f** **s**   **f**

651        661        671        681        691        
**QASSHSRQDA LKSLAWKSFC DALVEAGTGP AFLQIQKIIE HQQVSDAEAA**
**eeeeeeeeee eeeebbebbe ebbbeeeeee bbbbbeebbe eeebeeeebb**
                    **f fs**   **fff** **f s**   **s**   **s**         **fss**

701        711        721        731        741        
**RMISRLPVTA RFPDKEYMNS FFNFVRSNNV QHQNQLNETA LLAFAELCRK**
**ebbeebeeeb eeeeeebbee bbebbeeeee eeeeebebbb bbbbbebbee**
           **f** **ff**                **f**  **f**   **f**            **f**

751        761        771        781        791        
**ADVNARNAHN YYPVHVYGRV LPEHAKAVAH QYLPYYEQNL KRAVANGDSR**
**bebeeeebee ebebebbbeb eeeeeeebbe ebbeebeeeb eebbeeeeee**
   **f**        **sf**                      **f**     **s**  **s**    **ff**

801        811        821        831        841        
**KIQAYIRAIG NFAHPKILEV FEPYLEGKVP ISNFQRTVMV LSLNELARVY**
**ebebbbbbbb bbeeeebbeb beeebeeeee beebeebbbb bbbeebbeee**
  **f**    **s** **s**  **ff**   **s**    **fffsff**     **f**  **ff**   **s**

851        861        871        881        891        
**PNLARNVLFK IYQNTQENQE VRVAAVFLIF GTNPSAQTLQ RMAQFTYEDQ**
**eeebeebbbe bbeeeeeeee bbbbbbbbbb eeeeebebbe ebbebeeeee**
**f**   **f** **s**  **f**   **f**  **f**     **s**  **s**       **f** **ff**    **f fss**  **f**  **f**

901        911        921        931        941        
**DQQVNAAVSS AIRNAAKKSA GIREELAEAA QSAVDLLNPK TYGLQFSKKW**
**eeebeebbee bbeebeeeee eeeeebbeeb eebeebbeee eeeeeebeeb**
**f**  **sff** **sff** **s** **fsf**             **fs**  **s**              **s**

951        961        971        981        991        
**LRDYIVKEEN LAYSVYADTI QGDDSLFPNQ YYAAFFRHVG GFNKRVASFR**
**beeebeeeee eebebbbebb eeeeeebeeb bebebeeeee eeeeeeeebe**
                       **f** **ff**  **f**

1001       1011       1021       1031       1041       
**AFASSASDLY DRVADSFYFA EQYQDKSFEK FSKYSAEEIF KNFNFKADYP**
**bbbeebeebb ebbeeebeee eeeeeeeeee eeeeebeebb ebbebeeeee**
   **ff**                                         **s**

1051       1061       1071       1081       1091       
**KELEAYFQYY FLGSKQYSFI NEEIFNQIPR DLESALNKAA NGYSFNNTKF**
**eebeeebbbe beeeeebbeb eeeebeebee ebeebbeeee eeeeeeebeb**
   **ff**                 **f**  **f**

1101       1111       1121       1131       1141       
**YNDFALTIGF PTATGLPFSY TIKLPTLLNF GGEVKAKVQG FKADNNKFRI**
**eeeeebebbb ebeeeebbbb ebeeeebbeb eeebeeeeee eeeeeeeeee**
           **f** **f** **f** **s**        **f**

1151       1161       1171       1181       1191       
**PEAVNVTAAI DVTYSTKLET KFGFVTPFDH QRYVAGVDKN INFNLPLKFN**
**eeebeeeeeb ebbbbeeeee ebbbbbbbee eebbbebeee bebebebebe**
                         **s**  **s**  **f**     **f**   **f**     **f**

1201       1211       1221       1231       1241       
**VNLDVYNTKA EIIVKPLNNQ HEQRVFHYSS YPYTAFYSIF DFAPVQFNKN**
**bebeeeeeeb ebebeeeeee eeeebbebbb bebbbeeebe ebeeeeeeee**
                **f**           **f** **s**   **f** **s**          **f**

1251       1261       1271       1281       1291       
**MKKIQTNNHK NEYNQAFGND KFGLNFRANY KGDYQYFDFA TFYNYFQRND**
**eeebeeeeee eeeeeeeeee eeebbbebee eeeeebeebe ebbeebeeee**
                  **f**

1301       1311       1321       1331       1341       
**LVTFFFYPWA EQEIKQNDFN FYFNPSASDN KAAKFTFNYA SKYAAKEQAD**
**bbeebbbbbe eeeeeeeebe bebeeeeeee eebebebebe eeeeeeeeee**
                  **f**          **f**

1351       1361       1371       1381       1391       
**HESRNANTND AVTSNNKPDS EERLNEFVRK SYAGINSAFV NAFDFSAQFL**
**eeeeeeeeee eeeeeeeeee eeeeeebbee beeebeebeb ebbebbbebe**
                    **f**  **f**                      **f**

1401       1411       1421       1431       1441       
**GQKEADYVCT FAFARSPVAE KSRFLFYGHY NTANNKKQQC AFHASAEMPN**
**eeeeeebbbb bbbbeeeeee ebebbbbbee eeeeeeeeeb bbebeeeeee**
         **s** **s**   **f** **ff**    **f**                **f**          **f**

1451       1461       1471       1481       1491       
**VPLTNPAAAM KAEPASKIYA NFKFGESFEN AAKVHFNANL KQSSERRQFL**
**eeebebeebb eeeeeeebeb ebeeeeeeee eeebebeeee eeeeeeeeeb**
    **f**                     **f**                  **ff**

1501       1511       1521       1531       1541       
**RNNALYKQCE SEMERGQYFL PACRNFTVAD NRMNEYYYNF NFQNIPEYFK**
**eeeeebeebe eeeeeeeeee eebeebeeeb ebeeebebeb ebeeeeeebe**
        **s**    **f**   **f**      **s**      **s**   **f**

1551       1561       1571       1581       1591       
**NYTYQAFAFA RHMGYQYQSE NVVNPHYKPN EIEGFFKFSP SFRYANFSFA**
**ebbeebbebb eebeeeebee eeeeeeeeee ebebebebee eeeebebebe**

1601       1611       1621       1631       1641       
**SPALSAAFDN VPVNPYFAAI FAPHPTYTAF DFFMQETFRS KYQAACVADK**
**beebebebee bebeebbeeb bbbeeeeeee eebeeeeeee eeeeebebee**
         **f**                                      **s**  **f**

1651       1661       1671       1681       1691       
**GFATTFDNRT FPAHFQNNWY VLMAYMNRNN YYNNNFNQYL QQNKNQHSYR**
**eebeebeeee beeebeebbb bbbbeeeeee eeeeeeeebe eeeeeeeeee**
    **fsff**                               **f**

1701       1711       1721       1731       1741       
**DYNEKRFYSA VLARDNSHGQ KELKVVLNNG EYEFNFEPAS QNAGFSNSFS**
**eeeeeeeebb bbbeeeeeee eebebbbeee eeebebeeee eebebeeeee**
                      **f**

1751       1761       1771       1781       1791       
**ASNPAAKVQF NKEEQHVQYK YMNDFFDKNG KIFAQFYALP DGTIRFFAPQ**
**eeeeeeebee eeeebeeeee ebeeeeeeee eebbebbeee eeebebebee**
           **f**

1801       1811       1821       1831       1841       
**AGLEFFYDGA RVKFQAASQY RGAVRGICGT YSNQYADDFT SPKNCVMRNP**
**eebebbbeee ebebebeeeb eeebebebbe eeeeeeeeee eeeebbeeee**
       **ff**  **f**          **f**   **fsfssf** **ff**    **ff**   **f** **f**     **f**

1851       1861       1871       1881       1891       
**EYFTAAYAFI DSSSPAQLKA QRDQAEQSSC AYKTYLAGNY VSRNEGQNGN**
**eebbbbbbbe eeebeeeeee eeeeeeeeeb eeeeeebeeb beeeeeeeee**
  **s**                       **f**              **f**   **f**

1901       1911       1921       1931       1941       
**KYYKYNNNDK YYESAYKNSK YYDAARYNHQ YNPYYQNKKY ARNEDASYSS**
**eeeeeeeeee eeebeeeeee eeeeeeeebe beeeeeeeee beeeebeebe**
                **f**

1951       1961       1971       1981       1991       
**SSSSSSSSSD SSSSSSSMDN SYYYNNNGNN NDNNNRNNNR NKNRNGSSSS**
**eeeeeeeeee eeeebebbee eeeeeeeeee eeeeeeeeee eeeeeeeeee**

2001       2011       2021       2031       2041       
**SSSSSSSSSP SMESYEQRNQ NGPSIHKLYR SMNEGDKTCF SVNSIPTCRY**
**eeeeeeeeee eeeeeeeeee eebeeeeebe beeeeeebbb eeeeeeebee**
                             **f**           **ss f**  **f**   **s**

2051       2061       2071       2081       2091       
**PYKPQGGANK EIDFYCVPRN SEEAQYFEKL MKKGVNPSQL SSKKANNQFK**
**ebeeeeeeee ebebebeeee eeebeebeee beeeeeeebe ebeeeeeeee**
         **f**     **s**              **f**   **fffff** **f**  **f**  **f**

2101       
**VNIPEYCVA**
**bebeeeeee**
   **f**  **f**
