# Supplementary material for: Molecular Characterization of Vitellogenin and Vitellogenin Receptor of Bemisia tabaci
Source: PLoS One. 2016 May 9;11(5):e0155306. doi: 10.1371/journal.pone.0155306 (PMC4861306; doi:10.1371/journal.pone.0155306)
Supplement: S3 File — Blue and red colour show vitellogenin (PS51211) and VWFD (PS51233) domains, respectively. N-terminus signal peptide is bold and underlined. (DOC) [file pone.0155306.s007.doc]

**Supplementary file 3.** Protein sequence whitefly (*Bemisia tabaci* Asia 1) vitellogenin with colour demarcation of important domains. Blue and red colour show vitellogenin (PS51211) and VWFD (PS51233) domains, respectively. N-terminus signal peptide is bold and underlined.

>Bemisia_tabaci_Vitellogenin

**MIPVRDFSSSVIMWTPALLCLLVAAANA**QYG**WKNGNLYKYEING**

**RTLTALNQVADQYAGVLFRANFYVQPFSSDRLSAYIQNAETAQVHAELPSGYESHIPS**

**SQLNYKSMPLSHEPFEIYLKKGVVSNLRVNKNVSDWELNIIKAVVSQIQVDTQGQNLK**

**KSSHNQLPKENKPYGVYKTMEDSVTGECETLYDVSPLPEITLQTKPWLVPFPNFRENG**

**QFIDIVKTTNYSKCEERSAYHFGITGLTNWKPASNQMGQFLSRSNINRVVISGNVKYY**

**TIQSSVSTNKIVISPQMYESQKGMVISVMNMTLASFHQANGSPRSVNNYRKVNNLVYD**

**YMAASPNAYAQHYNNNGASSSSSSSSSDSSSSSSSSSSSSSSSSSSSSSSSSEEEYYR**

**NKNYNNKHNNNANNNDNNNKNENNHHNGDANASRNRSRRDLSQYNNGNNNNNGNNDND**

**AEYEKRNGHNGHNGHNGHNGHNGHNGKNVDGSSSSSSSEENDRYNNGKFASFARHHGS**

**GSSSSSSSSDSSDSSSSSSSSSSSSSSSSSSSSEDNSSFGSSVSSSSEEDYEPRPSMY**

**KAPQTPFFPYFIGNYGNSIQSAKQVNGVALARKLAQEIAEELNDPRQITQKSTLAKFN**

**MLVEELRTLDAKQMEQASQELHFNSAQASSHSRQDALKSLAWKSFCDALVEAGTGPAF**

**LQIQKIIEHQQVSDAEAARMISRLPVTARFPDKEYMNSFFNFVRSNNVQHQNQLNETA**

**LLAFAELCRKADVNARNAHNYYPVHVYGRVLPEHAKAVAHQYLPYYEQNLKRAVANGD**

**SRKIQAYIRAIGNFAHPKILEVFEPYLEGKVPISNFQRTVMVLSLNELARVYPNLARN**

**VLFKIYQNTQENQEVRVAAVFLIFGTNPSAQTLQRMAQFTYEDQDQQVNAAVSSAIR**N

AAKKSAGIREELAEAAQSAVDLLNPKTYGLQFSKKWLRDYIVKEENLAYSVYADTIQG

DDSLFPNQYYAAFFRHVGGFNKRVASFRAFASSASDLYDRVADSFYFAEQYQDKSFEK

FSKYSAEEIFKNFNFKADYPKELEAYFQYYFLGSKQYSFINEEIFNQIPRDLESALNK

AANGYSFNNTKFYNDFALTIGFPTATGLPFSYTIKLPTLLNFGGEVKAKVQGFKADNN

KFRIPEAVNVTAAIDVTYSTKLETKFGFVTPFDHQRYVAGVDKNINFNLPLKFNVNLD

VYNTKAEIIVKPLNNQHEQRVFHYSSYPYTAFYSIFDFAPVQFNKNMKKIQTNNHKNE

YNQAFGNDKFGLNFRANYKGDYQYFDFATFYNYFQRNDLVTFFFYPWAEQEIKQNDFN

FYFNPSASDNKAAKFTFNYASKYAAKEQADHESRNANTNDAVTSNNKPDSEERLNEFV

RKSYAGINSAFVNAFDFSAQFLGQKEADYVCTFAFARSPVAEKSRFLFYGHYNTANNK

KQQCAFHASAEMPNVPLTNPAAAMKAEPASKIYANFKFGESFENAAKVHFNANLKQSS

ERRQFLRNNALYKQCESEMERGQYFLPACRNFTVADNRMNEYYYNFNFQNIPEYFKNY

TYQAFAFARHMGYQYQSENVVNPHYKPNEIEGFFKFSPSFRYANFSFASPALSAAFDN

VPVNPYFAAIFAPHPTYTAF**DFFMQETFRSKYQAACVADKGFATTFDNRTFPAHFQNN**

**WYVLMAYMNRNNYYNNNFNQYLQQNKNQHSYRDYNEKRFYSAVLARDNSHGQKELKVV**

**LNNGEYEFNFEPASQNAGFSNSFSASNPAAKVQFNKEEQHVQYKYMNDFFDKNGKIFA**

**QFYALPDGTIRFFAPQAGLEFFYDGARVKFQAASQYRGAVRGICGTYSNQYADDFTSP**

**KN**CVMRNPEYFTAAYAFIDSSSPAQLKAQRDQAEQSSCAYKTYLAGNYVSRNEGQNGN

KYYKYNNNDKYYESAYKNSKYYDAARYNHQYNPYYQNKKYARNEDASYSSSSSSSSSS

SDSSSSSSSMDNSYYYNNNGNNNDNNNRNNNRNKNRNGSSSSSSSSSSSSSPSMESYE

QRNQNGPSIHKLYRSMNEGDKTCFSVNSIPTCRYPYKPQGGANKEIDFYCVPRNSEEA

QYFEKLMKKGVNPSQLSSKKANNQFKVNIPEYCVA
