# Supplementary material for: Molecular Characterization of Vitellogenin and Vitellogenin Receptor of Bemisia tabaci
Source: PLoS One. 2016 May 9;11(5):e0155306. doi: 10.1371/journal.pone.0155306 (PMC4861306; doi:10.1371/journal.pone.0155306)
Supplement: S4 File — LPD_N, DUF1943, VWFD domains are underlined by blue, green and red colours, respectively. (PDF) [file pone.0155306.s008.pdf]

Supplementary file 4. Multiple sequence alignment of full length vitellogenin protein sequences of selected insects from each order. LPD_N, DUF1943, VWFD domains are underlined by blue, green and red colours, respectively.

1	2	3	4	5	6	7	8	9
Variable				Average				Conserved

```
A7BK94_Nilaparvata_lugens           ------------------------------MNGLTL-ILCVIAVAGVS---
Q868N5_Apis_mellifera               -------------------------------MLLLL-TLLLFAGTVAA---
R4KZ43_Helicoverpa_armigera         -------------------------------MKLLV-LAAFIAVVSSG---
Q9U5D8_Plautia_stali                ------------------------------MLWSSA-LLLAFACLAAA---
O02024_Riptortus_clavatus           -------------------------------MWSPV-IICLLVGLASA---
D6W7J2_Tribolium_castaneum          -------------------------------MWSKI-VLCFFVGLACA---
D6W721_Tribolium_castaneum          -------------------------------MWSQI-LLCLLVGLAFA---
B_tabaci_Asia1                      -------------------MIPVRDFSSSVIMWTPA-LLCLLVAAANA---
BAG84131_Tetranychus_urticae        ---------------------------------------------------
ADU04394.1_B_tabaci_Q               -------------------------------MWTPV-LLCLLVAAANA---
Q16927_Aedes_aegypti                MATDGITSRFGFNERRRRTHNRNSCRILEDKMLAKL-LLLALAGLTAAYQY
G0STK0_Nilaparvata_lugens           ------------------------------MNGLTL-LLCAIAVAGVS---
Q9BPS0_Periplaneta_americana        ------------------------------MMWKTL-LCCLLAVSAAA---
E0VZ52_Pediculus_humanus_corporis   -------------------------------MWTPFALFVILAAVASA---
B0W351_Culex_quinquefasciatus       ------------------------------MMWWKF-VLIVLVGTCFAEQQ
O76823_Blattella_germanica          ------------------------------MTWNAL-LCCLLVSAASA---
Q7PQM2_Anopheles_gambiae            -------------------------------MIAKL-LLLTLVGLCTAYQY
UPI000258E3A9_Megachile_rotundata   -------------------------------MWLSL-TLLVLAGVVSAKS-
ADU04392.1_1_B_tabaci_B             -------------------------------MWTPV-LLCLLVAAANA---
Q9U8M0_Periplaneta_americana        -------------------------------MWKGF-LCCLLVAGVTS---
ADU04393.1_B_tabaci_SHJ-II          -------------------------------MWTPA-LLCLLVAAANA---
Q27309_Bombyx_mori                  -------------------------------MKLFV-LAAIIAAVSSD---
```

```
A7BK94_Nilaparvata_lugens           ------------AS-----------GS------------------------
Q868N5_Apis_mellifera               ------------DFQHN----------------------------------
R4KZ43_Helicoverpa_armigera         ------------KLSEQQQD-----NQ------------------------
Q9U5D8_Plautia_stali                ------------DY-----------G-------------------------
O02024_Riptortus_clavatus           ------------DNP------------------------------------
D6W7J2_Tribolium_castaneum          ------------SHNP---------G-------------------------
D6W721_Tribolium_castaneum          ------------SENP---------G-------------------------
B_tabaci_Asia1                      ------------QY-----------G-------------------------
BAG84131_Tetranychus_urticae        ---------------------------------------------------
ADU04394.1_B_tabaci_Q               ------------QY-----------G-------------------------
Q16927_Aedes_aegypti                ENSFKGYNPGYKGYDAGYKGYGYDAGYKYNNQGYSYKNGFEYGYQNAYQA
G0STK0_Nilaparvata_lugens           ------------AS-----------GS------------------------
Q9BPS0_Periplaneta_americana        ------------LD-----------G-------------------------
E0VZ52_Pediculus_humanus_corporis   ------------DY-----------G-------------------------
B0W351_Culex_quinquefasciatus       QFKRPQ------SYDEAAQRYEKLMDGGAKHYLHDNLA-------------
O76823_Blattella_germanica          ------------ITP----------G-------------------------
Q7PQM2_Anopheles_gambiae            SY----------EYEFPSSRPFNKTGF------------------------
UPI000258E3A9_Megachile_rotundata   ------------NY-----------G-------------------------
ADU04392.1_1_B_tabaci_B             ------------QY-----------G-------------------------
Q9U8M0_Periplaneta_americana        ------------LQE------------------------------------
ADU04393.1_B_tabaci_SHJ-II          ------------QY-----------G-------------------------
Q27309_Bombyx_mori                  ------------RFSSQSQSTGGQTYP------------------------
```

LPD_N

```
A7BK94_Nilaparvata_lugens           --------------GPWNS-NQQYRYHVQGRSLSAMHQSGSNQYVGMHLR
Q868N5_Apis_mellifera               ----------------WQV-GNEYTYLVRSRTLTSLGDL-SDVHTGILIK
R4KZ43_Helicoverpa_armigera         --------------WPWQS-GKLYRYEVNSHTLARLH---EGASSGNAFK
Q9U5D8_Plautia_stali                ----------------WNT-GYQYVYNFEARTVAGIHQV-ADQYSGNYIR
O02024_Riptortus_clavatus           ----------------WKA-GTKYTYQIQGRTLTGLHQV-ADQYTGVMIQ
D6W7J2_Tribolium_castaneum          ----------------WQH-NHEYVYQIRGRSLTGLDQI-SSNHAGMLFK
D6W721_Tribolium_castaneum          ----------------WNN-NYHYVYEVRGRSLAGLNQV-SNEYSGIIFK
B_tabaci_Asia1                      ----------------WKN-GNLYKYEINGRTLTALNQV-ADQYAGVLFR
BAG84131_Tetranychus_urticae        --------------------------------------------------
ADU04394.1_B_tabaci_Q               ----------------WKN-GNLYKYEINGRTLTALNQV-ADQYAGVLFR
Q16927_Aedes_aegypti                AFYKHRPNVTEFEFSSWMP-NYEYVYNVTSKTMTALAEL-DDQWTGVFTR
G0STK0_Nilaparvata_lugens           --------------GPWNS-NQQYRYHVQGRSLSAMHQSGSNQYVGMHLR
Q9BPS0_Periplaneta_americana        ----------------WEP-GKRYEYHVRGRTLTALHEV-ANQYSGFRFK
E0VZ52_Pediculus_humanus_corporis   ----------------FKP-GKEYHYEVTSTTLTSLHQV-ADRYAGVSMR
B0W351_Culex_quinquefasciatus       ----------------WEP-NQVYWYNVTTKTMTALPDV-TEQWTGVLTR
O76823_Blattella_germanica          ----------------WLPINSQLDYHVHGRTFSSLFQV-ANQYTGILYK
Q7PQM2_Anopheles_gambiae            ------------EFGAWEP-NKEYVYNVTTKTMTALPDL-EDYWTGIVTH
UPI000258E3A9_Megachile_rotundata   ----------------WKV-GNEYSYVVQSRTFTNLAEF-PKDYAGILMK
ADU04392.1_1_B_tabaci_B             ----------------WKN-GNLYKYEVNGRTLTALNQV-ADQYAGVLFR
Q9U8M0_Periplaneta_americana        ----------------WDP-NRQYVYKVESRAFTALHQI-SNQYAGILMR
ADU04393.1_B_tabaci_SHJ-II          ----------------WKN-GNLYKYEINGRTLTALNQV-ADQYAGVLFR
```

```
Q27309_Bombyx_mori                    ---------------SPWQV-GKQYRYEVTSRTLAHLQ---EGPSSGSAFK
```

LPD_N

```
A7BK94_Nilaparvata_lugens             AELEVEAKNENQAVFKISKAEYADVHQNLSGGWQQELRSNELQYKQLPLS
Q868N5_Apis_mellifera                 ALLTVQAKDSNVLAAKVWNGQYARVQQSMPDGWETEISDQMLELRDLPIS
R4KE43_Helicoverpa_armigera           AQFIVRVKYPGRLLAKLENPVHAQFNQQLPNNMAVP---SDLKYETVQNL
Q9U5D8_Plautia_stali                  GKLYAYVEPDNKVTFQFQDAKYADFNEHFPEGWATDLHHYQFSYKSFPIK
O02024_Riptortus_clavatus             GQLTVRAESDNKATAVIENAKYADLQANLSRGWDSEIQENELQYNELPIS
D6W7J2_Tribolium_castaneum            AKLWVRPRSDGHLGARIIEPQYAEIHSKLYRSWDQEIPDSELSYSPISLS
D6W721_Tribolium_castaneum            AQLYIQPRSDGNLGAKISNAQYAKIHEELSEGWETEIPDSQVSYQQLPLS
B_tabaci_Asia1                        ANFYVQPFSSDRLSAYIQNAETAQVHAELPSGYESHIPSSQLNYKSMPLS
BAG84131_Tetranychus_urticae          --------------------------------------------------
ADU04394.1_B_tabaci_Q                 ANFYVQPFSSDRLSAYIQNAETAQVHAELPSGYESHIPSSQLNYKSMPLS
Q16927_Aedes_aegypti                  AYLVIRPKSRDYVVAYVKQPEYAVFNERLPYGYATKFYHDMFKFQPMPMS
G0ZTK0_Nilaparvata_lugens             AELEVEAKNENQAVFKISKAEYADVHQNLSGGWQQELRSNELQYKQLPLS
Q9BPS0_Periplaneta_americana          GKLVIEPHTPSVLRGQLKDTYHMTVHRMLPDGWDQKFEERESNWERVGMK
E0VZ52_Pediculus_humanus_corporis     NKLTLTPVKEDEYLGTFTQSEVTEVHTTLPGGVDHSVPLENAKYSPISLP
B0W351_Culex_quinquefasciatus         AKMVINPKSDGYVVGRIDRAHYAQFNQYLADGHRSELSDLKLTWKPMPLS
O76823_Blattella_germanica            ARLSLDRNEDQLITGKVTEAQFSPVNTQFSSGWDESVPDEKLHWDVVPMS
Q7PQM2_Anopheles_gambiae              GYLVIRPKDHNYVVAYIDRPMYAAFNEYLPRGYRTELSRFNLKWQPMPFS
UPI000258E3A9_Megachile_rotundata     GYLTVQAKEDGKLTAKLTRGQYFHIHSNMSRGWD----------------
ADU04392.1_1_B_tabaci_B               ANFIVQPLSSDRLSAQIQNAEIAQVHTELPAGYESHIPSSQLNYKNMPLS
Q9U8M0_Periplaneta_americana          AKLIIQPKTREELHAQLVEVEHSQINKELPSGWDQEIEIHD--WQQLSAL
ADU04393.1_B_tabaci_ZHJ-II            ANFYVQPFSSDRLSAYIQNAETAQVHAELPSGYESHIPSSQLNYKSMPLS
Q27309_Bombyx_mori                    AQFTIRVKSPGRLQAKLENPQHGNFNEQLPDPRELPV---DLKYQPTPNI
```

LPD_N

```
A7BK94_Nilaparvata_lugens             QANQVFQVNYK-QGAVRSLQVNRNTPTWELNMIKGFVSLFQVDVTGQNAI
Q868N5_Apis_mellifera                 G--KPFQIRMK-HGLIRDLIVDRDVPTWEVNILKSIVGQLQVDTQGENAV
R4KE43_Helicoverpa_armigera           D--KPFEISVE-GGRVVALSLPSAFLLSHENLLKGLLSTLQVDLSAYHHV
Q9U5D8_Plautia_stali                  N--DVFQVYFN-NGLVDKLFVYKDIPHWELNFIKAIASQFQTDVQGQNLK
O02024_Riptortus_clavatus             K--DPFEIHFR-NGVIEKMVVNKDIPQWQLNFYKSIASQFQVDTQGKNLK
D6W7J2_Tribolium_castaneum            S--KPFLIKMD-NNAVSDLVVDRSISNWEANMIKAIVSQLQLDTNAENLI
D6W721_Tribolium_castaneum            S--KPFQIGLY-KGVIRNVMVDKQIKNWEANMIKSIVSQLQLDTKATNLI
B_tabaci_Asia1                        H--EPFEIYLK-KGVVSNLRVNKNVSDWELNIIKAVVSQIQVDTQGQNLK
BAG84131_Tetranychus_urticae          --------------------------------------------------
ADU04394.1_B_tabaci_Q                 H--EPFEIYLK-KGVVSNLRVNKNVSDWELNIIKAVVSQIQVDTQGQNLK
Q16927_Aedes_aegypti                  S--KPFGIRYH-KGAIKGLYVEKTIPNNEVNILKAWISQLQVDTRGANLM
G0ZTK0_Nilaparvata_lugens             QANQVFQVNYK-QGAVRSLQVNRNTPTWELNMIKGFVSLFQVDVTGQNAI
Q9BPS0_Periplaneta_americana          N--KPFEVHVGNEFQFNKLIVTEDTPVWETNMIKGVLSQIQVNLKEV---
E0VZ52_Pediculus_humanus_corporis     R--EPFVVKTK-DGIVDELVVSKDLPVWHVNVIRSIVSQFQIDTQGRRLI
B0W351_Culex_quinquefasciatus         S--KPFGIKYK-KGAIKGLYVEKTVPNHEVNILKSWVSQLQLDLFGANLI
O76823_Blattella_germanica            Q--QPFQIELNSRGEVRKLRVNKFVELWEINMIKAIISQLQVVVD-----
Q7PQM2_Anopheles_gambiae              S--KPFGIYYN-KGAVKGFYVEKTVPNHEVNMLKGWVSQLQLDTQGAYVI
UPI000258E3A9_Megachile_rotundata     -------------ESILDIVIVEEDVPNWEVVLLKSIISQFQVDIKGKKLV
ADU04392.1_1_B_tabaci_B               E--QPFEINLK-QGVVSNLRVNKNVSDWELNIIKAVVSQIQVDTQGQNLK
Q9U8M0_Periplaneta_americana          K--DAFAIILQ-NGHIVNVKVKDSTPNWAVNILKGILSTLQVNTQADNLV
ADU04393.1_B_tabaci_ZHJ-II            H--EPFEIYLK-KGVVSNLRVNKNVSDWELNIIKAVVSQIQVDTQGQNLK
Q27309_Bombyx_mori                    D--KVFEIEID-GGRIVSLDLPTSVPVPQENLIKGLISALQLDTSAHRVI
```

LPD_N

```
A7BK94_Nilaparvata_lugens             KSRRNIVPNGQ--QVSGSFKVMLDSVTGKCLTHYDVDELPMRVVQQHPEI
Q868N5_Apis_mellifera                 KVNSVQVPTDD--EPYASFKAMLDSVGGKCLVLYDIAPLSDFVIHRSPEL
R4KE43_Helicoverpa_armigera           HNHQDSYDKE---RQQGLFKKMLTDVTGDCLTLYTVSPV------AAEWR
Q9U5D8_Plautia_stali                  KSKFNDVPKGN--QLTGIFKTWLDSVNGEYLVMNEVSVLPEYLIESQPEL
O02024_Riptortus_clavatus             DSKINLVPSGQSGEPMGVYKTMLDSVNGIYLTLYDISVLPEYILQARPEL
D6W7J2_Tribolium_castaneum            PSSINILTQEG--SSTAVFKTMLPTVTGESLTLYEIHPLPDYILQTTPWL
D6W721_Tribolium_castaneum            PSSINILTQED--SNTAVFKTMLETVTGVCLTLYEIHPYPEYVLQSKPWV
B_tabaci_Asia1                        KSSHNQLPKEN--KPYGVYKTMLDSVTGECLTLYDVSPLPEITLQTKPWL
BAG84131_Tetranychus_urticae          --SASPMPKDQ------TCTLMKDSI------------------------
ADU04394.1_B_tabaci_Q                 KSSHNQLPKEN--KPYGVYKTMLDSVTGECLTLYDVSPLPEITLQTKPWL
Q16927_Aedes_aegypti                  HSSKPIHPSKN--EWNGHYKVMLPLVTGECLTHYDVNLIPAYMIQAHKQW
G0ZTK0_Nilaparvata_lugens             KSRRNIVPNGQ--QVSGSFKVMLDSVTGKCLTHYDVDELPMRVVQQHPEI
Q9BPS0_Periplaneta_americana          -GPDPRDEQED--RLRKIFKVHLSSVTGRCLVLYDITPITKFNMLPQ---
E0VZ52_Pediculus_humanus_corporis     PSKLNSVPHGD--ETTAVYKVMLDTVEGECLTLYDVTPLPKYVVLTTPSL
B0W351_Culex_quinquefasciatus         KSKYNQLPENE--TANAVYKTMLPSVSGECLTLYDVNVLPKYKIQSHDEW
O76823_Blattella_germanica            -------------EDKKVYRVFLSTVTGRCLALYEVDHLYPTTYLNPWQW
Q7PQM2_Anopheles_gambiae              KSEFNQFPENN--TLTGVYKTMLPSVTGECLTLYDVNPVPEFHFQSHKEW
UPI000258E3A9_Megachile_rotundata     EGHDVKVPDEN--DPRGGFTVLLDYFAGLNLVRYDFKPLSEQQLRTKPEL
ADU04392.1_1_B_tabaci_B               KSSHNQLPKEN--KPYGVYKTMLDSVTGECLTLYDVSPLPEITLQTRPWL
Q9U8M0_Periplaneta_americana          RHRYNILPNAS--TDSAVYSIRLTTITGECLVEYDVSPLPALPLLQHPEL
```

```
ADU04393.1_B_tabaci_ZHJ-II           KSSHNQLPKEN--KPYGVYKTMEDSVTGECETLYDVSPLPEITLQTKPWL
Q27309_Bombyx_mori                   HDSQNNYDRE---QQQGLFRKMETDVTGDCETLYTVSPV------ASEWR
```

LPD_N

```
A7BK94_Nilaparvata_lugens            APLA-VK-QQGQGQGQSHSRLIQVVKSRNFSNCDNPVTYHFGFTQESNFE
Q868N5_Apis_mellifera                VPMPTLK-GDGR--------HMEVIKIKNFDNCDQRINYHFGMTDNSRLE
R4KZ43_Helicoverpa_armigera          RELPRFANEEDP---------MEITKSKNYGHCHHRVAYHFGVPEGAEWT
Q9U5D8_Plautia_stali                 APLPHLR-NEGE--------FYELLKTRNFSSSNRHLVYRYGFNGHEGWD
O02024_Riptortus_clavatus            APLPHLK-GDGE--------FIEVVKTRNFSNSNQHVIYRFGSSGHEGWE
D6W7J2_Tribolium_castaneum           APLKHLK-GRGQ--------VIEVVKNKNFTHSKETPSYHFGFHGVDRFD
D6W721_Tribolium_castaneum           VPHKHLI-EDGD--------VIEIVKNKNFTHSEQSPSYHAGLDGIYGYQ
B_tabaci_Asia1                       VPFPNFR-ENGQ--------FIDIVKTTNYSKCEERSAYHFGITGLTNWK
BAG84131_Tetranychus_urticae         ---------------------------RTFDNVTYRT-------------
ADU04394.1_B_tabaci_Q                VPFPNFR-ENGQ--------FIDIVKTTNYSKCEERSAYHFGITGLTNWK
Q16927_Aedes_aegypti                 VPQGQLRGEDGQ--------FIQVTKTQNFDRCDQRMGYHFGFTGYSDFR
G0ZTK0_Nilaparvata_lugens            APLAVKQQGQGQ--GQCHSRLIQVVKSRNFSNCDNPVTYHFGFTQESNFE
Q9BPS0_Periplaneta_americana         -PLVEIEEENVN--------VLQVMKTQNFTDCKKLPSYVHGFYNFHNVF
E0VZ52_Pediculus_humanus_corporis    APTYHVG-DKED--------LIDIVKTANFSNCEQRPGYHFGLTGLTDWK
B0W351_Culex_quinquefasciatus        VPQPQYMQQDDE--------IFEIVKAKNFSNCDRRMGYHFGITGASDIK
O76823_Blattella_germanica           TQQHDTK--------------LRIMKTHQFTNCRHNSAYKLHFNAFEYFH
Q7PQM2_Anopheles_gambiae             VPQPQWLEEDQH--------VFHVVKSRNFDHCEQRMGFHFGFSGFSDFK
UPI000258E3A9_Megachile_rotundata    VPIPELK-GSGQ--------HIDIVKTKSYDRYPEMSENVFGMTRQMKKE
ADU04392.1_1_B_tabaci_B              VPFPKLR-ENGQ--------VIDIVKATNYSKCEERSAYHFGITGLTNWK
Q9U8M0_Periplaneta_americana         APLSNVN-DN----------VIDIEKTQNFSNCKRRPAVHYGLAGIPDLE
ADU04393.1_B_tabaci_ZHJ-II           VPFPNFR-ENGQ--------FIDIVKTTNYSKCEERSAYHFGITGLTNWK
Q27309_Bombyx_mori                   RELPKFANEQDP---------VEVTKSTNYGHCHHRVAYHFGVPVGAEWT
```

LPD_N

```
A7BK94_Nilaparvata_lugens            PASNQMG--NLVSRAAMGRIIIAGE--PDSYTIHSSVTQNEIAISPFGYN
Q868N5_Apis_mellifera                PGTNKNG--KFFSRSSTSRIVISES--LKHFTIQSSVTTSKMMVSPRLYD
R4KZ43_Helicoverpa_armigera          GTAHRSEEKQFIAQSTVSRILAGKQ----GPIYKAETTSTVSVHPHLYG
Q9U5D8_Plautia_stali                 AGTNQMG--NFLSRTSQSRIILSGK--LNRFTIQSSVTTEKVLIAPHIYN
O02024_Riptortus_clavatus            AGTNQMG--DFLSRSSQSRVILSGK--LNRYTIQSAVTTEKVLVAPHVYN
D6W7J2_Tribolium_castaneum           -IDNQIG--EFFSRTSVSRAVITGN--LDSFTLQSSVTVDEVTVNPSMQD
D6W721_Tribolium_castaneum           PG-NKVG--KFLSRTSVSQAVITGT--LDKYTVQSSVTVEQIVIRPTLAD
B_tabaci_Asia1                       PASNQMG--QFLSRSNINRVVISGN--VKYYTIQSSVSTNKIVISPQMYE
BAG84131_Tetranychus_urticae         --------------------------------------------------
ADU04394.1_B_tabaci_Q                PASNQMG--QFLSRSNINRVVISGN--VKYYTIQSSVSTNKIVISPQMYE
Q16927_Aedes_aegypti                 PNTNQMG--NVASKSLVSYMYLTGN--WYNFTIQSSSMINKVAIAPSLVN
G0ZTK0_Nilaparvata_lugens            PASNQMG--NLVSRAAMGRIIIAKE--PDSYTIHSSVTQNEIAISPFGYN
Q9BPS0_Periplaneta_americana         PAQNKAG---FMSRSQQTRTIVSRNKETGRFTIRSSVTFHEVVLKPELFN
E0VZ52_Pediculus_humanus_corporis    PTTNKLG--NFLSRSSVTRVVLSGN--LKTYTVHSSVTTSKVIVSPEVYN
B0W351_Culex_quinquefasciatus        PNTNQMG--NILSKSAVSQIFVVGK--IHNHTIQSASTTSKILVSPSLVH
O76823_Blattella_germanica           LKQHKPE--TFLSNSAVSRVIADGD-NLKNFTFYSGETIHKIVLNPEIYN
Q7PQM2_Anopheles_gambiae             PNTNQMG--NIMTKSEVTQMYLTGN--WYNYTIQSVSTVNKVVVSPSLVN
UPI000258E3A9_Megachile_rotundata    PKTNK----NLMSKSSTSRIVISGS--LKDFVIQSAVSTSKTTITPSFND
ADU04392.1_1_B_tabaci_B              PASNQMG--QFLSRSNINRVIISGN--VKQYHIQSSVSTNKIVISPQMYE
Q9U8M0_Periplaneta_americana         PGQNQMG--DFLARSSVSRVVISGT--LNKFTVQSSVTTNQVVMSPEMYN
ADU04393.1_B_tabaci_ZHJ-II           PASNQMG--QFLSRSNINRVVISGN--VKYYTIQSSVSTNKIVISPQMYE
Q27309_Bombyx_mori                   GTAHKTQEQQLIGRATYSRILTGKE----GPIYKAETTSTVHVHPHLYG
```

LPD_N

```
A7BK94_Nilaparvata_lugens            QQKGVVGTLMNATLVSVSHASSGSPQ------------------------
Q868N5_Apis_mellifera                RQNGLVLSRMNLTLAKMEKTSKPLPM------------------------
R4KZ43_Helicoverpa_armigera          KQKAQVHSYVQLILVSVEQDSGVEWQ------------------------
Q9U5D8_Plautia_stali                 SQKGMVGSRVNLTLDDYSNFKGSVPT------------------------
O02024_Riptortus_clavatus            NQKGIVVSSMNITLQQA-TGSQGSPQ------------------------
D6W7J2_Tribolium_castaneum           KQRGSTRSIVNITLVSMDEQNQEFEQ------------------------
D6W721_Tribolium_castaneum           QKKGSVNSKLNVTLLQVAQPVSENEY------------------------
B_tabaci_Asia1                       SQKGMVISVMNMTLASF-HQANGSPR------------------------
BAG84131_Tetranychus_urticae         --------------------------------------------------
ADU04394.1_B_tabaci_Q                SQKGMVISVMNMTLASF-HQANGSPRNGHNGHNGNNGHNGNNGHNGHNGN
Q16927_Aedes_aegypti                 KEPALVYAQVNMTLNDV-HPYDKVPM------------------------
G0ZTK0_Nilaparvata_lugens            QQKGVVGTLMNATLVSVSHASSGSPQ------------------------
Q9BPS0_Periplaneta_americana         SQQGISVSRMNVTLEEI-KSQQHIPP------------------------
E0VZ52_Pediculus_humanus_corporis    NQKGEVISRVNVTLVSVGTGTSTGSG------------------------
B0W351_Culex_quinquefasciatus        TEKAMVLAQVNVTLNQI-KRSQKEPV------------------------
O76823_Blattella_germanica           KQKGMLVSHINVTVERKGRELTVIDY------------------------
Q7PQM2_Anopheles_gambiae             SQKAMVYAQVNMTLNEI-TPYDKYPE------------------------
UPI000258E3A9_Megachile_rotundata    AQNSTVLSKLSLTLEDMHEIKTPMPS------------------------
ADU04392.1_1_B_tabaci_B              SQKGMVVSVMNL?LASF-HQSNGSPR------------------------
```

```
Q9U8M0_Periplaneta_americana        SQKGLIVSRVNVTIKDI-EEARPIPL------------------------
ADU04393.1_B_tabaci_SHJ-II          SQKGMVISVMNMTLASF-HQANGSPR------------------------
Q27309_Bombyx_mori                  KQKAEVYSHVEMELISVDQDSGAEWP------------------------
```

LPD_N

```
A7BK94_Nilaparvata_lugens           ------------------SVQNPQKINDLVYEFNPASNSESNQRSSHYT
Q868N5_Apis_mellifera               -------------------VDNPESTGNLVYIYNNPF------------
R4KS43_Helicoverpa_armigera         ------------------KPEGTRQIKTLLYAMTTKQ------------
Q9U5D8_Plautia_stali                -------------------ISNPKTVNNLVYEYEYGK------------
O02024_Riptortus_clavatus           ------------------SVSNPREVKSLVYEYESQG------------
D6W7J2_Tribolium_castaneum          -------------------VSNPVNVG-LVYHYDNPF------------
D6W721_Tribolium_castaneum          ------------------DVTSPVDVG-VVYAYDSPY------------
B_tabaci_Asia1                      ------------------SVNNYRKVNNLVYDYMAAS---PNAYAQHYN
BAG84131_Tetranychus_urticae        -------------------------------------------------
ADU04394.1_B_tabaci_Q               NGQNNSNGSSSSSSSSSSSSGSSSSEENNNNNDNEDEKNNSGAYAQHYN
Q16927_Aedes_aegypti                -----------------GPAEDLKVFVDLVYSYNMPSDKKNYVRP----
G0STK0_Nilaparvata_lugens           ------------------SVQNPQKINDLVYEFNPASNSESNQRSSHYT
Q9BPS0_Periplaneta_americana        -------------------PRPPKDVGDLVYRYSAET-GEPSQRDSAYA
E0VZ52_Pediculus_humanus_corporis   -------------------PANPQTLKKLVYDFNGPF------------
B0W351_Culex_quinquefasciatus       -----------------GPAEDRAVFVGLVYSYNLPEDKNNWARPENET
O76823_Blattella_germanica          --------------------ELRNVGDLSYSTSLVK---------AHSM
Q7PQM2_Anopheles_gambiae            -----------------GPADDRQVFVDLVYSYNMAHDKNNFVRPANET
UPI000258E3A9_Megachile_rotundata   -------------------PTKPKSTGYRI-------------------
ADU04392.1_1_B_tabaci_B             ------------------MVSNARKINNLVYDYNAAS---PNAYAQHYN
Q9U8M0_Periplaneta_americana        -------------------PGNLQDTGDLLYSYNNAHDIEPLQRDRQ--
ADU04393.1_B_tabaci_SHJ-II          ------------------SVNNYRKVNNLVYDYMAAS---PNAYAQHYN
Q27309_Bombyx_mori                  ------------------RAGAMRPAQSILYSLSTKQ------------
```

LPD_N

```
A7BK94_Nilaparvata_lugens           RQQADNEDDSSSSSSSSSDSSSSSSSSSS----SSSSSSSSSEENNKNSKK
Q868N5_Apis_mellifera               --------------------------------------SDVEERRVSKTA
R4KS43_Helicoverpa_armigera         --------------------------MAIHDQSSSQESSESHEHIHVEVE
Q9U5D8_Plautia_stali                ----------PQNGESSEESSSSSSSSSSSSSSSSESSSSSDESNWNQSL
O02024_Riptortus_clavatus           ---------NSENLQSSQQSSQQSSSEESSSSSSSSSSSSSEENMNSMKT
D6W7J2_Tribolium_castaneum          ---------------------------------------DQTNEPHLRSQ
D6W721_Tribolium_castaneum          ----------------STDNSPRPAKQYDHGVAPGSSESQYDTNQYYGRT
B_tabaci_Asia1                      NNGASS---SSSSSSSDSSSSSSSSSSSSSSSSSSSSSSSSSSEEE-YYRNK
BAG84131_Tetranychus_urticae        --------------------------------------------------
ADU04394.1_B_tabaci_Q               NNGASSSSSSSSSSSSDSSSSSSSSSSSSSSSSSSSSSSSSSEEDQYYRRH
Q16927_Aedes_aegypti                ------GNETSSSSSSSSSSSSSSSESSSSSSESVENPKISPVEQYKPLL
G0STK0_Nilaparvata_lugens           RQQADNEDDSSSSSSSSDSSSSSSSSSSSSSSSSSSSEENNKNSKKNNKN
Q9BPS0_Periplaneta_americana        LESNSDSSSSSSSSSSEENAANSRHRSSSSSSSSRSSEEMRDSKKHPRAS
E0VZ52_Pediculus_humanus_corporis   ------------------------------------STDAQVREKWDQRQ
B0W351_Culex_quinquefasciatus       SSSSSSSSSSSSSSSSDSSSSSDSSSSSSSSSSSSSESSSSSSSSSSEEK
O76823_Blattella_germanica          RNSASMDLSSSSMSSSSSSSSSSSSSSSSSSSSSSSEEHHSHNQKLSKKR
Q7PQM2_Anopheles_gambiae            DDSSSS--SSSSSSDSDSDSSSSSDSSSSSSEEEAENFKISPAEQYKKQA
UPI000258E3A9_Megachile_rotundata   --------------------------------------------------
ADU04392.1_1_B_tabaci_B             NNGASSSSSSSSSSSSDSSSSSSSSSSSSSSSSSSSSSSSSSEEDQYYRRH
Q9U8M0_Periplaneta_americana        --------DSDFTLESDSSSSSSSSSSDENSWSRDSHSRISEENKKAQIK
ADU04393.1_B_tabaci_SHJ-II          NNGASSSSPSSSSSSSDSSSSSSSSSSSSSSSSSSSSSSSSSEEDQYYRRH
Q27309_Bombyx_mori                  -------------------------MTKHYESSSSSSSSESHEFNFPEQH
```

LPD_N

```
A7BK94_Nilaparvata_lugens           NNNNKNWNNKNQKKKNNNNRNNHNDNDNNQDNSNE---------------
Q868N5_Apis_mellifera               MNSNQIVS------------------------------------------
R4KS43_Helicoverpa_armigera         QPK-----------------------------------------------
Q9U5D8_Plautia_stali                RKPASATSTGSLSSSSSSVSSSSS--------------------------
O02024_Riptortus_clavatus           NQQMKEDRAK----------------------------------------
D6W7J2_Tribolium_castaneum          IRSGRKNSD-----------------------------------------
D6W721_Tribolium_castaneum          KRAITRPN------------------------------------------
B_tabaci_Asia1                      NYNNKHNNNANNNDNNN---------------------------------
BAG84131_Tetranychus_urticae        --------------------------------------------------
ADU04394.1_B_tabaci_Q               NNNNRNNNNNNNNNNNRNNNHNNNNNNANDNNNSANNNNNNNS-------
Q16927_Aedes_aegypti                DKVEKRGN------------------------------------------
G0STK0_Nilaparvata_lugens           WNNNKNWNNKNQKNNNNNNRNNHNYNDNNQDNSNE---------------
Q9BPS0_Periplaneta_americana        TTESQPRN------------------------------------------
E0VZ52_Pediculus_humanus_corporis   EQTL----------------------------------------------
B0W351_Culex_quinquefasciatus       DNKKISPAE-----------------------------------------
O76823_Blattella_germanica          QVPLPRPLF-----------------------------------------
Q7PQM2_Anopheles_gambiae            KEVERRGN------------------------------------------
UPI000258E3A9_Megachile_rotundata   --------------------------------------------------
```

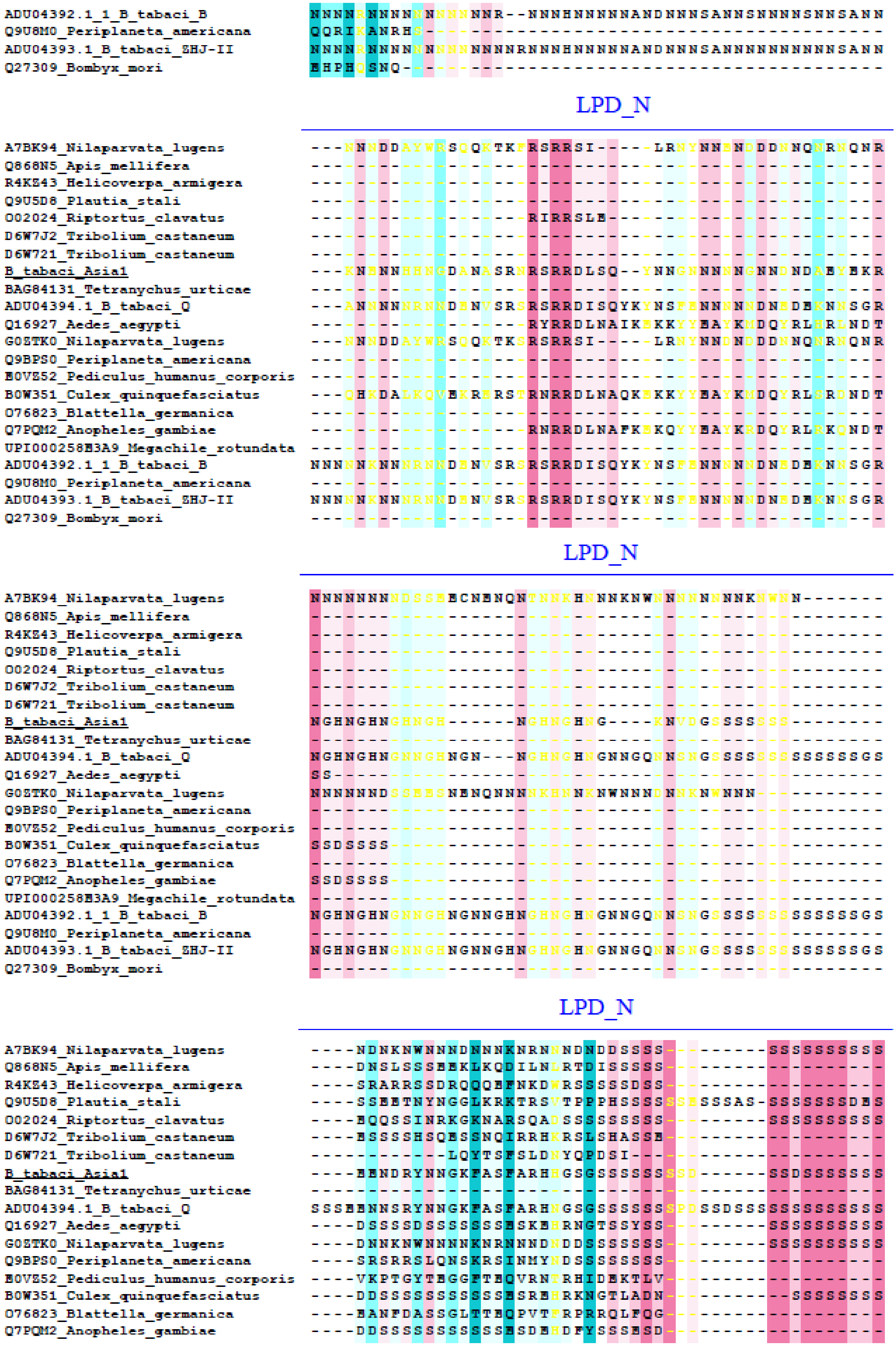
LPD_N
A7BK94_Nilaparvata_lugens
Q868N5_Apis_mellifera
R4KE43_Helicoverpa_armigera
Q9U5D8_Plautia_stali
O02024_Riptortus_clavatus
D6W7J2_Tribolium_castaneum
D6W721_Tribolium_castaneum
B_tabaci_Asia1
BAG84131_Tetranychus_urticae
ADU04394.1_B_tabaci_Q
Q16927_Aedes_aegypti
G0ZTK0_Nilaparvata_lugens
Q9BPS0_Periplaneta_americana
E0VZ52_Pediculus_humanus_corporis
B0W351_Culex_quinquefasciatus
O76823_Blattella_germanica
Q7PQM2_Anopheles_gambiae
UPI000258E3A9_Megachile_rotundata
ADU04392.1_1_B_tabaci_B
Q9U8M0_Periplaneta_americana
ADU04393.1_B_tabaci_ZHJ-II
Q27309_Bombyx_mori

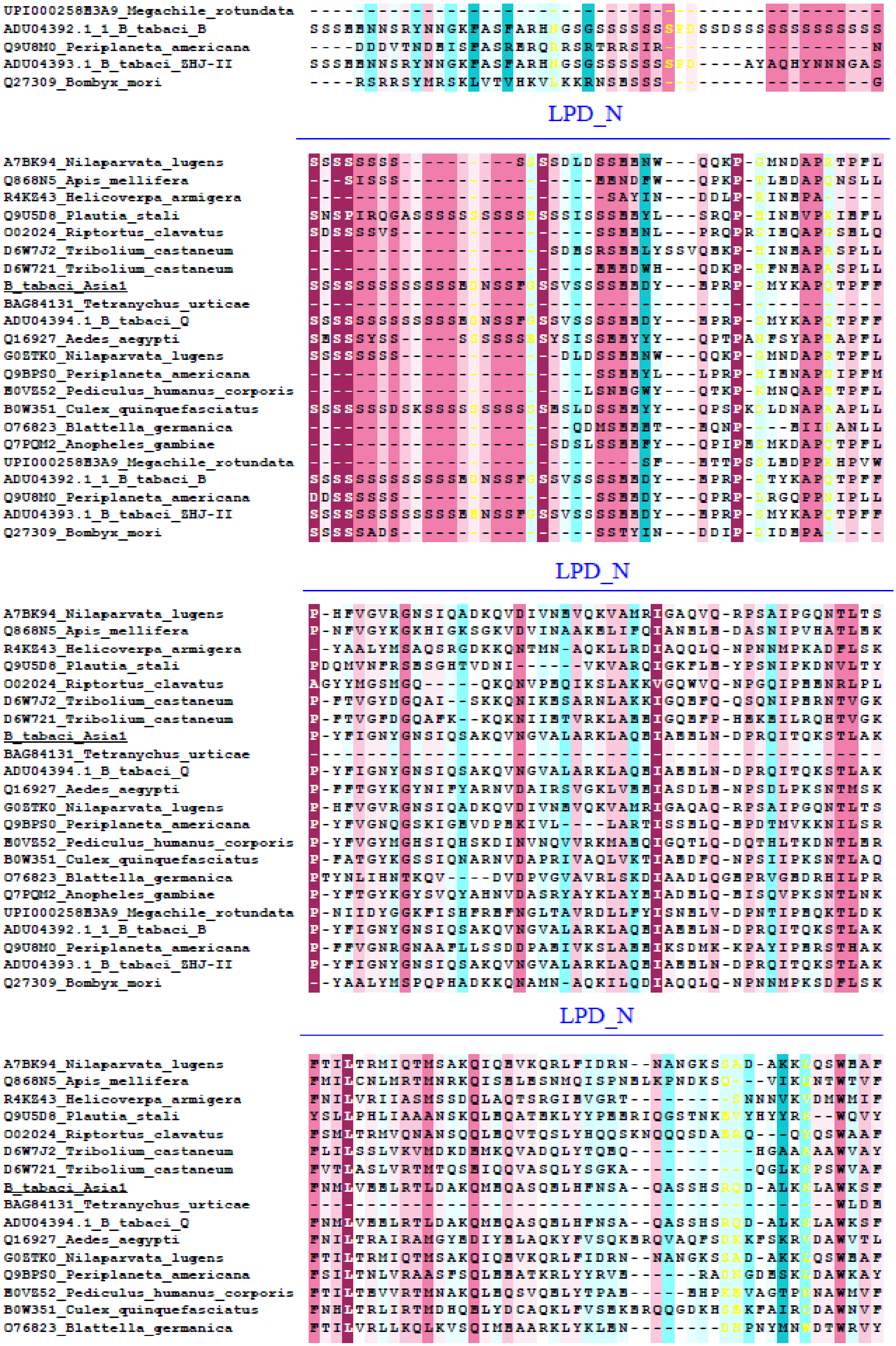
LPD_N
LPD_N
LPD_N

```
Q7PQM2_Anopheles_gambiae             FTILARVLRTMHYQDIYDVCQKLFVSQKEREEGSNHSESFAKKVDAWNTF
UPI000258E3A9_Megachile_rotundata    FTHLVNIIRTMNRNQIAEAENKWQISPKKLKWGDKAD---AFRQNRWSVF
ADU04392.1_1_B_tabaci_B              FNMLVEELRTLDAKQMEQASQELHFNSA--QASSHSRQD-ALKSLAWKSF
Q9U8M0_Periplaneta_americana         LMMMRDIVRTMTAKQLQKATSLIHSESK------------HDLGWIAY
ADU04393.1_B_tabaci_ZHJ-II           FNMLVEELRTLDAKQMEQASQELHFNSA--QASSHSRQD-ALKSLAWKSF
Q27309_Bombyx_mori                   FNILVRLIASMSTEQLSQTSRSIETAKT--------SNNIIKSDMWMIF
```

LPD_N

```
A7BK94_Nilaparvata_lugens            KEATANAGTGPALEAIKNWVEKGDVRNEKAAELVAVLPRTARLPTDQYIK
Q868N5_Apis_mellifera                RDAITQTGTGPAFLTIKEWIERGTTKSMEAANIMSKLPKTVRTPTDSYIR
R4KZ43_Helicoverpa_armigera          RDAVVQAGTPPAFLQIKTWILNKKLQNEEAAQVISTLARTLRYPTKEIMI
Q9U5D8_Plautia_stali                 RDAVAQAGTGPALVTIYSWLKSAKVRENEGAQLLAVLPKAARYPTTEYMD
O02024_Riptortus_clavatus            RDAVAQAGTGPALVTIKQWIQSKKVQGEEAAQLLAVLPYTARYPNTEYMN
D6W7J2_Tribolium_castaneum           RDAVAEAGTGPALLNIQEWIREGKISREEAAEVVSTAAVSARQPTEKYMK
D6W721_Tribolium_castaneum           RDAVAQAGTGPALYNIKEWILSGKIDGREAAQVIAVAANAARQPTEEYIK
B_tabaci_Asia1                       CDALVEAGTGPAFLQIQKIIEHQQVSDAEAARMISRLPVTARFPDKEYMN
BAG84131_Tetranychus_urticae         CEFIA---------------------------------------------
ADU04394.1_B_tabaci_Q                CDALVEAGTGPAFLQIQKII----------------PYTTMFNTRTNST
Q16927_Aedes_aegypti                 RDAVAEAGTPSAFKLIFDFIKEKKLRGYEAATVIASLAQSIRYPTEHLLH
G0ZTK0_Nilaparvata_lugens            KEATANAGTGPALEAIKNWVEKGDVRNEKAAELVAVLPRTARLPTDQYIK
Q9BPS0_Periplaneta_americana         RDSVAQAGTPAALKMVHTWIRKEYIKDEEAAKVVAVIPHAADTPTDNYIA
E0VZ52_Pediculus_humanus_corporis    RDVLAQAGTGPALTTIVSLITSKKLKGEEAAQVVSTLADTARYPTTEYLN
B0W351_Culex_quinquefasciatus        RDAMAEAGTPPVFKVIKQYIEEKKLRGIEAASVVATLPKRIRYPTETLMH
O76823_Blattella_germanica           RDAVSQAGTWSALNSIQQFISSEMVEPKEASHLITVLPAAVSDKNKAYLH
Q7PQM2_Anopheles_gambiae             RDALAQAGTPPAFKVIKELIEEKKLRGDEAASVIATLPKTIRYPTETVMH
UPI000258E3A9_Megachile_rotundata    RDAITQAGTGPALLTIKNWVENGDLRSEEAAEMLSKIPASARQITAKYIE
ADU04392.1_1_B_tabaci_B              CDALVEAGTGPAFLQIQKIIEHQQVSDAEAARMISRLPVTARFPDKEYMN
Q9U8M0_Periplaneta_americana         RDMVSESGTHPALEELSIWIISKKLSSEEGAELLATLPRAVIMPTPEYFE
ADU04393.1_B_tabaci_ZHJ-II           CDALVEAGTGPAFLQMQKIIEHQQVSDAEAARMISRLPVTARFPDKEYMN
Q27309_Bombyx_mori                   RDGVTQAGTLPAFKQIQSWIENKKIQEEEAAQVVVALPRTLRYPTKQIMT
```

LPD_N

```
A7BK94_Nilaparvata_lugens            TFFQFATSSNVQNQKYLNSTIILGFSEILRKAQVDSDTKHMRFGVHSFGH
Q868N5_Apis_mellifera                SFFELLQNPKVSNEQFLNTAATLSFCEMIHNAQVNKRSIHNNYPVHTFGR
R4KZ43_Helicoverpa_armigera          QFFELAMNPVVQEQERLNTSALIAATKFIYMGQVNNETAHYYYPSHMYGR
Q9U5D8_Plautia_stali                 YLFGMVKSKEIQNQKYLNDSAVLSFADLVRRSQVDKKSAHQRYPIHLYGA
O02024_Riptortus_clavatus            YFFDMVTSEEIQRQRSLNTTALFAFTELSRKSQVDYDVAHNRYPVHLHAK
D6W7J2_Tribolium_castaneum           VYFEMIQNDQIMEQPYLNESLLLAYTTLIHQVYVNEEYSHSKYPVHSFGS
D6W721_Tribolium_castaneum           FFNDMIQDEKIMSQQHLNESALLSYTNLVRQVYANRGDSHAKYPVYSFGS
B_tabaci_Asia1                       SFFNFVRSNNVQHQNQLNETALLAFAELCRKADVNARNAHNYYPVHVYGR
BAG84131_Tetranychus_urticae         --------------------------------------------------
ADU04394.1_B_tabaci_Q                KLLSLLS-----------------LSFARKADVNARNAHNYYPVHVYGR
Q16927_Aedes_aegypti                 EFFLLVTSDVVLHQEYLNATALFAYSNFVNQAHVSNRSAYNYYPVFSFGR
G0ZTK0_Nilaparvata_lugens            TFFQFATSSNVQNQKYLNSTIILGFSEILRKAQVDSDTKHMRFGVHSFGH
Q9BPS0_Periplaneta_americana         YFFEMVKDPVVHGEKYLNSSAVLAFSKLLRLAAVDSEAVR-RYPVHVFGR
E0VZ52_Pediculus_humanus_corporis    TFFELVKHPEVVSQPILRTTSVYTFTKLVRYALVNPKTVHNRYPVHTFGY
B0W351_Culex_quinquefasciatus        EFFLLATSTAVQHQETLNATALIAFSDFLNRAHVNNQSALNYYPVNSFGR
O76823_Blattella_germanica           FLFEMTKDPVFKNMTYVNTSLVLAFSEVIHQVEM----------------
Q7PQM2_Anopheles_gambiae             EYFLLVTSNAVQHQEYLNTTALISYCDFLNRAQVNNRSAYNYYPVYSFGR
UPI000258E3A9_Megachile_rotundata    AFFELATHPQVTQTALLNVTAITAFSELLFLSQGNDKSIYKFYPVHTTDR
ADU04392.1_1_B_tabaci_B              SFFNFVRSNNVQHQNQLNETALLAFAELCRKADVNARNAHNYYPVHVYGR
Q9U8M0_Periplaneta_americana         AFNKLVMDKRVRNQPIVNSTGLLALATLHRQVH-DAEFSHNNYPVHAFGR
ADU04393.1_B_tabaci_ZHJ-II           SFFNFVRSNNVQHQNQLNETALLAFAELCRKADVNARNAHNYYPVHVYGR
Q27309_Bombyx_mori                   QFFNFARSPAVKDQMFLNSSALMAATKLINLGQVNNYTAHSYYPTHMYGR
```

LPD_N

```
A7BK94_Nilaparvata_lugens            L-TSKHDQSLHQEYMPYLEEKLKSAFEKGDSQKIIVYIQALGNTAHPRLL
Q868N5_Apis_mellifera                L-TSKHDNSLYDEYIPFLERELRKAHQEKDSPRIQTYIMALGMIGEPKIL
R4KZ43_Helicoverpa_armigera          L-ARRNDRFVLDDILPRLSEKLQQAIEKQEWSRAQVYIKAIGNLGHREIL
Q9U5D8_Plautia_stali                 E-ALNHFAVVIEKYIPYFQGQLRKAVQKGDSIQIQVYIRALANMGHPTVW
O02024_Riptortus_clavatus            N-SQEHRQAVPQKYIPWLQERLERAVSRGDSIKAQVYIRALGNTAHPKIL
D6W7J2_Tribolium_castaneum           FNTAQGWFYVKKEVIPYLSQKLDEAISNADTKQIHLYIRALGNVGHQQIL
D6W721_Tribolium_castaneum           FRTTRGQEYVKKNVIPHLTRKLNEAINSADTQKIHIYIRALGNVGHQQIL
B_tabaci_Asia1                       V-LPEHAKAVAHQYLPYYEQNLKRAVANGDSRKIQAYIRAIGNFAHPKIL
BAG84131_Tetranychus_urticae         -----------------------SMDCSQSQKFAVTVK--GQAGKRTV-
ADU04394.1_B_tabaci_Q                V-LPEHAKAVAHQYLPYYEQNLKRAVANGDSRKIQAYIRAIGNFAHPKIL
Q16927_Aedes_aegypti                 L-ADADYKIIEHKIVPWFAHQLREAVNEGDSVKIQVYIRSLGNLGHPQIL
G0ZTK0_Nilaparvata_lugens            L-TSKHGQSLHQEYMPYLEEKLKSAFEKGDSQKIIVYIQALGNTAHPRLL
Q9BPS0_Periplaneta_americana         M-VPKNFSARVKEYIEYFANKLKNAVKDKDSHKIQVYTRALGNTGHADII
E0VZ52_Pediculus_humanus_corporis    L-TRLDDTTVTDTYVPYLAQQLEQAVKEGDSTKVQTYVVGLGNVAHPKIL
B0W351_Culex_quinquefasciatus        L-ADSKYKIVAHKAVPWLAHQLREAVQEADSERIQVYIRAIGNLGHPEIL
```

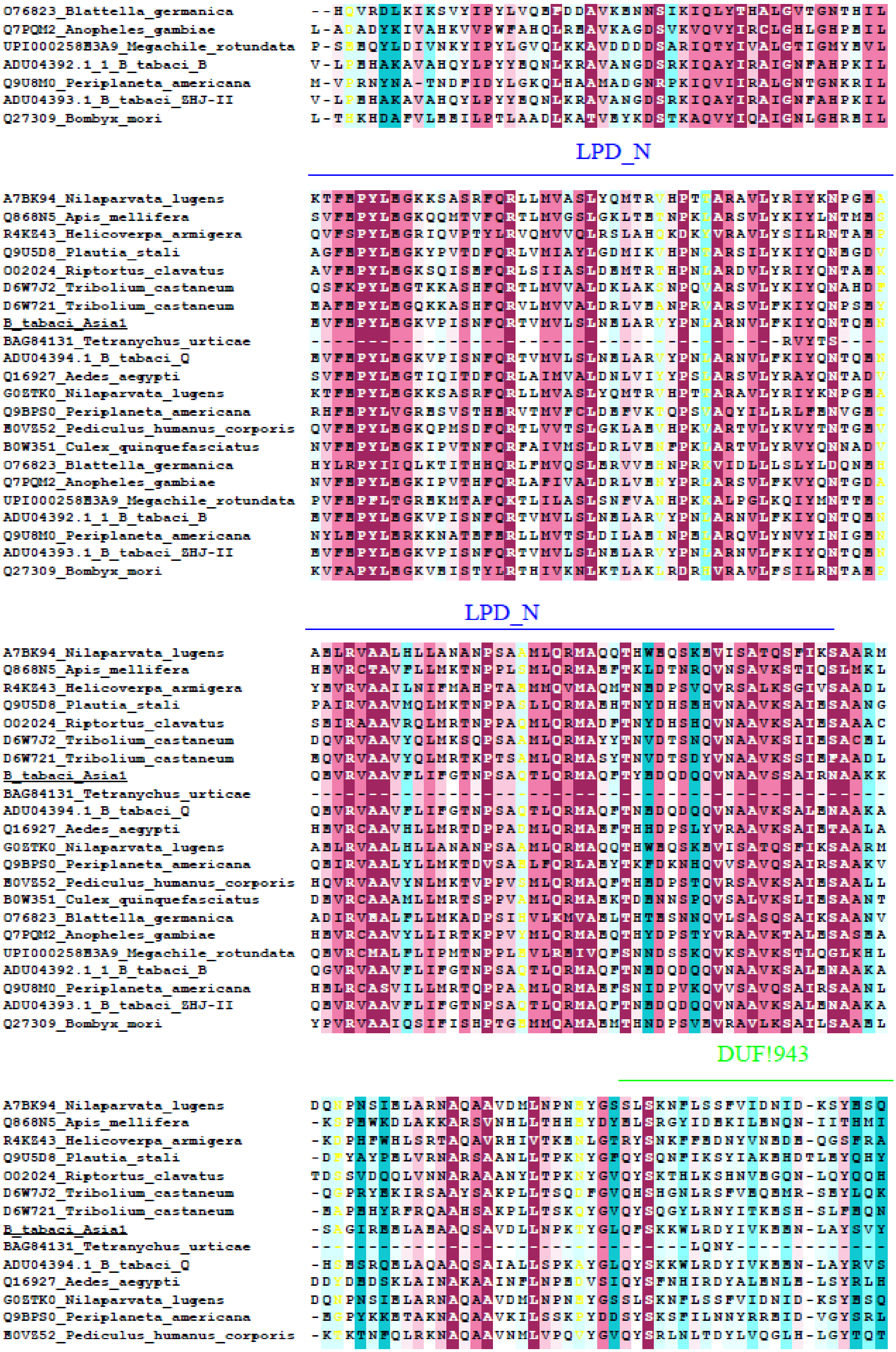
LPD_N
LPD_N
DUF!943

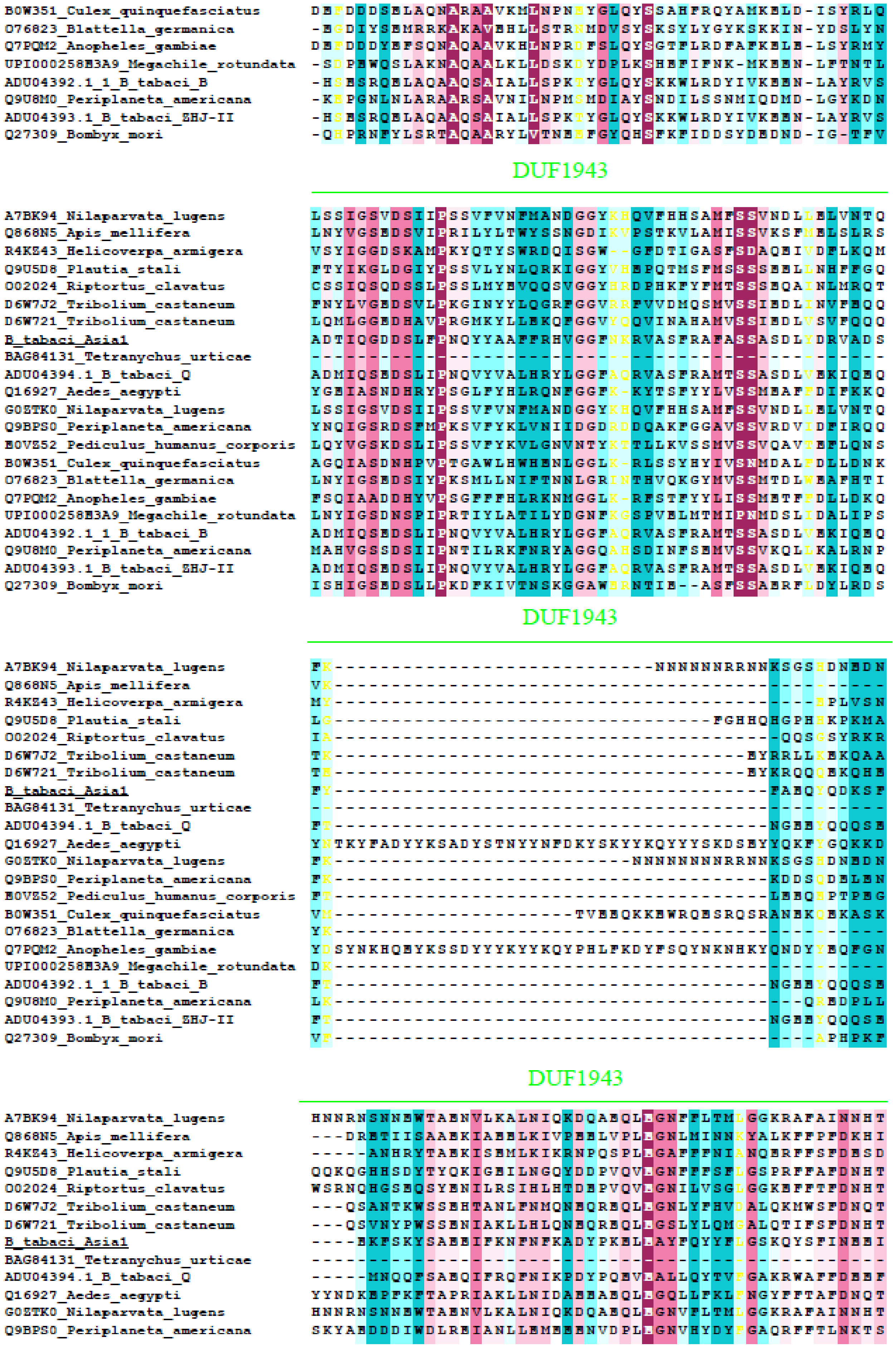
DUF1943
DUF1943
DUF1943

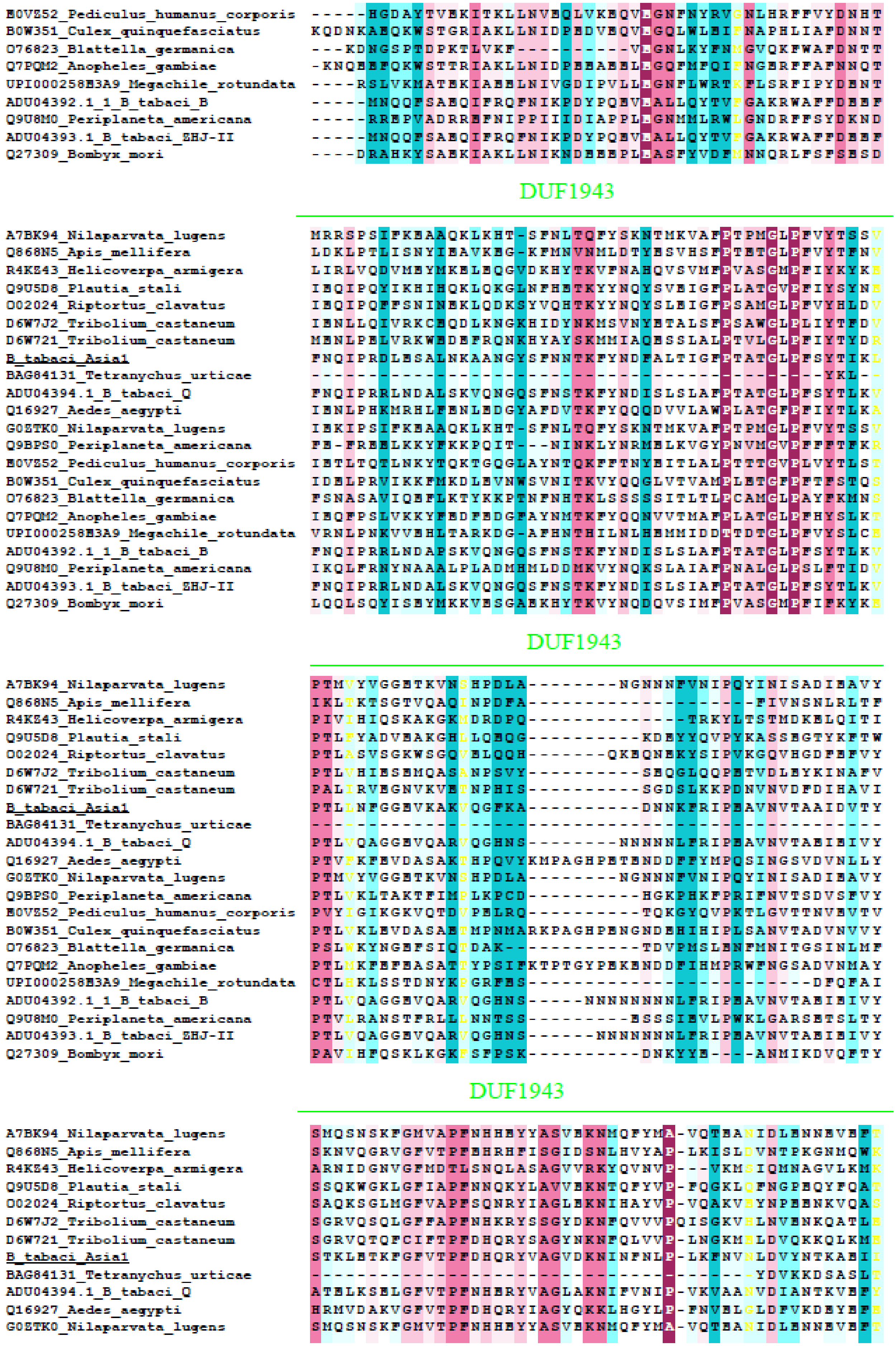
DUF1943
DUF1943
DUF1943
B0VZ52_Pediculus_humanus_corporis
B0W351_Culex_quinquefasciatus
O76823_Blattella_germanica
Q7PQM2_Anopheles_gambiae
UPI000258E3A9_Megachile_rotundata
ADU04392.1_1_B_tabaci_B
Q9U8M0_Periplaneta_americana
ADU04393.1_B_tabaci_ZHJ-II
Q27309_Bombyx_mori
A7BK94_Nilaparvata_lugens
Q868N5_Apis_mellifera
R4KZ43_Helicoverpa_armigera
Q9U5D8_Plautia_stali
O02024_Riptortus_clavatus
D6W7J2_Tribolium_castaneum
D6W721_Tribolium_castaneum
B_tabaci_Asia1
BAG84131_Tetranychus_urticae
ADU04394.1_B_tabaci_Q
Q16927_Aedes_aegypti
G0ZTK0_Nilaparvata_lugens

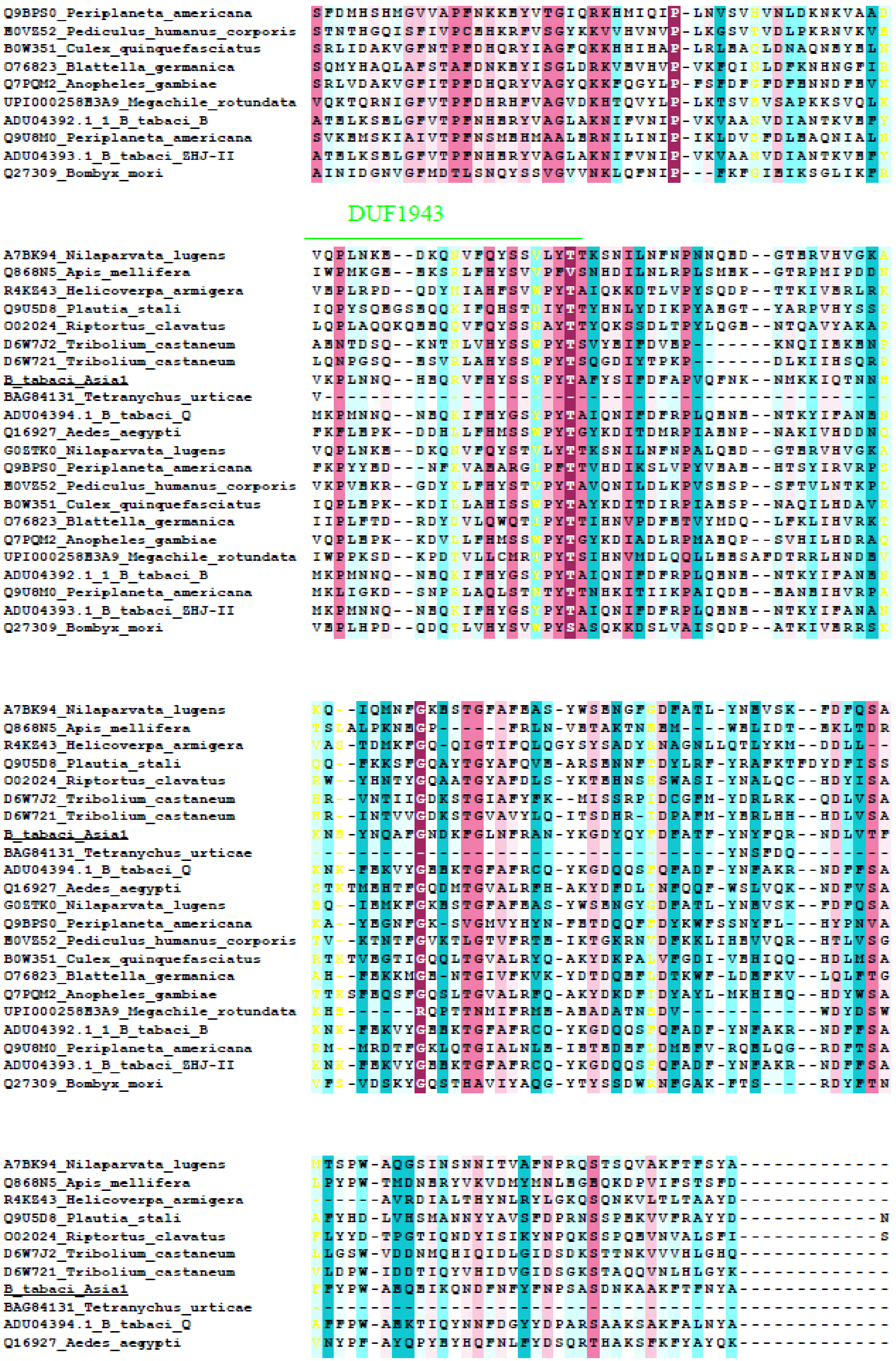
Q9BPS0_Periplaneta_americana
E0VZ52_Pediculus_humanus_corporis
B0W351_Culex_quinquefasciatus
O76823_Blattella_germanica
Q7PQM2_Anopheles_gambiae
UPI000258E3A9_Megachile_rotundata
ADU04392.1_1_B_tabaci_B
Q9U8M0_Periplaneta_americana
ADU04393.1_B_tabaci_ZHJ-II
Q27309_Bombyx_mori
DUF1943
A7BK94_Nilaparvata_lugens
Q868N5_Apis_mellifera
R4KZ43_Helicoverpa_armigera
Q9U5D8_Plautia_stali
O02024_Riptortus_clavatus
D6W7J2_Tribolium_castaneum
D6W721_Tribolium_castaneum
B_tabaci_Asia1
BAG84131_Tetranychus_urticae
ADU04394.1_B_tabaci_Q
Q16927_Aedes_aegypti
G0ZTK0_Nilaparvata_lugens
Q9BPS0_Periplaneta_americana
E0VZ52_Pediculus_humanus_corporis
B0W351_Culex_quinquefasciatus
O76823_Blattella_germanica
Q7PQM2_Anopheles_gambiae
UPI000258E3A9_Megachile_rotundata
ADU04392.1_1_B_tabaci_B
Q9U8M0_Periplaneta_americana
ADU04393.1_B_tabaci_ZHJ-II
Q27309_Bombyx_mori
A7BK94_Nilaparvata_lugens
Q868N5_Apis_mellifera
R4KZ43_Helicoverpa_armigera
Q9U5D8_Plautia_stali
O02024_Riptortus_clavatus
D6W7J2_Tribolium_castaneum
D6W721_Tribolium_castaneum
B_tabaci_Asia1
BAG84131_Tetranychus_urticae
ADU04394.1_B_tabaci_Q
Q16927_Aedes_aegypti
G0ZTK0_Nilaparvata_lugens
Q9BPS0_Periplaneta_americana
E0VZ52_Pediculus_humanus_corporis
B0W351_Culex_quinquefasciatus
O76823_Blattella_germanica
Q7PQM2_Anopheles_gambiae
UPI000258E3A9_Megachile_rotundata
ADU04392.1_1_B_tabaci_B
Q9U8M0_Periplaneta_americana
ADU04393.1_B_tabaci_ZHJ-II
Q27309_Bombyx_mori
A7BK94_Nilaparvata_lugens
Q868N5_Apis_mellifera
R4KZ43_Helicoverpa_armigera
Q9U5D8_Plautia_stali
O02024_Riptortus_clavatus
D6W7J2_Tribolium_castaneum
D6W721_Tribolium_castaneum
B_tabaci_Asia1
BAG84131_Tetranychus_urticae
ADU04394.1_B_tabaci_Q
Q16927_Aedes_aegypti

```
G0ZTK0_Nilaparvata_lugens              MTSPW-AQGSLNSNNITVAFNPRQSTSQVAKFTFSYA-------------
Q9BPS0_Periplaneta_americana           FYYGW-EAQPVFYYDFKLYLDSHNSPAKTVQLKASYD--------NRYTQP
E0VZ52_Pediculus_humanus_corporis      VVFPT-AEATIDNTNVDVYYDSKLSTVQSLVLSATYR-------------
B0W351_Culex_quinquefasciatus          LLFPLHASQPCHYHQLNLWYDAQRSPVKNIKLSLQQT-------------
O76823_Blattella_germanica             LNYDV-PTKDIFYNNLTVYYDHEDTKNHAVSFTVTKEQSKFYETLNPVVQ
Q7PQM2_Anopheles_gambiae               LVYPF-ASETYHYHQFNLYYDAQRTSVKNVKFVLQHK-------------
UPI000258E3A9_Megachile_rotundata      IMFPI-KDKQSEYHKVAVIMDKRSDEGKPLVLSASYG-------------
ADU04392.1_1_B_tabaci_B                AFFPW-AEKTIQYNNFDVYYDPARSAAKSAKFALNYA-------------
Q9U8M0_Periplaneta_americana           LAFIW-ARRTINNHNVTLSIDEDQSTTNAVRIEGKYA-------------S
ADU04393.1_B_tabaci_ZHJ-II             AFFPW-AEKTIQYNNFDVYYDPARSAAKSAKFALNYA-------------
Q27309_Bombyx_mori                     LASLL-TQEDIALTHFNLKHLCKQSQSKALTITAYYD-------------

A7BK94_Nilaparvata_lugens              -DNSDDNNNSHSGHDSNNSNNNNNNRADYSDAQPSSTTGQTTLMLSHLPL
Q868N5_Apis_mellifera                  -----------SKVMTRPDTDSENWTPKMMAVEPTDKQ------------
R4KZ43_Helicoverpa_armigera            -----------------QLFNQKQSGELGQADKRKDVT------------
Q9U5D8_Plautia_stali                   V--------EQGGQHKKNAKLMNARGKRINAGGSNLAQ------------
O02024_Riptortus_clavatus              NEYGNKGARMTSKFKKANIRDRSASSARDNLAVPSSEQ------------
D6W7J2_Tribolium_castaneum             ------------------HTYNSNLNRQPAPEMSSRYQ------------
D6W721_Tribolium_castaneum             ------------YKYQSTQAQEKNANDQITDIPAFADS------------
B_tabaci_Asia1                         -----------SKYAAKEQADHESRNANTNDAVTSNNK------------
BAG84131_Tetranychus_urticae           --------------------------------------------------
ADU04394.1_B_tabaci_Q                  --------NKYANKENNNEGNSNNHNNNHNDAVPSSYQ------------
Q16927_Aedes_aegypti                   --FGAPSFEETGPKHPANRHSYSGNYYESNYAQPFVYS------------
G0ZTK0_Nilaparvata_lugens              -ENSDDNNNSHSGHDSNSSNNNNNNRADYSDAQPSSTA------------
Q9BPS0_Periplaneta_americana           EEEEETRQHSKIRRPRSASRKHRRSRHEERAPLENLEV------------
E0VZ52_Pediculus_humanus_corporis      ------------------QVTPTEIKHETDTGKVFVPT------------
B0W351_Culex_quinquefasciatus          TANETEDFSSSDIKHPKARHQSEGYYNEKNLAQPFVFK------------
O76823_Blattella_germanica             QNLKLSSGKKQKHRNVKSHRIRREYTEDENPAIPKDKQ------------
Q7PQM2_Anopheles_gambiae               QADYDQDFQTADVKHPKSRHGFSGFYNEYNYAQPFVYY------------
UPI000258E3A9_Megachile_rotundata      -----------TLDVEAGTEDANQWTQFVNATEAFSEI------------
ADU04392.1_1_B_tabaci_B                --------NKYANKENNNEGNSNNHNNNQNDAVPSSYQ------------
Q9U8M0_Periplaneta_americana           KINDVDAGTWTEWRSSRVERSAKRHHRPRPEAQEYDSS------------
ADU04393.1_B_tabaci_ZHJ-II             --------NKYANKENNNEGNSNNHNNNQNDAVPSSYQ------------
Q27309_Bombyx_mori                     -----------------EYYNQQNSGILTDATDRNDLS------------

A7BK94_Nilaparvata_lugens              LPTVV------SRQNEFLRKAAAGISGADAMVVDVSARFQDSHGQSNAQY
Q868N5_Apis_mellifera                  -ANSK------TRRQEMMREAGRGIESAKSYVVDVRVHVPG-ESE--SET
R4KZ43_Helicoverpa_armigera            -PNSE------ARREEMLKRVSAGINNARAQVVDFSATFEG-PQK--QEY
Q9U5D8_Plautia_stali                   -PSSV-----ENRVDELFTNSYSGIQDARAQVFDASVTFEGGQNK--GQY
O02024_Riptortus_clavatus              -----------QREQELLRNVEAEIQDAKSHLLDASVTFGGKNNN--AGY
D6W7J2_Tribolium_castaneum             -AAQESW----ERQKEFLKNCSKDIRAVRAHVVDAIVLFEG-QRI--VKY
D6W721_Tribolium_castaneum             -PE--------KRQQEFVDKLGKYINNPHVFVADATADFQG-QYK--VKY
B_tabaci_Asia1                         -PDSE------ERLNEFVRKSYAGINSAFVNAFDFSAQFLG-QKE--ADY
BAG84131_Tetranychus_urticae           ------------------------------------------QQG--PDY
ADU04394.1_B_tabaci_Q                  -PDNE------QRMNQFASRAQSGVQSANIDVIDISAQFFG-QKN--ADY
Q16927_Aedes_aegypti                   -PGSQ------RRYEQFFRNAASGIRNSFVRYYDFGFEFYAPQYK--SEF
G0ZTK0_Nilaparvata_lugens              -ANSR------SRQNEFLRKAAAGISGADAVVVDVSARFQGSHGQSNAQY
Q9BPS0_Periplaneta_americana           -SDTE------TQREELYDIVLPAVRAGRLYYASVSVAFKG-EENVYSKY
E0VZ52_Pediculus_humanus_corporis      -SDVQ------TRIKQFYEKVTTGVKGARAQVCDFVLNVNA-QTP--VEL
B0W351_Culex_quinquefasciatus          -PASQ------RRQEQFLKNAGAGIRNSLVSVWDLGAEFEGRQNK--AEF
O76823_Blattella_germanica             -PNSH------PRRQEYLSKSMALTGDATAVVLDMTLKFEG-PAE--SYF
Q7PQM2_Anopheles_gambiae               -AGSQ------RRQEQFMRNAGAGIRNSDVNVYDFGIVFEGKQQK--AEF
UPI000258E3A9_Megachile_rotundata      -RDSE------QRKIRFLEEAAKGIVLAQAGVLDFHIHIPFINEE--VKT
ADU04392.1_1_B_tabaci_B                -PDNE------QRMNQFASRAQSGVQSANIDVIGISAQFFG-QKN--ADY
Q9U8M0_Periplaneta_americana           -ANPAIVDRA-DKLEEFLNRAS----GNRVHGLAVRVAFTG-SSD--ATF
ADU04393.1_B_tabaci_ZHJ-II             -PDNE------QRMNQFASRAQSGVQSANIDVIDISAQFFG-QKN--ADY
Q27309_Bombyx_mori                     -PNSE------TRRAEMVKLVSAGINKARVRVVDLSASFEG-SQD--QNY

A7BK94_Nilaparvata_lugens              VATVAMANSDASPNARMLFFASMNPAN-----------SDSKAQVCAAAA
Q868N5_Apis_mellifera                  VLTLAWSESNVESKGRLLGFWRVEMPR-----------SNADYEVCIGSQ
R4KZ43_Helicoverpa_armigera            VLTAAIAKSPVDLKIQSAVFAGRNSV------------QQGDEQINAVVK
Q9U5D8_Plautia_stali                   AFTFAHAESPVSEKSRYLAYFSASPYQESQ--------YAKAFKAAFHLN
O02024_Riptortus_clavatus              SFTLAHANSPVSEKSRVLFYYNAQPVSSSE--------NTKPFQAAFQLD
D6W7J2_Tribolium_castaneum             TSTLAGARSDIDPRGRVLMQMEKDNQ------------HDKPMRVFFAAN
D6W721_Tribolium_castaneum             SATFGVAKSNVDPQSRFMGYARKVEK------------QNPQVLAYVQGV
B_tabaci_Asia1                         VCTFAFARSPVAEKSRFLFYGHYNTA------------NNKKQQCAFHAS
BAG84131_Tetranychus_urticae           VLT-----------------------------------------------
ADU04394.1_B_tabaci_Q                  VATLAYARSPVAEKARFLFYAGANNA------------NNNKNKVAVDAT
```

```
Q16927_Aedes_aegypti                 TFTTAFADSPVDKTSRQLYYFYASPMFPPSQSYFKDIPFSGKQFQFCATAT
G0ZTK0_Nilaparvata_lugens            VATVAMANSDASSNARMLFFASMNPAN------------SDSKAQVCAAVA
Q9BPS0_Periplaneta_americana         EVEGALASSQVNEHISTMLRAHSNDA-------------ERKHQYAHVRVN
E0VZ52_Pediculus_humanus_corporis    VGTFALARSPVDAPVNLIGYFYKTA--------------GNDHYEICYDST
B0W351_Culex_quinquefasciatus        VLTFAKASSPVDEKERTLVFASASPYIAVG---------SKKQHQACLSLT
O76823_Blattella_germanica           TTTVSHATSLVNGSSNYLLFYDQHYYE------------EKKRNQFCLSWS
Q7PQM2_Anopheles_gambiae             VFTTAYADSPVDEKERLLMFLSFSPYVSSSAFFEFIPFSGKQFQMCFSAT
UPI000258E3A9_Megachile_rotundata    NLTVAWSASNAETKGRFLSRLTIMNQI------------DEVEFEVCAASQ
ADU04392.1_1_B_tabaci_B              VATLAYARSPVAEKARFLFYAGANNA-------------NNNKNKVAVDAT
Q9U8M0_Periplaneta_americana         DLTAALGLSNVNGSARALVSYISQPAHPADV--------MPRKTEYDLFAA
ADU04393.1_B_tabaci_ZHJ-II           VATLAYARSPVAEKARFLFYAGANNA-------------NNNKNKVAVDAT
Q27309_Bombyx_mori                   VLTGTWGDSPVDSKVQGMLFAGTKSA-------------TQGNQQINAVFA


A7BK94_Nilaparvata_lugens            SNFPNVPLMNFHDALKANPTSRISADIAFGAQCNAGGHIHADAKLSQTQE
Q868N5_Apis_mellifera                IMVSPETLLSYDEKMDQKPKMDFNVDIRYGKNCGKGERIDMNGKLRQSPR
R4KZ43_Helicoverpa_armigera          VTKPEISALNFLEALKKDVKMTYEADIKYGQD----GNIHIQGNTERTKK
Q9U5D8_Plautia_stali                 FKFPQVPVFNYKKALESDPTSYVEGQINFNDN--EKYKIAFQGKFEQTEE
O02024_Riptortus_clavatus            IKDPQLPAFNYQDALNADSTSQVQGKINMGEQLE--SQITFKGKLERTQE
D6W7J2_Tribolium_castaneum           TKIPNTNSLNLKYALEFDPTSTIQIQGVIEKKSGNDAQINAKLTLAKSEE
D6W721_Tribolium_castaneum           SHVRNTNGLNLDYAMEFDPTSTAYIQGLLRKE-QRLSRVDAHIELSKSEQ
B_tabaci_Asia1                       AEMPNVPLTNPAAAMKAEPASKIYANFKFGESFENAAKVHFNANLKQSSE
BAG84131_Tetranychus_urticae         ---PGKPAI-----------------------------------------
ADU04394.1_B_tabaci_Q                AYMPNVPLVNAAQAFNADANSRLYVNVKSGENLDNGAQFQFQANMKQSQE
Q16927_Aedes_aegypti                 SEFPRVPYLKFSDFDKYYGDASQYFDFLYGESCQGGAHIAVKGKQKQTGK
G0ZTK0_Nilaparvata_lugens            SNFPNVPLMNFLDALKANPTSHISADIAFGAQCNAGGHIHADARLSQTQE
Q9BPS0_Periplaneta_americana         VTMPQVPVIDYRKALEFDPTSKIQCEVHFGDTPEKKSKVYFQGKFERTDE
E0VZ52_Pediculus_humanus_corporis    FKSTYSPIYDLEHAIQSESKSDLNVHVKFGNKCDTGAKVVLTGKLERTPE
B0W351_Culex_quinquefasciatus        EKYPSVPMLNYITALQNDVTSEIDLELSFGEKCAGGAQVSVNGMLRQTDL
O76823_Blattella_germanica           VYKPQVPIMNIYSAFEFDPNSKVHAIMNIGKECENGGSAVANIDMLRLSE
Q7PQM2_Anopheles_gambiae             NQYPNMPKLNFLNVLNFDKVGSMNWELAYGEKCQGGSHVSMKGKLIQSEP
UPI000258E3A9_Megachile_rotundata    LALTPPYFPVYDEVNNCTPKIEFDIDIRHGENCQEDNRVNIIGTGTRSNE
ADU04392.1_1_B_tabaci_B              AYMPNVPLVNAAQAFNADANSRFYANVKSGENLDNGAQFQFQANMKQSQE
Q9U8M0_Periplaneta_americana         LSMPAPPIINFNQALEFDPDSNLDAGLSIFTNDKPSGNLRIKGELQQSEE
ADU04393.1_B_tabaci_ZHJ-II           AYMPNVPLVNAAQAFNADANSRFYANVKSGENLDNGAQFQFQANMKQSQE
Q27309_Bombyx_mori                   TTKPEIHSLSFSKPLQSDLRAPFGMHFKYGQS----GEIRVSGSFDRTKK


A7BK94_Nilaparvata_lugens            FQEYAKSRPMAKKCFQLMEKG-QALEYACQNATKVANMLNNYEVSVKYD-
Q868N5_Apis_mellifera                LKELVGATSIIKDCVEDMKRG-NKILRTCQKAVVLSMLLDEVDISMEVP-
R4KZ43_Helicoverpa_armigera          YTELLQKHPLAKLVQEQIANG-NQYQLASHKMLIKAYAPDSLKASVTYK-
Q9U5D8_Plautia_stali                 RKNYVKHSQYSAECLEQMQHD-NYIQPVCLNASLNRNALDKYEFTFKFQ-
O02024_Riptortus_clavatus            RKEYLQQHPLARACESQMQRR-NYIQPACLNATLQANFHNRYQFVFNYQ-
D6W7J2_Tribolium_castaneum           RKQYLKELPQYEKCQKEMQEG-NYQLPACAMLTAQANLLDQFSFEMNYH-
D6W721_Tribolium_castaneum           RKEYLKQQEEYKECKHQMQEG-NTQLPICAKMGIRANLLDTFSIKMKYS-
B_tabaci_Asia1                       RRQFLRNNALYKQCESEMERG-QYFLPACRNFTVADNRMNEYYYNFNFQ-
BAG84131_Tetranychus_urticae         --------------------------------------------------
ADU04394.1_B_tabaci_Q                FRDYFRQSQMYKQCSQQMEQG-EYMMPACRNATVAANRLNEAHFSINFD-
Q16927_Aedes_aegypti                 YREYLRFSDVAKACKEQMANG-YYQFEECQQAIDQAYYYDFYDYAIEYK-
G0ZTK0_Nilaparvata_lugens            FQEYAKSRPMAKKCFQLMEKG-QALEYACQNATKVANMLNNYEVSVKYD-
Q9BPS0_Periplaneta_americana         RKKFVAESDMAQLCSAQQNNK-NYLLPACRNVTEEASKLDKYFFKVKYE-
E0VZ52_Pediculus_humanus_corporis    RQQYVQTLPQVQTCKTEVQQG-NKYTANCRNATLQAGLFDKYTFNIKYE-
B0W351_Culex_quinquefasciatus        WRETLRSSAIGRKCKNQMAEG-YFALPECQNATRLASALDHYTFDIEFK-
O76823_Blattella_germanica           HLDYVKNLTVSKLCDHEMRTKRDHVLPACRNSTERASDLNRVHVDINYNL
Q7PQM2_Anopheles_gambiae             YRHFLRISEAGQSCKQQMDQG-YFQLPACQNATRQAGYFDQYSFNFEYK-
UPI000258E3A9_Megachile_rotundata    LKEDINDISVVKECKKQMEQG-NKLLRACQKAGDKALQVDELQLSLQS--
ADU04392.1_1_B_tabaci_B              FRDYFRQSQMYKQCSQQMEQG-EYMMPACRNATVVANRLNEAHFSINFD-
Q9U8M0_Periplaneta_americana         RRNAIRSTPAALACMREMANGNNNLLPSCRNATEMANRLDRIRLQAKFE-
ADU04393.1_B_tabaci_ZHJ-II           FRDYFRQSQMYKQCSQQMEQG-EYMMPACRNATVAANRLNEAHFSINFD-
Q27309_Bombyx_mori                   YTTELENHPLAKQCSQQTTLN-NFYQDSCHKAIVMAHAPDHVEFSVSFQ-


A7BK94_Nilaparvata_lugens            RVSSVFKNVTYSIYSALAQAAYPYHNENMF--SQNSNPSGKIDLNARFNY
Q868N5_Apis_mellifera                --SDALIALYSQGLFSLSEIDNLDVSLDVS--NPKNAGKKKIDVRAKLNE
R4KZ43_Helicoverpa_armigera          NVSPMFMNWTSQAYNILKQLS---WNADVN--PMKRVADGKLQFDVQTSY
Q9U5D8_Plautia_stali                 NVPEYFQHLAYKAYSFARYEGYQYNQEEYA--GVKNEPG-QIKFGFNFSA
O02024_Riptortus_clavatus            NVPAGVRRAFHSAYSMGRYALYQNNEENIN---TEQNQEGQIQIGVQFEE
D6W7J2_Tribolium_castaneum           NIDKVVVNSTYKLYTVLRHYMYPRVSEDILMENEQVQKD-RLQVKGQFSP
D6W721_Tribolium_castaneum           DMDPRVINATYKTYSVLRHFLYPRVEENIV----EPSSDNHLDIQGQFSP
B_tabaci_Asia1                       NIPEYFKNYTYQAFAFARHMGYQYQSENVV--NPHYKPN-EIEGFFKFSP
BAG84131_Tetranychus_urticae         --------------------------------------------------
```

```
ADU04394.1_B_tabaci_Q                KVSDAVKNYTYQAFAYARHLGYQYQSENFG--NPNGQHN-KIDGYFKFSP
Q16927_Aedes_aegypti                 DVGSVAKNLTNKFYNYFQYAFYPYFESNFF---YHGKSN-YIKAEFEFAP
G0ZTK0_Nilaparvata_lugens            RVSSVFKNVTYSIYSALAQAAYPYHSENMF--SQNSNPSGKIDLNARFNY
Q9BPS0_Periplaneta_americana         NLSEKCRNRTYKAYSYLRHYFFPYITENVY---PDERKTDSVEVQVQFNE
E0VZ52_Pediculus_humanus_corporis    GVPEELKNYTYRVVNIYTHLAYPYLTEGMP--TQAAQQSKEVEFDVNFTP
B0W351_Culex_quinquefasciatus        EIPSSVRNMTNKALNWVQSAVITRWEEDCV---SHKGKEGKAQLKIELSP
O76823_Blattella_germanica           KQHETFKRRVYKVYDFVRTHLYPHVSEDVIVDNPAQ----FISANFTLKD
Q7PQM2_Anopheles_gambiae             DVSNYAKNLTYQFFDYARYFTFPYWNEDYF---FQGKHN-QFQIDFQLAP
UPI000258E3A9_Megachile_rotundata    GCRRTQRDMEYFFSHLLSSGIIVGTLAELQ---PKVMDEGKINLKAKISK
ADU04392.1_1_B_tabaci_B              KVSDAVKNYTYQAFAYARHLGYQYQSENFG--NPNGQHN-KIDGYFKFSP
Q9U8M0_Periplaneta_americana         NLSDDLINNTYKAYTWIRYFTQPYVTENIA----QEQNPGRLNINVDVNN
ADU04393.1_B_tabaci_ZHJ-II           KVSDAVKNYTYQAFAYARHLGYQYQSENFG--NPNGQHN-KIDGYFKFSP
Q27309_Bombyx_mori                   DMSPQYRNFSYHTYRLYEYLGYWYTEANPL----KLTQNGKMDFKIDFSY
```

```
A7BK94_Nilaparvata_lugens            NLRYFNASINTPFFSANVKNVEV-HHALRPL-----VIFHPSL-------
Q868N5_Apis_mellifera                YLDKADVIVNTPIMDAHFKDVKL-SDFG--F-----STEDILD-------
R4KZ43_Helicoverpa_armigera          VDQTLRFEMTSPSGVLRFDNVLI-PKFTPYM-----VSLYYPF-------
Q9U5D8_Plautia_stali                 DFDYFNVSLVTPVFTSRFENIGL-DRHLRPF-----FVQHPGY-------
O02024_Riptortus_clavatus            NLRSVNLSIAAPGLSSNFSNVRV-PGMAVPF-----VVYHPRY-------
D6W7J2_Tribolium_castaneum           CLSRVNASVVTEYGKVDFNNVKV-QDWAKAL-----LVSHPVH-------
D6W721_Tribolium_castaneum           DLHAVNFSINSEYGNVQVKNVQV-NKWYRDL-----FVSQPVF-------
B_tabaci_Asia1                       SFRYANFSFASPALSAAFDNVPV-NPYFAAI-----FAPHPTY-------
BAG84131_Tetranychus_urticae         --------------------------------------------------
ADU04394.1_B_tabaci_Q                KFDFAQFYFNAPSVAASFKNVPV-HQYVADF-----FAPHPVY-------
Q16927_Aedes_aegypti                 YGDYYNASFFGPSYAFQVQNYPVFNDYSTYFPYFFKYTFFPRYQPYYMHR
G0ZTK0_Nilaparvata_lugens            NLRYFNASINTPFFSANVKNVEV-HHALRPL-----VIFHPSL-------
Q9BPS0_Periplaneta_americana         EINAVNVSVKAPILNVEFTDVRVYNKYARAL-----FSLNPRY-------
E0VZ52_Pediculus_humanus_corporis    DLKNVNLYLSTPVGVSTFHSVPV-GEYTRTL-----LVSHPVF-------
B0W351_Culex_quinquefasciatus        RVSHINVTLATPNRKIEIENLPVENEWMKSL-----VLVHPDL-------
O76823_Blattella_germanica           NTRAFNVSIETPVLSVNATSVRL-QSWQSEM-----LRMNPRT-------
Q7PQM2_Anopheles_gambiae             YFDYYNASFYGSDRSFAIQNYPIESEYARYF-----FSVHPDF-------
UPI000258E3A9_Megachile_rotundata    NMKSANFSVQLPNINVEMNDVNL--DYFED----------SSE-------
ADU04392.1_1_B_tabaci_B              KFDFAQFYFNAPSVAASFKNVPV-HQYVADF-----FAPHPVY-------
Q9U8M0_Periplaneta_americana         DGTALNASVDTALMSITWTNIRL-NRWTRSL-----VEPSPQD-------
ADU04393.1_B_tabaci_ZHJ-II           NFDFAQFYFNAPSVAASFKNVPV-HQYVADF-----FAPHPVY-------
Q27309_Bombyx_mori                   FDRTYTVDIASPSGEARMRDMPI-ATMAPGA-----LSFYQPL-------
```

VWFD

```
A7BK94_Nilaparvata_lugens            --------------NSLEHLSSCPTMRTTIT-QHVLSA--RTPSAHSTTR
Q868N5_Apis_mellifera                --------------TADEDLLINNVFYEDET-SCMLDKT-RAQTFDGKDY
R4KZ43_Helicoverpa_armigera          --------------SMTERFANYYSSYQYQP-FCSIDGN-QVRTFSNRSY
Q9U5D8_Plautia_stali                 --------------NQLYYFAPDYFDTKNYA-TCGVDGNGVVTTFNGQSY
O02024_Riptortus_clavatus            --------------NTYQLLKSKLYQTEQPQATAVIDGN-KATTFSNRTY
D6W7J2_Tribolium_castaneum           --------------HVKSRLGGYALNYETYKANCMVDSC-AVNTLDNRTF
D6W721_Tribolium_castaneum           --------------HARARLQGQALKYDTYRPICVVDKT-QTSTWDNKTY
B_tabaci_Asia1                       --------------TAFDFFMQETFRSKYQA-ACVADKG-FATTFDNRTF
BAG84131_Tetranychus_urticae         ----------------------------------------RLDMLPNSKY
ADU04394.1_B_tabaci_Q                --------------SGFDRLMQDTFQAKYQA-ACVADKM-HATTFDNKTY
Q16927_Aedes_aegypti                 LPAHKPRNRPYYELSNYEQFAVFDRKPQYP--SCSFSND-YFYTFDNKKY
G0ZTK0_Nilaparvata_lugens            --------------NSLE-LMSYNENYDYP--TCSVSKN-SISTFDNKTY
Q9BPS0_Periplaneta_americana         --------------PLLSQVAKTAFPQYYEP-TCVVDYS-KVNTFDNRTY
E0VZ52_Pediculus_humanus_corporis    --------------NYVERYQWSAFQQQYKA-YCTIDKA-QTKTFDNQTY
B0W351_Culex_quinquefasciatus        --------------AWNERLASYAYNGEMNP-SCVVAPK-YVDTFDGRTY
O76823_Blattella_germanica           --------------SFAKRFAKWALPLYYKP-TCVVDSS-YINTFDNFTY
Q7PQM2_Anopheles_gambiae             --------------DYYERMFNYAYRGNYHP-SCAVSNK-FVNTFDGKTY
UPI000258E3A9_Megachile_rotundata    --------------EKEDEADNSWLMDDDVS-TCVMDLN-RAETFDGKEF
ADU04392.1_1_B_tabaci_B              --------------SGFDRLMQDTFQAKYQA-ACVADKM-HATTFDNKTY
Q9U8M0_Periplaneta_americana         --------------TALDRLAREALPLYYEP-TCVLDVS-QAATFDNTTY
ADU04393.1_B_tabaci_ZHJ-II           --------------SGFDRLMQDTFQAKYQA-ACVADKM-HATTFDNRTF
Q27309_Bombyx_mori                   --------------KAYELVANYFTGHQYQP-YCSIDGT-RIHTFSNRSY
```

VWFD

```
A7BK94_Nilaparvata_lugens            PTHADLEGWHVMFASTPKN--FNDNSGRYSASNSQSNSFYKYKK------
Q868N5_Apis_mellifera                PLRL-GPCWHAVMTTYPRINPDNHNEKLHIPKDKS---------------
R4KZ43_Helicoverpa_armigera          DYEL-SKSWHVVMREEYNKIRGKWDD------------------------
Q9U5D8_Plautia_stali                 SIDY-EDFTYVLVYALPGEHFESSEDSSE---EYESTYLGG---------
O02024_Riptortus_clavatus            PIDL-GNCYHVFAMYAPQGQGENRQHESSISNIQQG--------------
D6W7J2_Tribolium_castaneum           PIDL-SKDWVVLLHYVPRRPSPIKNQPYLTVPEQLNQQVEGY-------
D6W721_Tribolium_castaneum           PSSF-SNGWTVLLHYVPRRPSSSQNKPYESVQEQLNELVESY-------
B_tabaci_Asia1                       PAHF-QNNWYVLMAYMNRNNYYNNNFNQYL---QQNKNQHSYRDYNEKRF
```

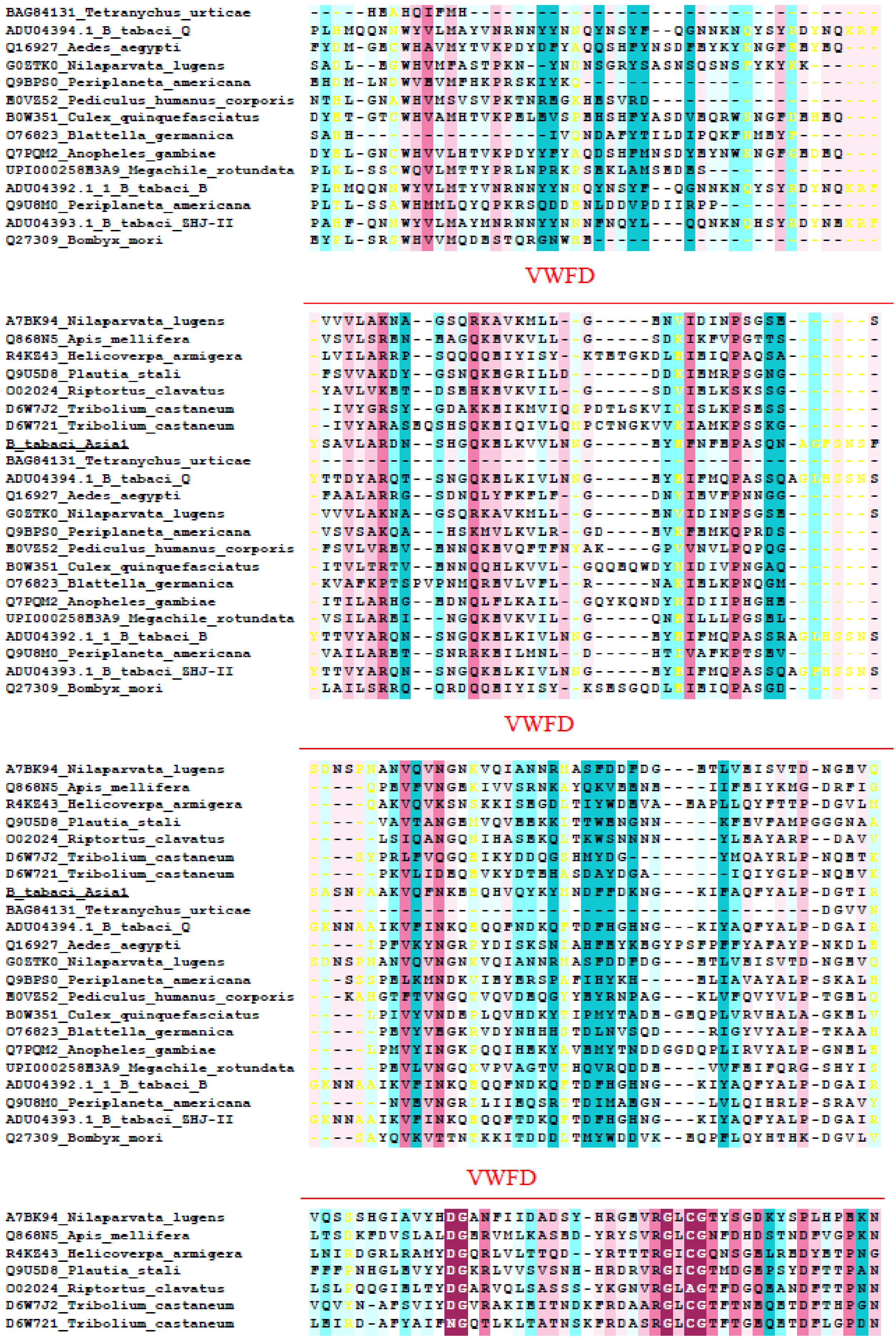
VWFD
VWFD
VWFD

```
B_tabaci_Asia1                       FFAPQAGLEFFYDGARVKFQAASQ-YRGAVRGICGTYSNQYADDFTSPKN
BAG84131_Tetranychus_urticae         VVLPKENVRVAFDGHYLKITTSPL-FVGKTCGLCGNQDQEFYPEFLDPGM
ADU04394.1_B_tabaci_Q                FFAPQSGLQAIYDGARIKIQAANQ-YRGAVRGMCGTYSNQYADTSPPLKT
Q16927_Aedes_aegypti                 VSFFGGKLKFATDGYRARFFSDYS-FYNNFVGLCGTNNGEYFDEFVTPDQ
G0ZTK0_Nilaparvata_lugens            VQSSSHGIAVYHDGANFIIDADSY-HRGEVRGLCGTYSGDKYTDFTTPNK
Q9BPS0_Periplaneta_americana         LDLLNDSLVFVYDGERVMLHAGNH-YRNQVRGLCGTFDGEPSTDFKAPQN
E0VZ52_Pediculus_humanus_corporis    FVSTKYGFNFLYDAQRVQLALSQS-YRNRVGGLCGVYNGEHY-GYVTPQG
B0W351_Culex_quinquefasciatus        VDIRDGQIVIVCDGYRAQILTGQT-FYDNTVGLCGTNNKQEEDDFITPQQ
O76823_Blattella_germanica           IVFPSYEIEMFYDGSRIMIQASNM-YRNFTKGLCGNMDGEFVNDVLTPWG
Q7PQM2_Anopheles_gambiae             ISFRDDDIKIVFDGYRARFFADQS-YFNNFVGLCGTNNGEGEDDFITPDQ
UPI000258E3A9_Megachile_rotundata    VVSEEYDVTLTYDGKRLMIEEPEM-FSYSLRGLCGNYDGDSENDFTSPGN
ADU04392.1_1_B_tabaci_B              FFAPQSGLQAIYDGARIKIQAANQ-YRGAVRGMCGTYSNQYADDFTAPQN
Q9U8M0_Periplaneta_americana         MVAPQHKLLMIHDGKRVLLQASNG-YRDEVRGLCGTFDGEPTTDFTTPSN
ADU04393.1_B_tabaci_ZHJ-II           FFAPQSGLQAIYDGARIKIQAANQ-YRGAVRGMCGTYSNQYADDFTAPQN
Q27309_Bombyx_mori                   INIEDDRIRAIYDGQRFVVFTQD--YRNSTRGICGRMSGEQRDDYLTPEG


A7BK94_Nilaparvata_lugens            KCIMREAIP-FAATYALPGS-SNSNVEQLK---RQADQMTCFRRRH----
Q868N5_Apis_mellifera                -CLFRKPEH-FVASYALISNQCEGDSLNVA---KSLQDHDCIRQERT---
R4KZ43_Helicoverpa_armigera          --LVDQPEH-YGASYSLDVEDSDPKTQGLK----KEAQEKAYQSTP----
Q9U5D8_Plautia_stali                 -CVVKDPQQ-FASSYALPEASLRGPTKPHK---KQGQSGACYPREV----
O02024_Riptortus_clavatus            -RVLRNPLQ-FAATYALLDSECQGPAVQRQ---QQAQQSPSYERRV----
D6W7J2_Tribolium_castaneum           -CIIHNPEV-FVATNTVPEKQRTKRMNEMMM--IQAEEKKCYRKSV----
D6W721_Tribolium_castaneum           -CIIHAPEK-FIESYTIGQQLMQRGSKMRR----SDNEKQCYFKKV----
B_tabaci_Asia1                       -CVMRNPEY-FTAAYAFIDSSSPAQLKAQR---DQAEQSSCAYKTY----
BAG84131_Tetranychus_urticae         -VNLASNLTAFRDSYSL---------------------HNCTDPNP----
ADU04394.1_B_tabaci_Q                -VSTRTQKD-FAASYAVIDSSSPSQVKSQK---ERAQQNFCARKNN----
Q16927_Aedes_aegypti                 -CYMRKPEF-FAASYAITGQNCTGPAKAFNYAYQQKAKQECVKREV----
G0ZTK0_Nilaparvata_lugens            -CIMREARL-FAATYALPGS-SNSNVEQLK---RQADQMTCFRRRH----
Q9BPS0_Periplaneta_americana         -CHVRDVED-LILAYTLVRDLDRSRLR---------DENICVREDV----
E0VZ52_Pediculus_humanus_corporis    -YILNSPEE-FVATWAVT---TEGHVAELK---KKAQEHSVYKKTV----
B0W351_Culex_quinquefasciatus        -CVMRKPEY-FAASWAVTGQNCTGPAKAFAIASQQKQKEACLKVEY----
O76823_Blattella_germanica           -CYAKDMAL-FVASYA---DNSNSEVRKIK---ATQNEQTCVP-------
Q7PQM2_Anopheles_gambiae             -CVMRKPEY-FAASYALTGMNCSGPAQAYFTEYHQKAQQHCVKPQY----
UPI000258E3A9_Megachile_rotundata    -CLLTKPEE-FIASYTLTKEQCEG----------ESLQKVKSLQK-----
ADU04392.1_1_B_tabaci_B              -CVYKNPED-FAAVYAVIDSSSPSQVKSQK---ERAQQNFCAHKNN----
Q9U8M0_Periplaneta_americana         -CILNDPKA-FINSYRLGGDREDNAWM------LNYRAQPCVSRNFTSTD
ADU04393.1_B_tabaci_ZHJ-II           -CVYKNPED-FAAVYAVVDSSSPSQVKSQK---ERAQQNFCARKNN----
Q27309_Bombyx_mori                   --LVDKPEL-YAAAYSLNEENSDPKTQELK----ALATQQAYYPEY----


A7BK94_Nilaparvata_lugens            IFANVITSNDYDRS------------------------------------
Q868N5_Apis_mellifera                QQRNVISDSESGRL------------------------------------
R4KZ43_Helicoverpa_armigera          KYTAILRSDEQWLQ------------------------------------
Q9U5D8_Plautia_stali                 VFADVVSDTDAGRG------------------------------------
O02024_Riptortus_clavatus            ILGDVVNELEAGRQ------------------------------------
D6W7J2_Tribolium_castaneum           QFVKIVSAQDSGIM------------------------------------
D6W721_Tribolium_castaneum           YYANYISNQDAGRV------------------------------------
B_tabaci_Asia1                       LAGNYVSRNEGQNGNKYYKYNNNDKYYESAYKNSKYYDAARYNHQYNPYY
BAG84131_Tetranychus_urticae         CHDDVCDVDAMAHP------------------------------------
ADU04394.1_B_tabaci_Q                QFGNYVSRSDAGYG---YKYNNNDKYYESAYKNTKYYDPS--NSQYNKYY
Q16927_Aedes_aegypti                 YYGDIIYNQEYYHP------------------------------------
G0ZTK0_Nilaparvata_lugens            IFANVITSNDYDRS------------------------------------
Q9BPS0_Periplaneta_americana         QLVNLTNHRHAEKS------------------------------------
E0VZ52_Pediculus_humanus_corporis    LYTNVVSTPDSRYH------------------------------------
B0W351_Culex_quinquefasciatus        MYGNVVSDVEAGRK------------------------------------
O76823_Blattella_germanica           QFHQPLVSHQMRLS------------------------------------
Q7PQM2_Anopheles_gambiae             YFGNVISEQEAGRQ------------------------------------
UPI000258E3A9_Megachile_rotundata    --------------------------------------------------
ADU04392.1_1_B_tabaci_B              QFGNYVSRSDAGYG---YKYNNNDKYYESAYKNTKYYDPS--NSQYNKYY
Q9U8M0_Periplaneta_americana         IIGKHMPRNPASRG------------------------------------
ADU04393.1_B_tabaci_ZHJ-II           QFGNYVSRSDAGYG---YKYNNNDKYYESAYKNTKYYDPS--NSQYNKYY
Q27309_Bombyx_mori                   KYTSILRSDPTWQE------------------------------------


A7BK94_Nilaparvata_lugens            --------------------------------------------------
Q868N5_Apis_mellifera                --------------------------------------------------
R4KZ43_Helicoverpa_armigera          --------------------------------------------------
Q9U5D8_Plautia_stali                 --------------------------------------------------
O02024_Riptortus_clavatus            --------------------------------------------------
D6W7J2_Tribolium_castaneum           --------------------------------------------------
```

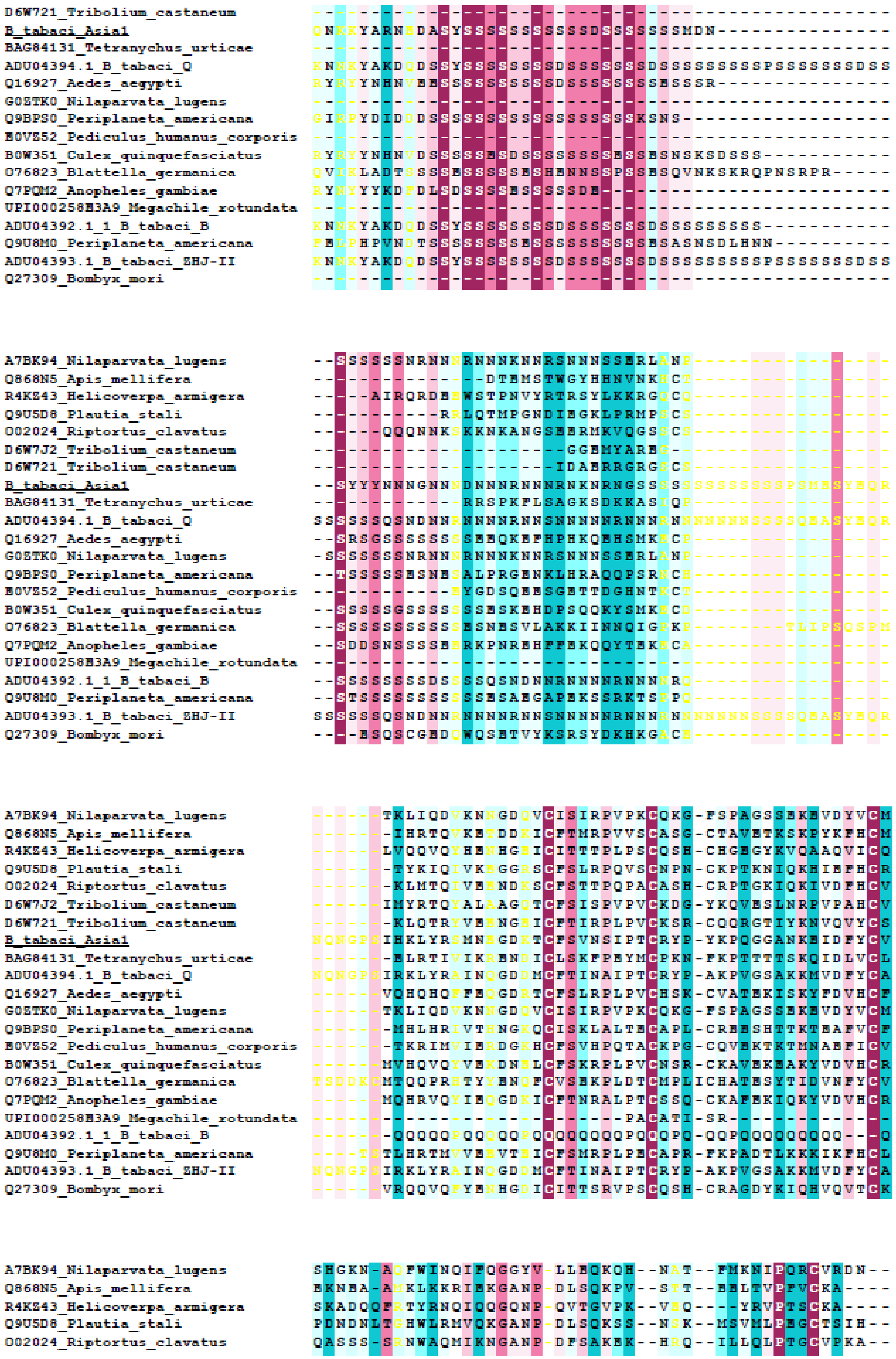

```
D6W7J2_Tribolium_castaneum          ESSQL-ARSLRRQIDQGANP-SFKSKPQ--HRE--VMMQMAKRCEA----
D6W721_Tribolium_castaneum          RSTSS-TNLWKNEIQKGASP-DFSLQQV--SKT--IQMEMPKECLP----
B_tabaci_Asia1                      PRNSEEAQYFEKLMKKGVNPSQLSSKKA--NNQ--FKVNIPEYCVA----
BAG84131_Tetranychus_urticae        PNAHPTAHLWAKEAEKRLIT-EVRDFDS--TET--STYDRPTKCVAESVR
ADU04394.1_B_tabaci_Q               PKSSSEAQHFSKLIAKGAAPSQLSLKKP--NQK--FEVNIPEYCVA----
Q16927_Aedes_aegypti                EKDSTQAKKYKSDIGRGYTP-DFKSFAP--HKT--YKFNYPKSCVYKAY-
G0ZTK0_Nilaparvata_lugens           SHGKN-AQFWINQIFQGGYV-KLEQKQH--NAT--FMKNIPQRCVRDN--
Q9BPS0_Periplaneta_americana        PPGPT-ADHYTKLVRKGVSP-DFSRKTD--IVN--LRVTIPSRCVSKI--
E0VZ52_Pediculus_humanus_corporis   NKTQT-SSHWAQMVARGAQP-NFKNKAN--AQK--MQFEGPEVCTDA---
B0W351_Culex_quinquefasciatus       SVQDSAAQMLKQQIRKGVNP-DMSAKSV--SKT--MKFAVPKQCVHIH--
O76823_Blattella_germanica          PLGPA-ANHYMKLVKKGILP-DLSRNRN--GKRVVLPVEIPIQCEPVLN-
Q7PQM2_Anopheles_gambiae            DATDSAAQLFKQQIRKGVNP-DMSNKSV--TKT--VKYFFPKKCVFAN--
UPI000258E3A9_Megachile_rotundata   --------------------------------R-----------------
ADU04392.1_1_B_tabaci_B             PRSQLRTEKPKRTLYPQTVPCHQPRRRHVLHHQRYPNLQIPSQARWKRQE
Q9U8M0_Periplaneta_americana        TKGPT-ASHWLKMVKKGVNP-DFSKKRE--HKQ--LEVDIPAKCVRH---
ADU04393.1_B_tabaci_ZHJ-II          PKSSSEAQHFSKLIAKGAAPSQLSLKKP--NQK--FEVNIPEYCVA----
Q27309_Bombyx_mori                  SKLDHDFRMYKEQIKKGQNP-EVSGIPS--VKQ----FKVPVTCQP----


A7BK94_Nilaparvata_lugens           --
Q868N5_Apis_mellifera               --
R4KZ43_Helicoverpa_armigera         --
Q9U5D8_Plautia_stali                --
O02024_Riptortus_clavatus           --
D6W7J2_Tribolium_castaneum          --
D6W721_Tribolium_castaneum          --
B_tabaci_Asia1                      --
BAG84131_Tetranychus_urticae        GI
ADU04394.1_B_tabaci_Q               --
Q16927_Aedes_aegypti                --
G0ZTK0_Nilaparvata_lugens           --
Q9BPS0_Periplaneta_americana        --
E0VZ52_Pediculus_humanus_corporis   --
B0W351_Culex_quinquefasciatus       --
O76823_Blattella_germanica          --
Q7PQM2_Anopheles_gambiae            --
UPI000258E3A9_Megachile_rotundata   --
ADU04392.1_1_B_tabaci_B             NG
Q9U8M0_Periplaneta_americana        --
ADU04393.1_B_tabaci_ZHJ-II          --
Q27309_Bombyx_mori                  --
```
