# Supplementary material for: Molecular Characterization of Vitellogenin and Vitellogenin Receptor of Bemisia tabaci
Source: PLoS One. 2016 May 9;11(5):e0155306. doi: 10.1371/journal.pone.0155306 (PMC4861306; doi:10.1371/journal.pone.0155306)
Supplement: S5 File — (DOC) [file pone.0155306.s009.doc]

**Supplementary file 5.** Nucleotide and protein sequence of vitellogenin receptor of *Bemisia tabaci* Asia 1.

1 atgattcagagggaatggagctccatatccaagggctcatggtgc

M I Q R E W S S I S K G S W C

46 acagcattattagtagttatcgcagtcttttgcacatttgtacaa

T A L L V V I A V F C T F V Q

91 tcatcttcatcatatgaatgtgtcggaccatcccatttcgaatgt

S S S S Y E C V G P S H F E C

136 acaaatcacaggtgtatatccatggatttaaggtgcgatggagat

T N H R C I S M D L R C D G D

181 gacgattgtaatgatggatccgatgaacacggttgtaatgtggat

D D C N D G S D E H G C N V D

226 aaatcgaaaaatgaaacctgtgctagtacccagtttgattgcggt

K S K N E T C A S T Q F D C G

271 cagggccaatgtataccacggtcctgggtttgtgatgggaatgca

Q G Q C I P R S W V C D G N A

316 gattgtgaggacggtaaagatgaaggagctgcaggctgcgctgaa

D C E D G K D E G A A G C A E

361 agccattgcgcagcgtctgagtgggagtgccctcataaccatcgc

S H C A A S E W E C P H N H R

406 tgtatcccgaatgattatatctgtgatggggatgatgattgtggt

C I P N D Y I C D G D D D C G

451 gacaattcagatgaaaacgactgtacaggcaagaataattttaca

D N S D E N D C T G K N N F T

496 gagtgcacctcagcgtttgggaaatttctctgcaaaaataggaat

E C T S A F G K F L C K N R N

541 cagtgcatcgatgatactcttctgtgcaacgggcaccctgactgt

Q C I D D T L L C N G H P D C

586 aaagacgggtcagatgagggtggtcactgtgcttcgaaagctcaa

K D G S D E G G H C A S K A Q

631 gtagctgctgactgtgcaaagctaaactgtacccactcgtgtgtt

V A A D C A K L N C T H S C V

676 gaaagcccagatggaccagtttgcgtctgtggatcaggatatcac

E S P D G P V C V C G S G Y H

721 cttgaaggaaacgtctgtgaagatattaacgagtgcttggaatgg

L E G N V C E D I N E C L E W

766 ggaacatgtgaccaaatgtgtgaaaacacggtcggaggttacatc

G T C D Q M C E N T V G G Y I

811 tgtgaatgtgaacctggatacaaactggaaagtaacggacgcaca

C E C E P G Y K L E S N G R T

856 tgcaaagcagaagagggtgaaggacttctaatctactcaagcctc

C K A E E G E G L L I Y S S L

901 aaaaagattaagtctctatatctgacatctcgcatatcaatgaca

K K I K S L Y L T S R I S M T

946 gttgcttctgaggttccatatgcgacgggtgtgtcttttgatggt

V A S E V P Y A T G V S F D G

991 caacatgtctattggacgaccgtgctcgatggagtcgaatctatt

Q H V Y W T T V L D G V E S I

1036 gttcgggcaagtgaggatggatctcatgagaccaccattgttgat

V R A S E D G S H E T T I V D

1081 tcaggtgttggctcccctgaagacctagctgtcgactgggtgact

S G V G S P E D L A V D W V T

1126 ggtaacatctacttcactgatggcgagtaccaacagattggcatt

G N I Y F T D G E Y Q Q I G I

1171 tgcacctacaacgaagagctggttgaaacaaaatgcgctgtcctc

C T Y N E E L V E T K C A V L

1216 cacaacaaagacctgaacaagccccgcgcaattgttttaaaccca

H N K D L N K P R A I V L N P

1261 gctgatgcggtcatgtactggtctgattggggctttaagccactc

A D A V M Y W S D W G F K P L

1306 atcgcccgttctggaatggatggctcagacttctatgagtttgta

I A R S G M D G S D F Y E F V

1351 acgacagagctccattggcccaatggtctcacaattgatcacgga

T T E L H W P N G L T I D H G

1396 aatcggagagtatattgggtcgatgcgagacttggaactgttgaa

N R R V Y W V D A R L G T V E

1441 actgttgattttcaaggccgtgatcggcggaaaatattaactgat

T V D F Q G R D R R K I L T D

1486 ctcaatgatcatccttttgcaattgctgtttttgaagataaaatc

L N D H P F A I A V F E D K I

1531 tactggagtggatggacaaaccaagaaatagtagaatgtaataag

Y W S G W T N Q E I V E C N K

1576 ttcaccgggaaaaaccgagtacaagtcgtcaaaagtcggaaagac

F T G K N R V Q V V K S R K D

1621 aaaatttacggtgtgcacatttttcacccaactttgcaaaatcat

K I Y G V H I F H P T L Q N H

1666 tcgctaccgaatccctgtgccgggaagtgcagtgacatctgtgct

S L P N P C A G K C S D I C A

1711 ctatctccatcagcttcaagcggaggcaaaggctactcttgcttg

L S P S A S S G G K G Y S C L

1756 tgccctgacaacaaaatcttgtccccctctggagagtggtgccaa

C P D N K I L S P S G E W C Q

1801 gaacagcccaaagaatctgtcattgttagcattggaaactttgtt

E Q P K E S V I V S I G N F V

1846 ttccagctgaaagtgactcttgggaagcagtacattcatccgctg

F Q L K V T L G K Q Y I H P L

1891 cctgttaataacctccagtctgtcagtgcaattgtctacaactcc

P V N N L Q S V S A I V Y N S

1936 ttcgatggctctctattaatagctgacccagatgccaaaatgatt

F D G S L L I A D P D A K M I

1981 tactcgtatcaactgaacacggacaccatggagactttaattgat

Y S Y Q L N T D T M E T L I D

2026 ctcaaagtgggctatgtctcagcattggcttacgatcccattggc

L K V G Y V S A L A Y D P I G

2071 cggaatctctattggtgcgacaaagaggctggcactgtagaagtg

R N L Y W C D K E A G T V E V

2116 ttcagtttcttctctcacagacggaagttgttgctccgagaattt

F S F F S H R R K L L L R E F

2161 gacgatgaaaaacctttcgccatgactctcataccagaagaaggc

D D E K P F A M T L I P E E G

2206 ctgatgttcgtcattgccaaagcccatgaccaccttcatattgat

L M F V I A K A H D H L H I D

2251 cggatcaacatggatggctcactaagcacactgactcacatgacc

R I N M D G S L S T L T H M T

2296 agtctcaagttgcaaggaccggatgtagctttgcactatgacagt

S L K L Q G P D V A L H Y D S

2341 gattctcgcagggtttattgggctgatcactcagctggcctcatt

D S R R V Y W A D H S A G L I

2386 gaaagcaccgatacgaatggaaacgacaggcaagtataccgtgac

E S T D T N G N D R Q V Y R D

2431 gtatcatcacctttagctctgaccgatgtggatagagacctttat

V S S P L A L T D V D R D L Y

2476 tggacatcagatggtcgcccacacctgtactactctgagaaagcc

W T S D G R P H L Y Y S E K A

2521 aatgcaagcatgcccgtcaggaaaatcaacatggaaagatttttg

N A S M P V R K I N M E R F L

2566 cgatcaccaaaggaccactatcggatgtttgttacggcaatcatc

R S P K D H Y R M F V T A I I

2611 cctgataaaactactagagatcatccttgccaaactaacaatggc

P D K T T R D H P C Q T N N G

2656 aaatgtagccatttttgcctcctcaccagtcgtaatccaaagcat

K C S H F C L L T S R N P K H

2701 gtatgcagttgtcctgatgggatgaagctggcggataatggtcag

V C S C P D G M K L A D N G Q

2746 gattgtgaagagattgctgcatgtggagcacatgaatatcattgc

D C E E I A A C G A H E Y H C

2791 acgacaggcgaatgcattccaatgtcgaagaaatgcgaccgaaac

T T G E C I P M S K K C D R N

2836 aaggattgtccctatggcgaagatgagaccttctgcccagcacag

K D C P Y G E D E T F C P A Q

2881 tgtgagactgatcagtttgcgtgctttgatggacaaaaatgtatc

C E T D Q F A C F D G Q K C I

2926 gatgccaaggatagatgcaacatgcattttgattgtcacgaccac

D A K D R C N M H F D C H D H

2971 tctgatgaggctaattgccaaaatgtcacctgcgatcaatcatac

S D E A N C Q N V T C D Q S Y

3016 aactttttgtgccgaaccggtgaatgcgtaagtcatgctgtgctg

N F L C R T G E C V S H A V L

3061 tgcaataatgaatggaattgcaaggatggaagtgatgaagagaac

C N N E W N C K D G S D E E N

3106 tgtacgacttccacgtgcccttccaacgagtttcggtgtcattcc

C T T S T C P S N E F R C H S

3151 ggaacatgcatcccgaagaactgggtctgtgatctggatgccgat

G T C I P K N W V C D L D A D

3196 tgccccgaccaatcagatgaaaataactgcagtttctcaagaaaa

C P D Q S D E N N C S F S R K

3241 gaaaagtgcaccgaattcttgtgtcagagtggcatgtgtgtagca

E K C T E F L C Q S G M C V A

3286 caggaacttgtctgtaacggtcaaaccgaatgtgacgatggaagt

Q E L V C N G Q T E C D D G S

3331 gacgaattcaattgcgatgagccggtcccaaaaacggcaaacaaa

D E F N C D E P V P K T A N K

3376 gaggatggttttatcgataactgtgatgaagagaaggagttcatg

E D G F I D N C D E E K E F M

3421 tgcgagcctggcaaatgcattaatcttatattcaagtgtaatggt

C E P G K C I N L I F K C N G

3466 gctaaagattgcgagaacggagctgatgagctaaactgcataggc

A K D C E N G A D E L N C I G

3511 tgtgaacaattcacttgcaataatgggaaatgtatcacttatgat

C E Q F T C N N G K C I T Y D

3556 cttgtatgcaatgatgatgatgactgtggtgactcatccgatgaa

L V C N D D D D C G D S S D E

3601 agaccactcaactcgtgcccagacagcaaagaaaacccagctatt

R P L N S C P D S K E N P A I

3646 gtgcctgctcatattcccaatgtgtgccatggatttgtttgcaag

V P A H I P N V C H G F V C K

3691 aacggtgaatgccttgatgatttcagtctagtctgcaacaaaaag

N G E C L D D F S L V C N K K

3736 caagactgtaaggatggctcagatgaaggtggacgatgtggttca

Q D C K D G S D E G G R C G S

3781 agctgcgatgtgacagcaaattgcagtcagatttgtcgtgataag

S C D V T A N C S Q I C R D K

3826 ccaaatggacatgagtgtgcgtgtgtccctggctttaaaattgct

P N G H E C A C V P G F K I A

3871 gaggacggtcgcgattgcgaggatattgatgagtgcaccgaattg

E D G R D C E D I D E C T E L

3916 gagccgtgtagccagatgtgtttcaacacctatggaagttacaca

E P C S Q M C F N T Y G S Y T

3961 tgtgcctgtcttggtccagattacattaaaaagtcagatgggtcc

C A C L G P D Y I K K S D G S

4006 tgcaaagccactggcccaaaattgcagtacgtctttgctaccggt

C K A T G P K L Q Y V F A T G

4051 taccagattcgcactatatcatacctaatgaccgatgtcaaagtg

Y Q I R T I S Y L M T D V K V

4096 gcatactatagtgcagatcttgaagtttcaggatttgatgtgaac

A Y Y S A D L E V S G F D V N

4141 atgagaacagagcatgtctactggtcatctgaaaacaagggtgtc

M R T E H V Y W S S E N K G V

4186 atcacaaaaatgtcattaacgcacagacacgagccaaaacatttc

I T K M S L T H R H E P K H F

4231 atcactggtttacgccgtccatcagagctggcagtcgattggata

I T G L R R P S E L A V D W I

4276 acacacaacctctactttgtccaagcaaggaacaccattaacgtg

T H N L Y F V Q A R N T I N V

4321 tgtaacttccaccttgaacgctgcgcacaaattttgaccgctgaa

C N F H L E R C A Q I L T A E

4366 agtggcttggagatcaacagtcttgccgttgatcctgtcagaggt

S G L E I N S L A V D P V R G

4411 gtccttttctggagtgaaacaagtcgcatcgtctggaatatgcct

V L F W S E T S R I V W N M P

4456 aagagcaccatcagacgcgcagacatgaacggaaaaaatattgaa

K S T I R R A D M N G K N I E

4501 accattgtttcagccaatgtgagctacgcattggatcttgctcta

T I V S A N V S Y A L D L A L

4546 gatccaatcctcaatcatgtctattgggtcgacaaaacattgaag

D P I L N H V Y W V D K T L K

4591 gtcattgaaagagccaactatgatggaactcgtcgacgtgtcatc

V I E R A N Y D G T R R R V I

4636 ttgacatcaaagttccatccaaaatctgttgcgctctttgatggt

L T S K F H P K S V A L F D G

4681 tctatctactggtcagttgagtcgagtggatccccgattacgaag

S I Y W S V E S S G S P I T K

4726 tgtgctcttcaaggactttccacggaatcgtactcgtgcaatcaa

C A L Q G L S T E S Y S C N Q

4771 atcccaatcaaagttgtggatccaataactcacttcactctcatg

I P I K V V D P I T H F T L M

4816 caaccagctctgcaaagaaacatatcaaacgcttgtaggaacatg

Q P A L Q R N I S N A C R N M

4861 gagtgcagtcacatgtgcgttctcagctcgacattaccatcatgc

E C S H M C V L S S T L P S C

4906 atttgtcgcaatggaaagatagttcctccaaaaacagcctgtacg

I C R N G K I V P P K T A C T

4951 gatagcaattatatgccagaaacccactttttggaaacaactgga

D S N Y M P E T H F L E T T G

4996 accgttgatggtcaaagtcctggatattcttggtcatcaatctgt

T V D G Q S P G Y S W S S I C

5041 gcaacaatcatcttagtcgctttcatcggaacaactttctatgcc

A T I I L V A F I G T T F Y A

5086 ttattctactactataactccaagtacaacatgcgacgtctattt

L F Y Y Y N S K Y N M R R L F

5131 ccatcaattcattttaaaaacccagccttcaacctacagtcaaaa

P S I H F K N P A F N L Q S K

5176 ttccaggctaacgggatgaccggtttagccagtggcaaccacatg

F Q A N G M T G L A S G N H M

5221 gctcacttgtcatcgaaggaccatcatttcgaaaaccctctgcaa

A H L S S K D H H F E N P L Q

5266 gagagtcgtgaaggtgaagtgagaatcgttactccaaatgaaatc

E S R E G E V R I V T P N E I

5311 acgattagcagagccgagaccagttggacatcagcccatctagaa

T I S R A E T S W T S A H L E

5356 gactcaagctctatagaaacagaatacgcagaccttgttgtcgag

D S S S I E T E Y A D L V V E

5401 acaaaccccaaagctaatttgatatcgtga 5430

T N P K A N L I S *
