# Supplementary material for: Molecular Characterization of Vitellogenin and Vitellogenin Receptor of Bemisia tabaci
Source: PLoS One. 2016 May 9;11(5):e0155306. doi: 10.1371/journal.pone.0155306 (PMC4861306; doi:10.1371/journal.pone.0155306)
Supplement: S6 File — Orange, green, red, blue and purple colour fonts show LDLa, EGF, calcium binding EGF, LDLb and O-Linked sugar domains, respectively. N-terminus signal peptide is bold and underlined. Transmembrane and cytoplasmic regions are highlighted in gray and yellow colours, respectively. (DOC) [file pone.0155306.s010.doc]

**Supplementary file 6.** Protein sequence whitefly (*Bemisia tabaci* Asia 1) vitellogenin receptor with colour demarcation of important domains. Orange, green, red, blue and purple colour fonts show LDLa, EGF, calcium binding EGF, LDLb and O-Linked sugar domains, respectively. N-terminus signal peptide is bold and underlined. Transmembrane and cytoplasmic regions are highlighted in gray and yellow colours, respectively.

**MIQREWSSISKGSWCTALLVVIAVFCTFVQSSSS**Y**ECVGPSHFECTNHRCISMDLRCDGDDDCNDGSDEHGCNV**DKSKNE**TCASTQFDCGQGQCIPRSWVCDGNADCEDGKDEGAAGCAE**S**HCAASEWECPHNHRCIPNDYICDGDDDCGDNSDENDCTG**KNNFT**ECTSAFGKFLCKNRNQCIDDTLLCNGHPDCKDGSDEGGHCAS**KAQVAA**DCAKLNCTHSCVESPDGPVCVCGSGYHLEGNVCEDINECLEWGTCDQMCENTVGGYICECEPGYKLESNGRTCK**AEEGEGLLIYSSLKKIKSLYLTSRISMTVASEVPYATGVSFDGQHVYWTTVLDGVESIVRASEDGSHE**TTIVDSGVGSPEDLAVDWVTGNIYFTDGEYQQIGICTYNEELV**ETKC**AVLHNKDLNKPRAIVLNPADAVMYWSDWGFKPLIARSGMDGSDFYEFVTTELHWPNGLTIDHGNRRVYWVDARLGTVETVDFQGRDRRKILTDLNDHPFAIAVFEDKIYWSGWTNQEIVECNKFTGKNR**VQVVKSRKDKIYGVHIFHPTLQNHSLPN**PCAGKCSDICALSPSASSGGKGYSCLCPDNKILSPSGEWCQ**EQPKESVIVSIGNFVFQLKVTLGKQYI**HPLPVNNLQSVSAIVYNSFDGSLLIADPDAKMIYSYQLNTDTMETLIDLKVGYVSALAYDPIGRNLYWCDKEAGTVEVFSFFSHRR**KLLLREFDDEKPFAMTLIPEEGLMFVIAKAHDHLHIDRINMDGSLST**LTHMTSLKLQGPDVALHYDSDSRRVYWADHSAGLIESTDTNGNDR**QVYRDVSSPLALTDVDRDLYWTSDGRPHLYYSEKANASMPVRKINMERFLRSPKDHYRMFVTAIIPDKTTRDH**PCQTNNGKCSHFCLLTSRNPKHVCSCPDGMKLADNGQDCE**EIA**ACGAHEYHCTTGECIPMSKKCDRNKDCPYGEDETFCPAQCETDQFACFDGQKCIDAKDRCNMHFDCHDHSDEANCQN**V**TCDQSYNFLCRTGECVSHAVLCNNEWNCKDGSDEENCTT**STCPSNEFRCHSGTCIPKNWVCDLDADCPDQSDENNCSFSRKE**KCTEFLCQSGMCVAQELVCNGQTECDDGSDEFNCDE**PVPKTANKEDGFIDNCDEEKEFMCEPGKCINLIFKCNGAKDCENGADELNCIGCEQFTCNNGKCITYDLVCNDDDDCGDSSDERPLNSCPDSKENPAIVPAHIPN**VCHGFVCKNGECLDDFSLVCNKKQDCKDGSDEGGRCGSSCDVTANCSQICRDKPNGHECACVPGFKIAEDGRDCEDIDECTELEPCSQMCFNTYGSYTCACLGPDYIKKSDGSCK**ATGPKLQYVFATGYQIRTISYLMTDVKVAYYSADLEVSGFDVNMRTEHVYWSSENKGVITKMSLTHRHE**PKHFITGLRRPSELAVDWITHNLYFVQARNTINVCNFHLERC**A**QILTAESGLEINSLAVDPVRGVLFWSETSRIVWNMPKSTIRRADMNGKNIETIVSANVSYALDLALDPILNHVYWVDKTLKVIERANYDGTRR**RVILTSKFHPKSVALFDGSIYWSVESSGSPITKCALQGLSTESYSCNQIPIKVVDPITHFTLMQPALQRNISN**ACRNMECSHMCVLSSTLPSCICRNGKIVPPKTACTDSNYMPETHFLETTGTVDGQSPGYSWSSICATIILVAFIGTTFYALFYYY**NSKYNMRRLFPSIHFKNPAFNLQSKFQANGMTGLASGNHMAHLSSKDHHFENPLQESREGEVRIVTPNEITISRAETSWTSAHLEDSSSIETEYADLVVETNPKANLIS
