# Supplementary material for: Molecular Characterization of Vitellogenin and Vitellogenin Receptor of Bemisia tabaci
Source: PLoS One. 2016 May 9;11(5):e0155306. doi: 10.1371/journal.pone.0155306 (PMC4861306; doi:10.1371/journal.pone.0155306)
Supplement: S7 File — (DOC) [file pone.0155306.s011.doc]

**Supplementary file 7:** ConSurf blast result of whitefly (*Bemisia tabaci* Asia 1) vitellogenin receptor.

**The conservation scale:**

| 1  2  3  4  5  6  7  8  9   | **Variable** | **Average** | **Conserved** | | --- | --- | --- | |
| --- | --- | --- | --- |

**e - An exposed residue according to the neural-network algorithm.**
**b - A buried residue according to the neural-network algorithm.**
**f - A predicted functional residue (highly conserved and exposed).**
**s - A predicted structural residue (highly conserved and buried).**
**X - Insufficient data - the calculation for this site was performed on less than 10% of the sequences.**

1          11         21         31         41         
**MIQREWSSIS KGSWCTALLV VIAVFCTFVQ SSSSYECVGP SHFECTNHRC**
**eeeeeeeebb eeebeebebb bbbbebeeee eeeeeebeee eebebeeeeb**
                                       **s**  **f**  **s** **s** **f**  **s** 51         61         71         81         91         
**ISMDLRCDGD DDCNDGSDEH GCNVDKSKNE TCASTQFDCG QGQCIPRSWV**
**eeeebebeee eebeeeeeee ebeeeeeeee ebeeeebebe eeebbeeebe**
**f**     **sf**    **fs** **f** **fff**   **s**                 **s**     **ss**      101        111        121        131        141        
**CDGNADCEDG KDEGAAGCAE SHCAASEWEC PHNHRCIPND YICDGDDDCG**
**beeeeebeee eeeeeeebee eeebeeeeeb eeeeebbeee bebeeeeeee**
**sf**   **fs** **f**  **fff**    **s**            **s**  **f** **fs**       **sff**  **ff**

151        161        171        181        191        
**DNSDENDCTG KNNFTECTSA FGKFLCKNRN QCIDDTLLCN GHPDCKDGSD**
**eeeeeeebeb eeeeeebeee eeebbbeeee ebbebeebbe eeeebeeeee**
**f** **fff**  **s**         **s**         **s** **f**    **s**      **s**      **s** **f** **ff**

201        211        221        231        241        
**EGGHCASKAQ VAADCAKLNC THSCVESPDG PVCVCGSGYH LEGNVCEDIN**
**eeeebeeeee beeebeeeeb eeebeeeeee ebbbbeeeee eeeeebeebe**
**f**              **s**         **s**   **f** **f**  **s** **s**  **f**      **f** **s** **fs**

251        261        271        281        291        
**ECLEWGTCDQ MCENTVGGYI CECEPGYKLE SNGRTCKAEE GEGLLIYSSL**
**ebeeebbbee ebeeeeeeee beeeeeeebe eeeeebeeee eebbbbbbbb**
**fs**   **s** **sff** **s** **f**  **f**    **s** **f**  **ff**     **f**   **s** **f** **f**       **s**

301        311        321        331        341        
**KKIKSLYLTS RISMTVASEV PYATGVSFDG QHVYWTTVLD GVESIVRASE**
**eebebbbbee ebbbbbbeeb eebbbbebee ebbbbbebbe beebbbebee**
  **sf**     **f**               **s**         **s** **s**         **ssf**

351        361        371        381        391        
**DGSHETTIVD SGVGSPEDLA VDWVTGNIYF TDGEYQQIGI CTYNEELVET**
**eeeeeebbbb bbbeeeeebb bbbbbbebbb beeeeeebbb beeeeebbee**
       **s**    **s**   **fff**   **ss**  **s** **fsss sf**     **sss s**  **f**

401        411        421        431        441        
**KCAVLHNKDL NKPRAIVLNP ADAVMYWSDW GFKPLIARSG MDGSDFYEFV**
**bbebbeeeee eebebbbbbb bebbbbbbee eeebebbebb beeeebeebb**
 **s** **s**  **f**  **f** **fsf**  **s**  **s**    **s**   **ff f**    **ss**  **s sfff**     **s**

451        461        471        481        491        
**TTELHWPNGL TIDHGNRRVY WVDARLGTVE TVDFQGRDRR KILTDLNDHP**
**eeebebeebb bbbbbeeebb bbebebebbe bbebeeeeee ebbeebbebb**
      **ff**     **s**  **f** **f** **s** **sfsf**    **f s**   **ff** **fff**        **ss**

501        511        521        531        541        
**FAIAVFEDKI YWSGWTNQEI VECNKFTGKN RVQVVKSRKD KIYGVHIFHP**
**bbbbbbeeeb bbbebeeeeb eebeeeeeee eeebbeeeee ebbbbbbbbb**
    **ssff**   **s** **sf**     **s**   **ffffff**                **s**    **s**

551        561        571        581        591        
**TLQNHSLPNP CAGKCSDICA LSPSASSGGK GYSCLCPDNK ILSPSGEWCQ**
**bbbebeeeee beeebeebbb bbeeeeeeee eeebebeeee eeeeeeebbe**
        **ff s**   **sf**  **s**  **s** **f**         **f** **sfsf**  **f** **f**      **s**

601        611        621        631        641        
**EQPKESVIVS IGNFVFQLKV TLGKQYIHPL PVNNLQSVSA IVYNSFDGSL**
**ebeeeeebbb eeebbebeee eeeeeebeeb ebeebeebbb bbbebbeeeb**
   **f**                   **ff**               **s** **s**   **fs**  **f**

651        661        671        681        691        
**LIADPDAKMI YSYQLNTDTM ETLIDLKVGY VSALAYDPIG RNLYWCDKEA**
**bbbeeeeeeb bebebeeeeb eebebbebbe bebbebbebb eebbbbeeee**
   **f**             **f**                     **s**  **s** **f** **s**  **f**

701        711        721        731        741        
**GTVEVFSFFS HRRKLLLREF DDEKPFAMTL IPEEGLMFVI AKAHDHLHID**
**eebebbbbbe eeeebbbeeb eeebeeebbb beeebbbbbb beeeeebbbb**
      **s**                **f**  **f**       **f**    **s**            **s**

751        761        771        781        791        
**RINMDGSLST LTHMTSLKLQ GPDVALHYDS DSRRVYWADH SAGLIESTDT**
**ebbbebebbe ebbbbeeebe beebbbbbee ebeebbbbee eeeebebbee**
**f**  **s** **s**                **sf**   **s**  **f**          **f**      **s**

801        811        821        831        841        
**NGNDRQVYRD VSSPLALTDV DRDLYWTSDG RPHLYYSEKA NASMPVRKIN**
**eeeeeebbee eeeebebbbb bbbbbbbbee eebbbbbeee eeeeebeebe**
 **f**            **f**

851        861        871        881        891        
**MERFLRSPKD HYRMFVTAII PDKTTRDHPC QTNNGKCSHF CLLTSRNPKH**
**beebbebeee eeebbbbbbe ebeeeeeeeb eeeeeebbbb bbbbeeebee**
                             **ffs**   **ff** **sss**  **s**

901        911        921        931        941        
**VCSCPDGMKL ADNGQDCEEI AACGAHEYHC TTGECIPMSK KCDRNKDCPY**
**ebbbeeebeb eeeeeebeee eebeeeebeb eeeebbeeee ebeeeeeeee**
 **s** **sf** **fs** **s**      **s**      **s**      **s**    **ss**      **sf**   **fff**

951        961        971        981        991        
**GEDETFCPAQ CETDQFACFD GQKCIDAKDR CNMHFDCHDH SDEANCQNVT**
**eeeeeebeee beeeebebee eeebbeeeee beeeeebeee eeeeeeeeee**
**ffff**  **s**    **s**    **s** **s** **f**   **ss**      **s**    **fs** **f**  **fff**

1001       1011       1021       1031       1041       
**CDQSYNFLCR TGECVSHAVL CNNEWNCKDG SDEENCTTST CPSNEFRCHS**
**eeeeeebebe eeebbeebee beeeeebeee eeeeebeeee beeeebebee**
        **s**     **s**       **sf**   **fs** **f**  **fff** **fs**     **s**      **s**

1051       1061       1071       1081       1091       
**GTCIPKNWVC DLDADCPDQS DENNCSFSRK EKCTEFLCQS GMCVAQELVC**
**eebbeeebeb eeeeebeeee eeeeeeeeee eeeeebebee eebbeeebeb**
**f** **ss**     **s f**   **fs** **f** **f ff**                **s** **f f** **s**      **s**

1101       1111       1121       1131       1141       
**NGQTECDDGS DEFNCDEPVP KTANKEDGFI DNCDEEKEFM CEPGKCINLI**
**eeeeeeeeee eeeebeeeee eeeeeeeebe eebeeeeebe beeeebbeee**
**f**   **ff** **f** **f ff**  **s**        **f**          **s**    **f**   **s**    **s** **f**

1151       1161       1171       1181       1191       
**FKCNGAKDCE NGADELNCIG CEQFTCNNGK CITYDLVCND DDDCGDSSDE**
**bebeeeeebe eeeeeeebee beebebeeee bbeeebebee eeeeeeeeee**
 **fsf**    **s**     **ff**  **s**      **s** **s** **f**   **ss**     **sf**    **ff** **f** **fff**

1201       1211       1221       1231       1241       
**RPLNSCPDSK ENPAIVPAHI PNVCHGFVCK NGECLDDFSL VCNKKQDCKD**
**eeeeebeeee eeeeeeeeee eeebeeeebe eeebbeebee bbeeeeebee**
     **s**                   **s**    **s**     **s**        **s**     **s** **f**

1251       1261       1271       1281       1291       
**GSDEGGRCGS SCDVTANCSQ ICRDKPNGHE CACVPGFKIA EDGRDCEDID**
**eeeeeeebee ebeeeeebee ebeeeeeeee beeeeebebb eeeeebeebe**
**ffff**   **s**   **fs**     **s**    **s**  **ff**       **f**  **f**      **f**   **s** **fs**

1301       1311       1321       1331       1341       
**ECTELEPCSQ MCFNTYGSYT CACLGPDYIK KSDGSCKATG PKLQYVFATG**
**ebeeeeebee ebeeeeeebe bbbeeeebeb eeeeebeeee eebebbbbbe**
**fs**     **sff** **s** **f**  **ff**   **s** **s**        **f** **f** **fsff** **f**

1351       1361       1371       1381       1391       
**YQIRTISYLM TDVKVAYYSA DLEVSGFDVN MRTEHVYWSS ENKGVITKMS**
**eebeebeeeb eebebbbbbe ebebebbebe beeebbbbbb eeeebbbebe**
  **sf**  **f**                      **f** **f**

1401       1411       1421       1431       1441       
**LTHRHEPKHF ITGLRRPSEL AVDWITHNLY FVQARNTINV CNFHLERCAQ**
**beeeeeeeee bebbeeeeeb bbbbbbebbb bbeeeeebeb bebeeeebbe**
                 **f**      **s**  **s** **s**          **s**    **f**      **s**

1451       1461       1471       1481       1491       
**ILTAESGLEI NSLAVDPVRG VLFWSETSRI VWNMPKSTIR RADMNGKNIE**
**bbebeeeeeb eebebbeebe bbbbbebbeb bbebeebebb ebebeeeebe**
              **f** **sf**      **s**            **f**   **s**       **f** **f**

1501       1511       1521       1531       1541       
**TIVSANVSYA LDLALDPILN HVYWVDKTLK VIERANYDGT RRRVILTSKF**
**ebbbeebebb bebbbbbbee ebbbbeebee ebeebeeeee eeebbbebee**
         **s** **f**   **s**          **f**      **sf**    **ff**   **f**

1551       1561       1571       1581       1591       
**HPKSVALFDG SIYWSVESSG SPITKCALQG LSTESYSCNQ IPIKVVDPIT**
**eeeebbbbee ebbbbbeeeb bebbebebee eeeeebebbe bebebbebbe**
 **f**      **f**           **s**         **f**

1601       1611       1621       1631       1641       
**HFTLMQPALQ RNISNACRNM ECSHMCVLSS TLPSCICRNG KIVPPKTACT**
**bbebbeebee eeeeeebeee ebeebbbbee eeeebbbeee eeeeeeebbe**
   **s** **f**   **f**    **f** **s** **f**       **s**         **s** **s** **ff**        **s**

1651       1661       1671       1681       1691       
**DSNYMPETHF LETTGTVDGQ SPGYSWSSIC ATIILVAFIG TTFYALFYYY**
**eeeeeeebee eeeeeeeeee eeeeeeebbb bbbbbbbbbb bbbbbbbbbe**
 **f**

1701       1711       1721       1731       1741       
**NSKYNMRRLF PSIHFKNPAF NLQSKFQANG MTGLASGNHM AHLSSKDHHF**
**eeeeeeeebe bbeebeeeee ebeeebeeeb eeeeebeeee eebeeeeeee**
               **s** **ff**                      **f**         **f** **f**

1751       1761       1771       1781       1791       
**ENPLQESREG EVRIVTPNEI TISRAETSWT SAHLEDSSSI ETEYADLVVE**
**eeebeeeeeb ebbbebeeee ebeebebebb ebeeeeeeee eeebbebebe**
 **ff**                                              **f**   **f**

1801       
**TNPKANLIS**
**eeeeeeeee**
        **f**
