# Supplementary material for: Molecular Characterization of Vitellogenin and Vitellogenin Receptor of Bemisia tabaci
Source: PLoS One. 2016 May 9;11(5):e0155306. doi: 10.1371/journal.pone.0155306 (PMC4861306; doi:10.1371/journal.pone.0155306)
Supplement: S8 File — Important domains are labeled and highlighted. (PDF) [file pone.0155306.s012.pdf]

Supplementary file 8. Multiple sequence alignment of full length vitellogenin receptor protein sequences of selected insects from each order. Important domains are labeled and highlighted.

1	2	3	4	5	6	7	8	9
	Variable			Average			Conserved	

```
Bemisia_tabaci_Asia_1             M-------------------------------------------------
T1E9W2_Anopheles_aquasalis        R-------------------------------------------------
P98163_Drosophila_melanogaster    MCQAEHQVHPSEQRIRVESPKMTASRRGFNLTSQTRAHPSSG-------G
B4Q2Q9_Drosophila_yakuba          M----------------------TASRRGFNLTSQTRAHTTSGTTSGTTSG
B4IGA9_Drosophila_sechellia       MCQAEHQVPTSEQRIRVKSPKMTASQRGFNLTSQTRAHPASG-------G
K4MUW4_Actias_selene              M-------------------------------------------------
Q1T728_Rhyparobia_maderae         M-------------------------------------------------
O76952_Aedes_aegypti              M-------------------------------------------------
Q6X0I2_Solenopsis_invicta         M-------------------------------------------------
ADE34166.1_Nilaparvata_lugens     M-------------------------------------------------
D5KXW4_Nilaparvata_lugens         M-------------------------------------------------
BAC02725_Periplaneta_americana    M-------------------------------------------------
ADM34986.1_1_B_tabaci_B           M-------------------------------------------------
CAJ19121_Blattella_germanica      M-------------------------------------------------
H2CLV8_Antheraea_pernyi           M-------------------------------------------------
XP_008180459_Acyrthosiphon_pisum  M-------------------------------------------------
UPI000252AABD_Apis_florea         M-------------------------------------------------
UPI0001758603_Tribolium_castaneum M-------------------------------------------------
T1KD20_Tetranychus_urticae        M-------------------------------------------------
UPI0002063B8E_Apis_mellifera      M-------------------------------------------------
E2FLQ4_Bombyx_mori                M-------------------------------------------------

Bemisia_tabaci_Asia_1             --------------------------------------------------
T1E9W2_Anopheles_aquasalis        --------------------------------------------------
P98163_Drosophila_melanogaster    STSSRYGNCQRTHLIINGRHVAISLLLLVGLCGGTAAGTPGSADTR---C
B4Q2Q9_Drosophila_yakuba          SPSSGYENCQRTHLIIHGRHVAISLLVLLGICGATAAGTPGSADTR---C
B4IGA9_Drosophila_sechellia       STSSGYGYWQRTHLIINGRHVAISLLLLLGLCGGTAAGTPGSADTR---C
K4MUW4_Actias_selene              --------------------------------------------------
Q1T728_Rhyparobia_maderae         ------WWSLQAMVIWLSVLWILQPSCNVGAKQN---------------C
O76952_Aedes_aegypti              ------GAIMLRSSAIVLLLIAFGVIINIFGSVDAARKSPSSRIAKAPAC
Q6X0I2_Solenopsis_invicta         --------------------------------------------------
ADE34166.1_Nilaparvata_lugens     -----------------RAIWFLANFVILAAIGASSFETFEGLIPSHDGC
D5KXW4_Nilaparvata_lugens         -----------------RAIWFLANFVILAAIGASSFETFEGLIPSHDGC
BAC02725_Periplaneta_americana    -------------LLYAEIMWIIIQLHTVQGESS---------------C
ADM34986.1_1_B_tabaci_B           --------------------------------------------------
CAJ19121_Blattella_germanica      ------GQLMQAIVIWLGLLTCCINVEVNGYCQG----------------
H2CLV8_Antheraea_pernyi           --------------------------------------------------
XP_008180459_Acyrthosiphon_pisum  -------------WKNSRMLIFFLIFSLVVCCTFSELNEKE--------C
UPI000252AABD_Apis_florea         --------------------------------------------------
UPI0001758603_Tribolium_castaneum --------------------------------------------------
T1KD20_Tetranychus_urticae        --------------------------------------------------
UPI0002063B8E_Apis_mellifera      --------------------------------------------------
E2FLQ4_Bombyx_mori                --------------------------------------------------

Bemisia_tabaci_Asia_1             ------IQREWSSISKGSWCTALLVV--IA-VFCTFVQSSSSYECVGPSH
T1E9W2_Anopheles_aquasalis        --------------------------------------------CASP--
P98163_Drosophila_melanogaster    DAGQFQCR-DGGCILQAKMCDGRGDC--KD-SSDELDCD--YRLCRPPHW
B4Q2Q9_Drosophila_yakuba          DAGQFQCR-DGGCILQAKMCDGRGDC--KD-GSDELDCD--YRLCRPPHW
B4IGA9_Drosophila_sechellia       DAGQFQCR-GGGCILQAKMCDGRGDC--KD-SSDELDCD--YRLCQPPHW
K4MUW4_Actias_selene              ------------KVVLLAIVLCATSC--AGQFVDEMQVY--EKECLGEDV
Q1T728_Rhyparobia_maderae         PENAFKCHSDGTCISGYHHCDGHVDC--ND-GSDEINCEHAQSQCLEPRW
O76952_Aedes_aegypti              AENEYRCD-NGACIPDVNHCNGAKDC--TD-GSDEVGCD--YFLCKKPMW
Q6X0I2_Solenopsis_invicta         -----------RFIVLLFICSFIYPCYVSSIGFRRISKVSLKKTKCEDGY
ADE34166.1_Nilaparvata_lugens     PLDHFMCD-DGECILLAQHCDEKDDC--SD-RSDEKNCE--LDFCRGPQF
D5KXW4_Nilaparvata_lugens         PLDHFMCD-DGECILLAQHCDEKDDC--SD-RSDEKNCE--LDFCRGPQF
BAC02725_Periplaneta_americana    PSGFFTCH-NGECINDDKHCDGTSDC--KD-GSDEFDCD--MVLCKEPHW
ADM34986.1_1_B_tabaci_B           ------IQREWSSISKGSWCTALLVV--IA-VFCTFVQSSSSYECVGPSH
CAJ19121_Blattella_germanica      -QGTFECH-NGACISETKHCDGHVDC--TD-GSDEVDCN--QVFCKEPDW
H2CLV8_Antheraea_pernyi           ------------KVVLLAIVLCTTSC--AGQFVDEMQVY--EKECLGEDV
XP_008180459_Acyrthosiphon_pisum  TKYEFNCG-NGTCINKTRLCDGIVDC--MDNGADEDDCDISKKPCLAPEY
UPI000252AABD_Apis_florea         -----------YRNLFVFFVLTNLYC--SSIDTTVIRCD-------PPDF
UPI0001758603_Tribolium_castaneum --------------------------------------------------
T1KD20_Tetranychus_urticae        -----------YLLTILLVISTSLSC--LAQTSASLSTK-------GP--
UPI0002063B8E_Apis_mellifera      --------------------------------------------------
E2FLQ4_Bombyx_mori                ------------KVVLLAIVLCTTSC--VGQFVDEMQVY--EKECLGEDV
```

LDLa

```
Bemisia_tabaci_Asia_1            FECTNHR--CISMDLRCDGDDDCNDGSDEHGCNVD--------KSKNETC
T1E9W2_Anopheles_aquasalis       ------------LDLMCDGIDHCPDGSDE---------------------
P98163_Drosophila_melanogaster   FPCAQPHGACLAAELMCNGIDNCPGGEDELNCPVRPGFRFGDTAHRMRSC
B4Q2Q9_Drosophila_yakuba         FPCAQPHGACLAAELMCNGIDNCPGGEDELNCPVRPGFRFGDTAHRMRNC
B4IGA9_Drosophila_sechellia      FPCAQPHGACLAAELMCNGIDNCPGGEDELNCPVRPGFRFGDTAHRMRNC
K4MUW4_Actias_selene             FPCMSGG--CIQQSQYCDGKVDCDDGTDENYCLDH-------KPDAQFCN
Q1T728_Rhyparobia_maderae        FRCRTGR--CISSSLRCDTDDDCGDWSDEEDCFPT--------VSEPARC
O76952_Aedes_aegypti             YRCKHDK-SCISATFLCDKHDDCPLGDDERNCENF------EVPHVPVPC
Q6X0I2_Solenopsis_invicta        FQCNSGE--CIPVDKKCDYIDHCIDGSDEDFECDH------LDEKSFITC
ADE34166.1_Nilaparvata_lugens    FECGNGN--CIDGDMVCNDVRDCLDGSDEDEQLCH-TQHQEHHINYPECD
D5KXW4_Nilaparvata_lugens        FECGNGN--CIDGDMVCNDVRDCLDGSDEDEQLCH-TQHQEHHINYPECD
BAC02725_Periplaneta_americana   FRCHNGR--CTSKSFHCDGVDDCGGWSDEEDCYEM--------ESKPANC
ADM34986.1_1_B_tabaci_B          FECTNHR--CISMDLRCDGDDDCNDGSDEHGCNVD--------KSKNETC
CAJ19121_Blattella_germanica     FRCRNGR--CISSGMRCDDDDDCGDWSDEDDCHIE---------HVPKNC
H2CLV8_Antheraea_pernyi          FPCMSGG--CIQQSQYCDGKVDCDDGTDENYCLDH-------KPDAQFCN
XP_008180459_Acyrthosiphon_pisum FECDNKR-KCIPKEFVCDKSNECTDKSDESHCKET-------SSSLTQSC
UPI000252AABD_Apis_florea        FHCKNGK--CISSLFRCDGENECGDNSDEMDCNGE-------TNLHMIRC
UPI0001758603_Tribolium_castaneum --------------------------------------------------
T1KD20_Tetranychus_urticae       --------------------------------------------------
UPI0002063B8E_Apis_mellifera     --------------------------------------------------
E2FLQ4_Bombyx_mori               FPCMSGG--CIQQSQYCDGKVDCDDGTDENYCLDH-------KPDAQFCN
```

LDLa

```
Bemisia_tabaci_Asia_1            ASTQFDCG-QGQCIPRSWVCDGNADCEDGKDEGAAGCAESHCAA--SEWE
T1E9W2_Anopheles_aquasalis       ---------------------------------TIGCIDIDSKC--SGFL
P98163_Drosophila_melanogaster   SKYEFMCQQDRTCIPIDFMCDGRPDCTDKSDE-VAGCKQAEITCPGEGHL
B4Q2Q9_Drosophila_yakuba         SKYEFTCQQDRTCIPIDFMCDGRVDCTDKSDE-VAGCKQAEATCSVEGHL
B4IGA9_Drosophila_sechellia      SKYEFTCQQDRTCIPIDWMCDGRPDCTDKSDE-VAGCKQAEVTCPGEGHL
K4MUW4_Actias_selene             ETHQFMCRDSKKCIPNHWICNNDIDCDDGSDE-LNCTLVPVATGKCKGFL
Q1T728_Rhyparobia_maderae        KKDEWHCINDNNCIPTDWVCDGKQDCMDGTDE-LQGCSSVDCS---DGFV
O76952_Aedes_aegypti             SKFEFTCT-DKMCIPLDLVCDGVSHCLDGSDE-TIGCKDIENKC--KGFV
Q6X0I2_Solenopsis_invicta        AKDQFKCK-NQECIPAAKYCDMVNDCLDESDE-HDGCVKHLNCT--NKFL
ADE34166.1_Nilaparvata_lugens    NATQFTCD-DKMCIPWAWVCDGEPNCLDDSDEKTSLCQKHHCSR--EEFQ
D5KXW4_Nilaparvata_lugens        NATQFTCD-DKMCIPWAWVCDGEPNCLDDSDEKTSLCQKHHCSR--EEFQ
BAC02725_Periplaneta_americana   TADEWRCV-DNNCIFMDWVCDGKQDCMDGSDE-LQGCSHKMSCE--DGFV
ADM34986.1_1_B_tabaci_B          ASTQFDCG-QGQCIPRSWVCDGNADCEDGKDEGAVGCAESHCAA--SEWE
CAJ19121_Blattella_germanica     TDSEWRCM-DNNCIIIDWVCDGRQDCMDGSDE-LQGCSTVLSCH--DGFM
H2CLV8_Antheraea_pernyi          ETHQFMCRDSKKCIPNHWICNNDIDCDDGSDE-LNCTLVPVATGKCKGFL
XP_008180459_Acyrthosiphon_pisum REGKFKCS-DGTCIPMAWHCDAEIDCHDGSDEDGEKCRYTTLCT--SGYM
UPI000252AABD_Apis_florea        TTDEFQCA-DHSCIPEEKFCDAKSDCYDGSDE-YIGCVKELKCN--NSFR
UPI0001758603_Tribolium_castaneum --------------------------------------------------
T1KD20_Tetranychus_urticae       --------VESVQPSIQVNSSEVTSTNFSDYNETISTTPLSST---TFI
UPI0002063B8E_Apis_mellifera     -----EKT-NAVTIPTKWIAMA------SNDE-YVGCVKELKCN--NNFR
E2FLQ4_Bombyx_mori               ETHQFMCRDSKKCIPNHWICNNDIDCDDGSDE-LNCTLVPVATGKCKGFL
```

LDLa

```
Bemisia_tabaci_Asia_1            CPHNHRCIP-NDYICDGDDDCGDNSDENDCTGKNNF--TECTSA------
T1E9W2_Anopheles_aquasalis       C-RNKHCLRTSSWVCDGMRDCSDGSDEEHC-------VHECTLE------
P98163_Drosophila_melanogaster   C-ANGRCLRRKQWVCDGVDDCGDGSDERGC-------LNLCEPQ------
B4Q2Q9_Drosophila_yakuba         C-ANGRCLRRKQWVCDGVDDCGDGSDEKGC-------LNLCEPQ------
B4IGA9_Drosophila_sechellia      C-ANGRCLRRKQWVCDGVDDCGDGSDERGCSNL-------CEPQ------
K4MUW4_Actias_selene             C-GDGKCIS-SLWLCDGSYDCKDKSDENSPENCRHSLLSHSMLSGSDCQD
Q1T728_Rhyparobia_maderae        C-NNHHCIP-VTFHCDGSDDCGDGSDERNCSSILHILPEDCTHE------
O76952_Aedes_aegypti             C-KNKRCINSHDWVCDGIDDCGDGSDEENC-------FIGCDLE------
Q6X0I2_Solenopsis_invicta        C-TDGHCIN-KEWVCDGRNDCPDGNDEWNCKANKTSSASSCKTE------
ADE34166.1_Nilaparvata_lugens    C-RDGHCIE-EDFKCDGSPDCKDGSDEENCPVGVYSMDDECTLD------
D5KXW4_Nilaparvata_lugens        C-RDGHCIE-EDFKCDGSPDCKDGSDEENCPVGVYSMDDECTLD------
BAC02725_Periplaneta_americana   C-GNYHCIP-NSFLCDGFDDCGDNSDEKLCPSVRNVPPEDCKLE------
ADM34986.1_1_B_tabaci_B          CPHNHRCIP-NDYICDGDDDCGDNSDEDDCTGKNNF--TECTSA------
CAJ19121_Blattella_germanica     C-KNGHCLP-ITFHCDGSDDCGDNSDEDYCPSVHYIPPENCTTD------
H2CLV8_Antheraea_pernyi          C-GDGKCIS-SLWLCDGSYDCKDKSDENSPENCRHSLLSHSMLSGSDCQD
XP_008180459_Acyrthosiphon_pisum C-KNFNCVS-KTWRCDGKDDCGDGSDETHC-DIQLVEPEKCLTE------
UPI000252AABD_Apis_florea        C-KDSHCIR-KEWVCDGVPDCPDKSDEEKCES-NITSIEECNNE------
UPI0001758603_Tribolium_castaneum --------------------------------------------------
T1KD20_Tetranychus_urticae       N----------------QANTEDDGDLKTSTN------------------
UPI0002063B8E_Apis_mellifera     C-KDSHCIR-NEWVCDGVPDCPDKSDEEKCENN-IVSIEKCNNE------
E2FLQ4_Bombyx_mori               C-GDGKCIS-SLWLCDGSYDCKDKSDENSPENCRHSLLSHSMLSGSDCQE
```

LDLa

```
Bemisia_tabaci_Asia_1              ----FGKFLCKNRNQCIDDTLLCNGHPDCKDGSDEG-GHCASKAQVAADC
T1E9W2_Anopheles_aquasalis         ----HGKFECANNDTCIAIQEVCNGKADCDDGSDEG-AGCK-----NDSC
P98163_Drosophila_melanogaster     ----KGKFLCRNRETCLTLSEVCDGHSDCSDGSDET-DLCHS----KPDC
B4Q2Q9_Drosophila_yakuba           ----KGKFLCRNRETCLVLSEVCDGHSDCSDGSDET-EFCHS----KPDC
B4IGA9_Drosophila_sechellia        ----KGKFLCRNRETCLTLSEVCDGHSDCSDGSDET-DLCHS----KPDC
K4MUW4_Actias_selene               WLGGRRQYKCTDSSFCLPSEMMCDGMQDCKDGSDER-SFCANW---HTMC
Q1T728_Rhyparobia_maderae          ----RNFHLCRDNRTCISLDELCDGVRNCPDYSDEG-IKCNES---KAAC
O76952_Aedes_aegypti               ----HGKFECADNSTCVDLKLVCDGKDDCGDHSDEG-GSCNSK-----EC
Q6X0I2_Solenopsis_invicta          ----NYQYMCANHR-CISLKVVCDKKDDCGDGSDEG-PGCT-----QFNC
ADE34166.1_Nilaparvata_lugens      ----NERFPCHDRTLCIDISALCNNKRDCFDGSDEG-GLCHE----KIDC
D5KXW4_Nilaparvata_lugens          ----NERFPCHDRTLCIDISALCNNKRDCFDGSDEG-GLCHE----KIDC
BAC02725_Periplaneta_americana     ----KNLFLCADRQECVEVRELCDGTPHCYDGSDEG-PACNAS---RAAC
ADM34986.1_1_B_tabaci_B            ----FGKFLCKNRNQCIDDSLLCNGHPDCKDGSDEG-GHCASKAQVATDC
CAJ19121_Blattella_germanica       ----KNLHLCHDGRTCISLNELCDEVQHCPDHSDEG-PGCNNS---KITC
H2CLV8_Antheraea_pernyi            WLGGRRQYKCTDSSFCLPSEMMCDGMQDCKDGSDER-SFCANW---HTMC
XP_008180459_Acyrthosiphon_pisum   ----NRKFLCHDHKKCIDVRNVCDYNNDCLDASDEG-GLCNNKTDFKSEC
UPI000252AABD_Apis_florea          ----YDRYLCKNQR-CIFLNATCDEKNDCGDNSDEDLDACKKA---DASC
UPI0001758603_Tribolium_castaneum  --------------------------------------------------
T1KD20_Tetranychus_urticae         -----------------------------YSGYDNG--------------
UPI0002063B8E_Apis_mellifera       ----HDRYLCKNQR-CIFLNATCNEKNDCGDNSDEDLDACKKA---DASC
E2FLQ4_Bombyx_mori                 WLGGRRQYKCTDSSFCLPSEMMCDGMQDCKDGSDER-PFCANW---HTMC
```

EGF

```
Bemisia_tabaci_Asia_1              A-KLNC-THS--CVESPDG-PVCVCGSGYHL--EG---NVCEDINEC-LE
T1E9W2_Anopheles_aquasalis         A-ELKCGDRP--CKRMPNGRSVCLCETGFRFNNVT---HQCEDINEC-DR
P98163_Drosophila_melanogaster     D-AKKC-ALGAKCHMMPASGAECFCPKGFRLAKFE---DKCEDVDECKEQ
B4Q2Q9_Drosophila_yakuba           E-TKKC-PLGAKCHMMSTSGAECICPKGFRQAKFE---DKCEDIDECKER
B4IGA9_Drosophila_sechellia        D-AKKC-ALGAKCHMMPAGGAECFCPKGFRLAKFE---DKCEDIDECKEQ
K4MUW4_Actias_selene               A-NHTCLGDKASCVPDRAG-PTCECLNHLNLRRYNTSTGACDDIDECALA
Q1T728_Rhyparobia_maderae          K-AIKC-DHL--CVPTPRG-AICVCKVGYTMA-KN---KTCIDIDEC-LK
O76952_Aedes_aegypti               D-SMRC-PEG--CKATPHG-AVCLCKPGFRFNKKS---KVCEDINEC-ER
Q6X0I2_Solenopsis_invicta          S-SAGCQSN---CHQTPKG-SVCTCKPGYKLQKDN---RTCNDIDEC-QA
ADE34166.1_Nilaparvata_lugens      NGKANCSIEQ--CFQSPSG-QICLCSKGYKH--DN---GVCVDIDEC-KE
D5KXW4_Nilaparvata_lugens          NGKANCSIEQ--CFQSPSG-QICLCSKGYKH--DN---GVCVDIDEC-KE
BAC02725_Periplaneta_americana     P-TVGC-SHQ--CIPSPQG-PLCVCQVGYKTV-DN---KTCVDVDEC-ME
ADM34986.1_1_B_tabaci_B            A-KLNC-THS--CVESPDG-PVCVCGSGYHL--EG---NVCEDINEC-LE
CAJ19121_Blattella_germanica       R-TAWC-NHR--CIPTPQG-PQCVCQTGYTMAVNN----TCVDIDEC-LE
H2CLV8_Antheraea_pernyi            A-NHTCLGDKASCVPDRAG-PTCECLNHLNLRRYNTSTGACDDIDECALA
XP_008180459_Acyrthosiphon_pisum   N-SMNC-SAAFECLRKPHG-IMCVCPKGMHY--LN---NKCSDINEC-EQ
UPI000252AABD_Apis_florea          KLTAKC-QHN--CRKTPKG-GQCSCRSGYKLI-NN---QTCTDINEC-DN
UPI0001758603_Tribolium_castaneum  --------------------------------------------------
T1KD20_Tetranychus_urticae         --IVIASPKPATVLPNPCG-----------------------------NH
UPI0002063B8E_Apis_mellifera       KLTAKC-EHN--CRKTPKG-AQCSCRSGYKLT-NN---QTCTDINEC-DN
E2FLQ4_Bombyx_mori                 A-NHTCLGDKASCVPDRAG-PTCECLNHLNLRRYNTSTGACDDIDECALA
```

Calcium binding EGF

```
Bemisia_tabaci_Asia_1              WGTCDQMCENT-VGGYICECEPGYKLESNGRTCKA-----EEGEGLLIYS
T1E9W2_Anopheles_aquasalis         YGLCSQGCINT-PGSFRCTCVDQFELKRDQRTCEL----TTGTEALMLYT
P98163_Drosophila_melanogaster     DDLCSQGCENT-SGGYRCVCDAGYLLDKDNRTCRAVVYGSKEQQPLLLYT
B4Q2Q9_Drosophila_yakuba           DDLCSQGCENT-SGGYQCVCDAGYLLDKDNRTCRAIVHGSIEQQPLLLYT
B4IGA9_Drosophila_sechellia        DDLCSQGCENT-SGGYRCVCDAGYLLDKDNRTCRAVVHGSKEQQPLLLYT
K4MUW4_Actias_selene               RPQCSHYCVNA-DGHFTCECADGYFKDELKYLCYA-----TGPEPLLFYS
Q1T728_Rhyparobia_maderae          YGICDQKCKNL-PGSYSCYCDEGYFLADDNHSCKA-----TGADPLLLFA
O76952_Aedes_aegypti               YGLCSQGCENT-PGSFKCTCVDKFKLKDDSRTCEL----DDSTEPLLLYT
Q6X0I2_Solenopsis_invicta          YGICDQDCMNV-PGSYACTCQREYYLENDKRTCKA-----RAGEATLVFS
ADE34166.1_Nilaparvata_lugens      FGICDQKCSNL-VGGFRCSCDPGYALQKDGHTCRA----EGGKEPLIYFS
D5KXW4_Nilaparvata_lugens          FGICDQKCSNL-VGGFRCSCDPGYALQKDGHTCRA----EGGKEPLIYFS
BAC02725_Periplaneta_americana     YGICDQRCRNL-QGSYSCYCDEGYEVGSDKRSCKA-----TGPDALMLFS
ADM34986.1_1_B_tabaci_B            WGTCDQMCENT-VGGYICECEPGYKLESNGRTCKA-----EEGEGLLIYS
CAJ19121_Blattella_germanica       YGICDQKCLNM-PGKYDCYCDEGYELAEDKRTCRA-----TGADPLLVFA
H2CLV8_Antheraea_pernyi            RPQCSHYCVNA-DGHFTCECADGYFKDELKYLCYA-----TGPEPLLFYS
XP_008180459_Acyrthosiphon_pisum   YGICDQVCLNL-EGSYTCSCDPHYEL-VDNHKCKI-----KGLNPELLYS
UPI000252AABD_Apis_florea          YGICDQQCINS-AGSYTCFCQPGYVMQDDKKTCKA-----EGGEATMVFS
UPI0001758603_Tribolium_castaneum  --------------------------------------------------
T1KD20_Tetranychus_urticae         NGNCSDICLDLGNGTRRCGCNYG-VLSSDDLSCEA-------PDSFVVFA
UPI0002063B8E_Apis_mellifera       YGICDQQCINN-AGSYSCLCQPGYIMQDDKKTCKA-----DGGEATMVFS
E2FLQ4_Bombyx_mori                 RPQCSHYCVNA-DGHFSCECADGYFKDELKYLCYA-----TGPEPLLFYS
```

```
Bemisia_tabaci_Asia_1              SLKKIKSLYLTS----RISMTVASEVPY-----ATGVSFDGQHVYWTTVL
T1E9W2_Anopheles_aquasalis         TQRSIGALYLSS----LHQYYVAKELSQ-----VIGVSYDGQHVYWTDIA
```

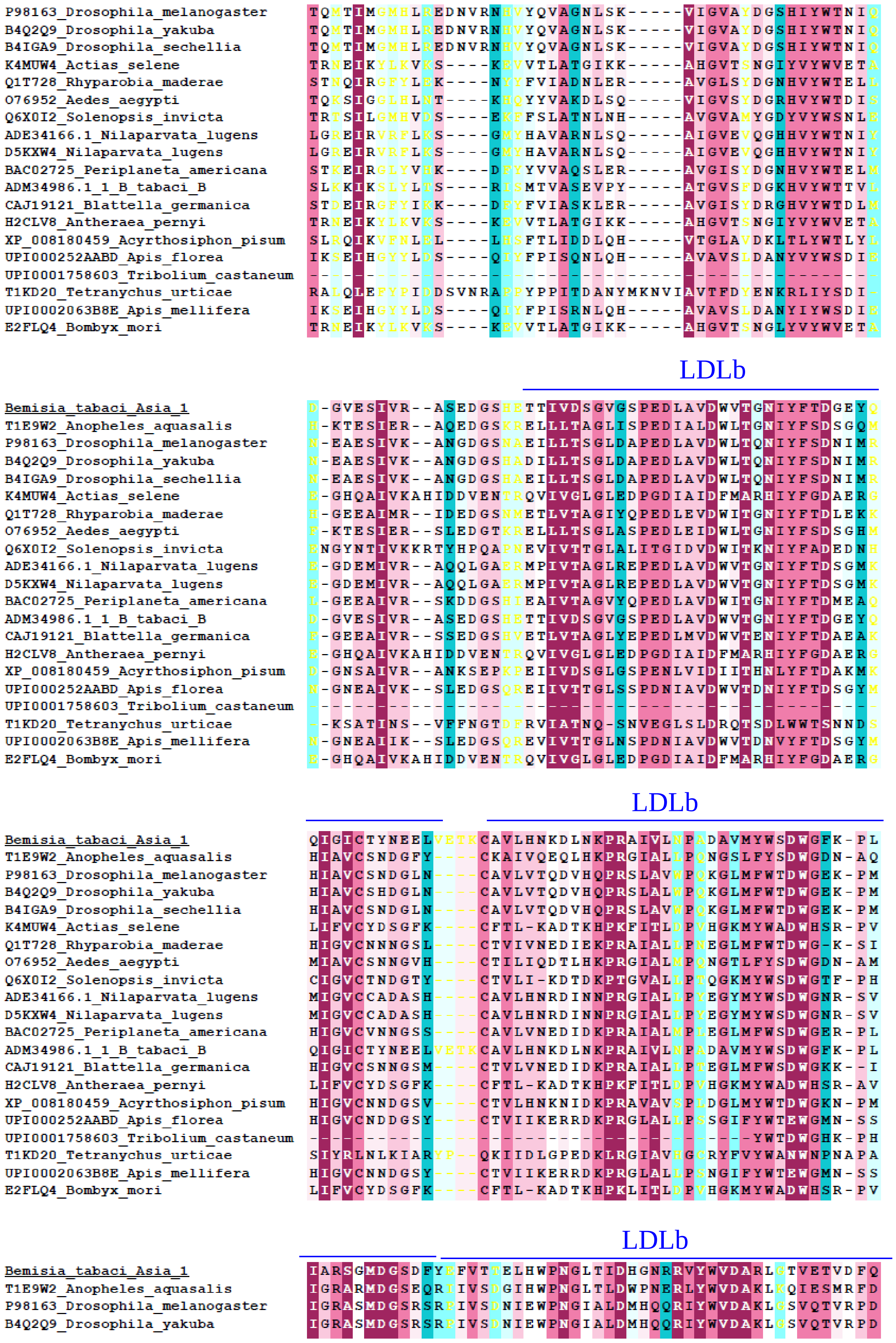
LDLb
LDLb
LDLb
Bemisia_tabaci_Asia_1
T1E9W2_Anopheles_aquasalis
P98163_Drosophila_melanogaster
B4Q2Q9_Drosophila_yakuba
B4IGA9_Drosophila_sechellia
K4MUW4_Actias_selene
Q1T728_Rhyparobia_maderae
O76952_Aedes_aegypti
Q6X0I2_Solenopsis_invicta
ADE34166.1_Nilaparvata_lugens
D5KXW4_Nilaparvata_lugens
BAC02725_Periplaneta_americana
ADM34986.1_1_B_tabaci_B
CAJ19121_Blattella_germanica
H2CLV8_Antheraea_pernyi
XP_008180459_Acyrthosiphon_pisum
UPI000252AABD_Apis_florea
UPI0001758603_Tribolium_castaneum
T1KD20_Tetranychus_urticae
UPI0002063B8E_Apis_mellifera
E2FLQ4_Bombyx_mori

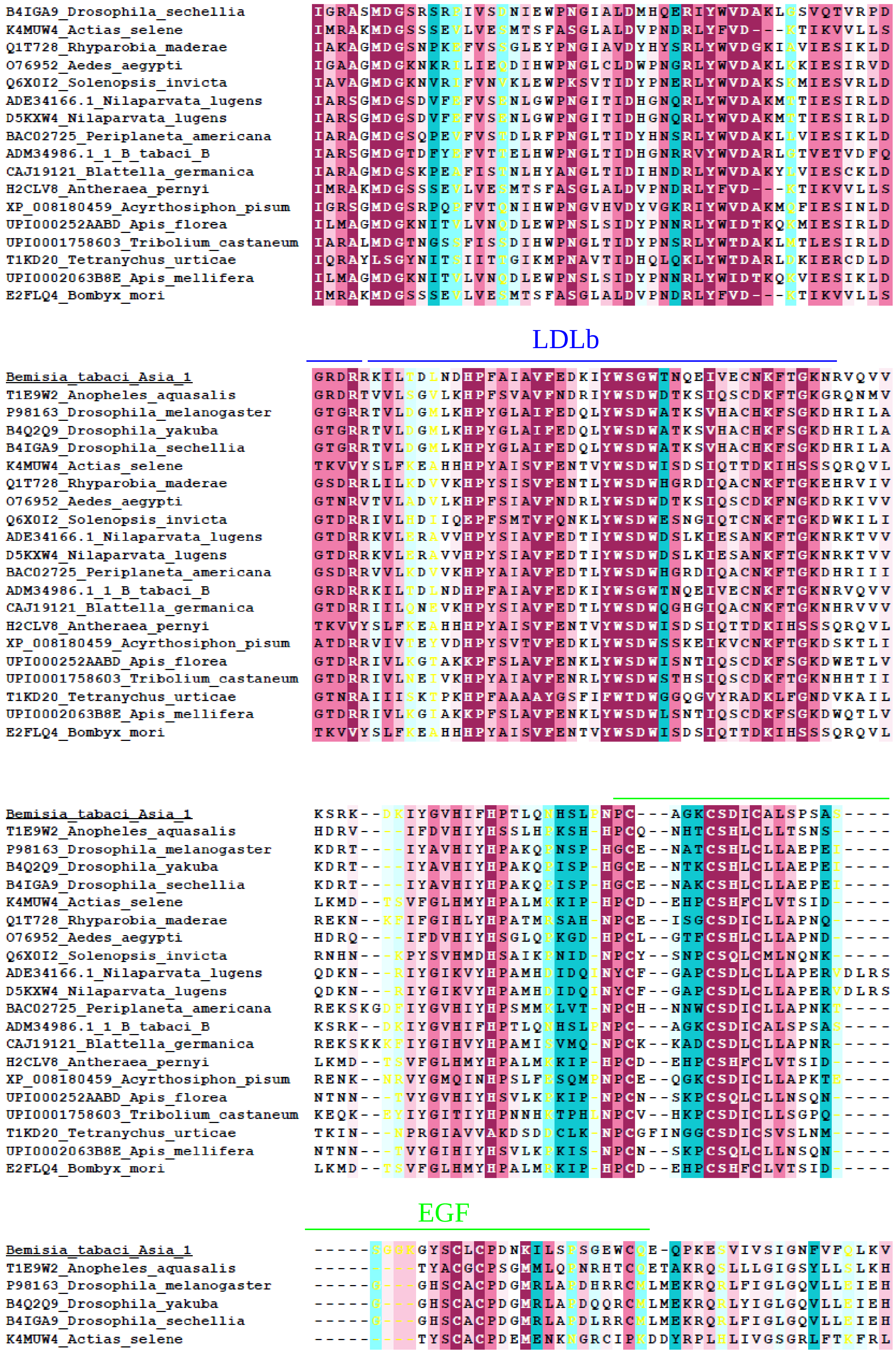
LDLb
EGF

```
Q1T728_Rhyparobia_maderae            ---------TYTCGCPEHKVRGLDKHSCLDSAKQEVMVVSSDHKIYVVGH
O76952_Aedes_aegypti                 ---------SYSCACPYGMSLKADKHSCRETVKRQYLLVGIANYLVTLET
Q6X0I2_Solenopsis_invicta            ---------GYTCGCTLDKKLNADKHTCQDVKKNQHLLIIQGRKFINYYH
ADE34166.1_Nilaparvata_lugens        SPSPAGNSRRYSCACPDGKQLAANNHSCIETEKKQELVIGNRDRLLSYQH
D5KXW4_Nilaparvata_lugens            SPSPAGNSRRYSCACPDGKQLAANNHSCIETEKKQELVIGNRDRLLSYQH
BAC02725_Periplaneta_americana       -----Y---TYTCACPENKQLGADKHTCHEIRKQELVVVAAGHKLTAVGH
ADM34986.1_1_B_tabaci_B              -----SGGKGYSCLCPDNKILSPSGEWCQE-QPKESVIVSIGNFVFQLKV
CAJ19121_Blattella_germanica         ---------TYTCACPEHKVRGADMHSCHESLKQEVMIGAANNKLYAVGH
H2CLV8_Antheraea_pernyi              ---------TYSCACPDEMENKNGRCIPKDDYRPLHLIVGSGRLFTKFRL
XP_008180459_Acyrthosiphon_pisum     -----INSKGYSCACPDDKKLSEDGIFCITIAIPPTLIVGTPTSIIEIEQ
UPI000252AABD_Apis_florea            ---------GYTCACSLDKELNYDNHTCRAIKKKMHLIIAAGNTFIDYYH
UPI0001758603_Tribolium_castaneum    ---------GFTCACPQNKELSSNGFTCRDLEKKQKLVLGAGNLLIEVEH
T1KD20_Tetranychus_urticae           ---------TVQCSCFEGRTL-LDGHRCMN--------------------
UPI0002063B8E_Apis_mellifera         ---------GYTCACSLDKELNYDNHTCRAIKKKMHLIIAAGNTFIDYYH
E2FLQ4_Bombyx_mori                   ---------TYSCACPDEMGNKNGRCIPKDDYRPLHLIVGSGRLFTKFRL
```

LDLb

```
Bemisia_tabaci_Asia_1                T-LGKQYI---HPLPVNN-----LQSVSAIVYNSFDGSLLIADPD-----
T1E9W2_Anopheles_aquasalis           HPFGRHEEGKGEPLPIN---------ISRLAFNSLTGEVLVADNV-----
P98163_Drosophila_melanogaster       TAFGRHQVSKSYTLPCL---------INEMVYNRINGSLIIADND-----
B4Q2Q9_Drosophila_yakuba             TAFGRHQVSKSYTLPCL---------INEMVYNRINGTLLIADND-----
B4IGA9_Drosophila_sechellia          TAFGRHQIRKSYTLPCL---------INEMVYNRINGSLIIADND-----
K4MUW4_Actias_selene                 DAMGNPHS---HVTNFS------LGRVQAMTYDSVRDRLYVYDGR-----
Q1T728_Rhyparobia_maderae            QFLGRQTF---TQMTMDS-----VHHIGAITYNSLTGNVIIFDDA-----
O76952_Aedes_aegypti                 QTFGRHES---SQADAYQ-----IF-FHRMAFNSITGEIFVADNR-----
Q6X0I2_Solenopsis_invicta            EFLGKPKV---MTLSLQHMSQQSYNNLVNIISDPLSGQIIICHLQLSTPF
ADE34166.1_Nilaparvata_lugens        KQLGRVHI---EELPLDQGNAM-IGDISALQYNSQDGSLIVGDSY-----
D5KXW4_Nilaparvata_lugens            KQLGRVHI---EELPLDQGNAM-IGDISALQYNSQDGSLIVGDSY-----
BAC02725_Periplaneta_americana       QFLGRQTL---YDMTLKN-----VHTIGAVTYNSLTDHIIIFDSE-----
ADM34986.1_1_B_tabaci_B              T-LGKQYI---HPLPVNN-----LQSVSAIVYNSFDGSLLIADPD-----
CAJ19121_Blattella_germanica         QFLGKQTF---YEMSLQA-----VNHIGAITYNSLTGHLVIFDAV-----
H2CLV8_Antheraea_pernyi              DAMGNPHS---HVTNFS------LGRVQAMTYDSVRDRLYVYDGR-----
XP_008180459_Acyrthosiphon_pisum     EHLGRQKA---KKISLKND----ISSISALTYNSLSGDIIIYDSR-----
UPI000252AABD_Apis_florea            ELLGKPKM---ATSVM-------LKRVTAIAYNPLTDGLLASDQL-----
UPI0001758603_Tribolium_castaneum    QVLGRHEV---NAMPIT------VKKMGALTYSSVENVIYVSDLE-----
T1KD20_Tetranychus_urticae           --------------------------------------------------
UPI0002063B8E_Apis_mellifera         ELLGKPKM---ATSIM-------LKRVTAIAYNPLTGGLLASDQL-----
E2FLQ4_Bombyx_mori                   DAMGNPHS---HVTNFS------LGRVQAMTYDSVRDRLYVYDGR-----
```

LDLb

```
Bemisia_tabaci_Asia_1                -AKM--IYSY-QLNTDTMETLIDLKVGYVSALAYDPIGRNLYWCDKEAGT
T1E9W2_Anopheles_aquasalis           -QKA--IFAV-NLETKQTRALVTTGIGSVASLALDYLSNTVFWCDTDRST
P98163_Drosophila_melanogaster       -QRL--ILEF-QPESHESNVLVRSNLGNVSALAFDHLSRNLYWADTERAV
B4Q2Q9_Drosophila_yakuba             -QRL--ILEF-QPENRETNVLVRANLGNVSALAFDHLSRNLYWADTERGV
B4IGA9_Drosophila_sechellia          -QRL--ILEF-QPETHETNVLVRANLGNVSALSFDYLSRNLYWADTERAV
K4MUW4_Actias_selene                 -EHSISYTNMSDFTHGKVFALIKFGPENVVDMDYDYVSDSLYMLDSGSGY
Q1T728_Rhyparobia_maderae            -QHK--LYNL-GVKTGKLSQLAS-DIGSIEGMDFDYLGNNLYWCDAQKAP
O76952_Aedes_aegypti                 -QKA--IFTV-DPKTKSSQKLITTGIGNISALAFDFLGNNLYWTDSERST
Q6X0I2_Solenopsis_invicta            LTSTTDILRY-DPVHHSSEKIVTIN-KIFFELAFDYIGNNLYTTNTVNQS
ADE34166.1_Nilaparvata_lugens        -NRK--MSSV-DLKTLQARDIVTTGVGRIEGIAFDNLGSNIYWTDSELGK
D5KXW4_Nilaparvata_lugens            -NRK--MSSV-DLKTLQARDIVTTGVGRIEGIAFDNLGSNIYWTDSELGK
BAC02725_Periplaneta_americana       -QQK--LFTL-GLKTMKLSLLLS-HVGKIDAMDFDYMGNNLYWCDGERAT
ADM34986.1_1_B_tabaci_B              -AKM--IYSY-QLNTDTMETLIDLKVGYVSALAYDPIGRNLYWCDKEAGT
CAJ19121_Blattella_germanica         -QHK--LFNL-GLKTMKLSQLAS-DVGYIDGMDFDYLGNNIYLCDGQKAT
H2CLV8_Antheraea_pernyi              -EHSISYTNMSDFTHGKVFALIKFGPENVVDMDYDYVSDSLYMLDSGSGY
XP_008180459_Acyrthosiphon_pisum     -SKK--LFTF-DFVKMKLSDLDVPEISSIYSLEFDNHGNNLYWCDRTRKT
UPI000252AABD_Apis_florea            -TDT--IFHL-NTHTGEVKSVITIENEILGGMDFDYIGNNVYLSDVKHKT
UPI0001758603_Tribolium_castaneum    -LRK--IISV-NLHTEMAKPIDVGSLGHVTAMDYDYLGNNLYWCDSLRDT
T1KD20_Tetranychus_urticae           -NRT--------------------AEAKCNSTQFE--------CD-----
UPI0002063B8E_Apis_mellifera         -TDT--IFHL-NTHIGEVKSIITIENEILGGMDFDYIGNNVYLSDVKHKT
E2FLQ4_Bombyx_mori                   -EHSISYTNMSDFTHGKVFALIKFGPENVVDMDYDYVSDSLYMLDSGSGY
```

```
Bemisia_tabaci_Asia_1                VEVFSFFSHRRKLLLREFDDEKPFAMTLIPEEGLMFVIAKAHD-----HL
T1E9W2_Anopheles_aquasalis           IEIYSLDTHHRSIVQHFLGGDSPVALALIPEIGKMFIALRSRAAPW--HT
P98163_Drosophila_melanogaster       IEVLSLQTRHRALIRFFPGQEVPIGLTVMPAEGYLYVVLKAKR-----HS
B4Q2Q9_Drosophila_yakuba             IEVLSLQTRHRALIRFFPGQEVPIGLTVMPAEGYLYVVLKAKR-----HS
B4IGA9_Drosophila_sechellia          IEVLSLQTRHRALIRFFPGQEVPIGLTVMPAEGYLYVVLKAKR-----HS
K4MUW4_Actias_selene                 IEVLSLRTLHRAVVYRFTDRETPVSFCVLPHYGKMLVAVMQTDNDN--RI
Q1T728_Rhyparobia_maderae            VEVMSLTTHERTILLHSFEGEVPLDVAVVPEEGVMFLSMSRHLSSSE-GP
O76952_Aedes_aegypti                 VEVFSLQTRHRAILQHYLGQDIPVGLAIVSEMGKMFIALRSPLPVP--HT
```

```
Q6X0I2_Solenopsis_invicta           IEVINLNT--KAMTAFYFKDEVPKYIALAPEESKMFVAFQKSMHSIS-GL
ADE34166.1_Nilaparvata_lugens       VEVINTINHHRKTIMNHLQGDIPRGIAVIPSEGIMYVSLTSPT-----TA
D5KXW4_Nilaparvata_lugens           VEVINTINHHRKTIMNHLQGDIPRGIAVIPSEGIMYVSLTSPT-----TA
BAC02725_Periplaneta_americana      VEILSLNTMERAILIHTLEGEIPLDVAVIPEEGVMFVAFSRHVIGD--GP
ADM34986.1_1_B_tabaci_B             VEVFSFFSHRRKLLLREFDDEKPFAMTLIPEEGLMFVIAKAHD-----HL
CAJ19121_Blattella_germanica        IEILSLTTLERPVLLHSFEGEVPLDIAVVPEEGIMFIAMSRHLTSSENGP
H2CLV8_Antheraea_pernyi             IEVLSLRTLHRAVVYRFTDRETPVSFCVLPHYGKMLVAVMQTDNDN--RI
XP_008180459_Acyrthosiphon_pisum    LDVLSLSTKTHTTIFKEIEGHIPFAVTLVPDKSFMFVAMKEGS-----HI
UPI000252AABD_Apis_florea           IEVHSLNSKEKTIFYF---KDEPYDIALVPEEGIMMVVFRRDE-----LY
UPI0001758603_Tribolium_castaneum   IEVYNFNTNSRKILLHDTNGETPESIALVPEEGIMFVAFRRFGQR---DG
T1KD20_Tetranychus_urticae          --------------------------------------------------
UPI0002063B8E_Apis_mellifera        IEVHSLNSKQKTIFYF---KDEPYDIALVPEEGIMMVVFRSDG-----LY
E2FLQ4_Bombyx_mori                  IEVLSLRTLHRAVVYRFTDRETPVSFCVLPHYGKMLVAVMQTDNDN--RI
```

LDLb

```
Bemisia_tabaci_Asia_1               HIDRINM-DGSLSTLTHMTSLK--LQGP-DVALHYDSDSRRVYWADHSAG
T1E9W2_Anopheles_aquasalis          HIDQLAL-TGR-GPHAHVMEDR--IGSNGSIGFHVDHDLRAVFWSDQSSG
P98163_Drosophila_melanogaster      HIDKIPL-SGK-GEQVHVFEDD--LGDD-DIKLVTDYETQTIFWSDSDLG
B4Q2Q9_Drosophila_yakuba            HIDKIPL-SGK-GEQVHVFEDD--LGDD-DIKLVTDYETQTLFWSDSDLG
B4IGA9_Drosophila_sechellia         HIDKIPL-SGK-GEQVHVFEDD--LGDD-DIKLVTDYETQTLFWSDSDLG
K4MUW4_Actias_selene                YVDSIGL-DGD--GRRHIVTVN--IRGP-RIILRFLHGMDNVYLADEGNG
Q1T728_Rhyparobia_maderae           HIDRISM-DGK-GEHTHIVESY--LEGP-TLNLYYDKDMERIFWADPNSG
O76952_Aedes_aegypti                HIDRLDM-TGR-GPHLHVIEER--LSGNGSFNFVIDRDLRTVYWNDMGSS
Q6X0I2_Solenopsis_invicta           TLYEMQM-NGL--GKRKLIREG--LIGP-QLPMYYDRDSKTLFVSDLLPG
ADE34166.1_Nilaparvata_lugens       HIDKLSMASGDVMSRTHIFEEN--LRGP-FIPLFYDGMLHRMYWADSGTR
D5KXW4_Nilaparvata_lugens           HIDKLSMASGDVMSRTHIFEEN--LRGP-FIPLFYDGMLHRMYWADSGTR
BAC02725_Periplaneta_americana      HIDRMNM-DGR-GAHTHVIETS--LDGP-IISLFYDSDLHRVFWTDPNNE
ADM34986.1_1_B_tabaci_B             HIDRINM-DGSLSTLTHMTSLK--LQGP-DVALHYDSDSRRVYWADHSAG
CAJ19121_Blattella_germanica        HIDRMTM-DGR--EHFHIVESG--LDGP-IISLHYDKELHRVFWTDPNEG
H2CLV8_Antheraea_pernyi             YVDSIGL-DGD--GRRHIVTVN--IRGP-RIILRFLHGMDNVYLADEGNG
XP_008180459_Acyrthosiphon_pisum    HIDRIDM-DGSIQSLVHMVEYG--LTGD-EIALHFDMITRLLYFTDYKNG
UPI000252AABD_Apis_florea           HIDLMNM-NGL-GPKITIVGNKTVLIGP-KISLSYDGDFKQLFWSDQGSG
UPI0001758603_Tribolium_castaneum   HVDRFLM-DGT--SRTHPIENG--LLGP--ISLYYDSDIHRILIADSGTR
T1KD20_Tetranychus_urticae          --------DG-------------------VCIPYELTCDLI---------
UPI0002063B8E_Apis_mellifera        YIDLMNM-NGL-GPKITIVGNKTLLIGP-KISLSYDGDFKQLFWCDQGSG
E2FLQ4_Bombyx_mori                  YVDSIGL-DGD--GRRHIVTVN--IRGP-RIILRFLHGMDNVYLADEGNG
```

```
Bemisia_tabaci_Asia_1               LIESTDTNGNDRQVYR-D-VSSPLALTDVDRDLYWTSDGRPHLYYSEKAN
T1E9W2_Anopheles_aquasalis          RIEMTSYEGDTRHLYR-DYLRHPVSMTVIGDELFWTGYRSQRLYWSDKHN
P98163_Drosophila_melanogaster      RISYSNYRVPHSQIFR-GKLRRPYSLAMVHHDLFWNELGTPRIYWTHKSN
B4Q2Q9_Drosophila_yakuba            RISYSNYRVPHSQIFR-GKLRRPYSLAMVHHDLFWNELGTPRIYWTHKSN
B4IGA9_Drosophila_sechellia         RISYSNYRVPHSQIFR-GKLRRPYSLAMVHHDLFWNELGTPRIYWTHKSN
K4MUW4_Actias_selene                IIDYLHPEGTGRENFR-ELSTSISSMAVTENYIFWTDRRTPKLYWANIHE
Q1T728_Rhyparobia_maderae           DIRSAAKNGMDEKIYK-TGLVQPFDVSSLGHELFWTDWGYPYVYWADKYD
O76952_Aedes_aegypti                KIEFTSYEGDTRHLFR-EFLRLPVSIAIVGDSIFWTCYRSKRLYWSDKHN
Q6X0I2_Solenopsis_invicta           YIYSHSAQ--DTRILR-SGLKSPHSLTVAGDNLFWIE-SQNKLYSTNFRT
ADE34166.1_Nilaparvata_lugens       RIEHTNWNGEERHTYS-ELNSSPISVTSVGTDIFWSTPGFKNVYYANKLE
D5KXW4_Nilaparvata_lugens           RIEHTNWNGEERHTYS-ELNSSPISVTSVGTDIFWSTPGFKNVYYANKLE
BAC02725_Periplaneta_americana      EIGSAAADGMDQHVFRSDVEGSPIDIASVGRDMFWTMWAHPYLYWASKFN
ADM34986.1_1_B_tabaci_B             LIESTDTNGNDRQVYR-D-VSSPLALTDVDRDLYWTSDGRPHLYYSEKSN
CAJ19121_Blattella_germanica        QIGSAAANGMDQHIYK-HSINNPSDVASLGRDLFWTDWGQPYIYWANKFD
H2CLV8_Antheraea_pernyi             IIDYLHPEGTGRENFR-ELSTSISSMAVTENYIFWTDRRTPKLYWANIHE
XP_008180459_Acyrthosiphon_pisum    IIDSVSEDGTDRRIIK-K-AGFVRDIVSIGYDIVWVSEGSKILNWIDNVD
UPI000252AABD_Apis_florea           RIGSTTIPGFETYILR-TGLSEPVSLALLGDYLFWTQYKSNQLYWTSKTI
UPI0001758603_Tribolium_castaneum   NIESTSVEGDDRHGFR-SLQTNPIGITSLKSEVFWVNRNSKELYWADKYN
T1KD20_Tetranychus_urticae          ---SHCVNGSD---------------------------------------
UPI0002063B8E_Apis_mellifera        RIGSTTIPGFETYILR-TGLSEPVSLAILGNYLFWTQYKSNQLYWTSKTI
E2FLQ4_Bombyx_mori                  IIDYLHPEGTGRENFR-ELSTSISSMAATENYIFWTDRRTPKLYWANIHE
```

```
Bemisia_tabaci_Asia_1               A--SMPVRKINMERFLR-----------SPKDH---YRMFVTAI----IP
T1E9W2_Anopheles_aquasalis          M---GVTKRMTIP---------------MPPGVAIPERIPVAAT----QP
P98163_Drosophila_melanogaster      M---GPRKVIDIMEKDDPAAIMPYVPVATP------NGIPLAAS----SP
B4Q2Q9_Drosophila_yakuba            M---GPRKVIDIMEKGDPAAILPYVPVAMP------NGIPLAAS----SP
B4IGA9_Drosophila_sechellia         M---GPRKVIDIMEKGDPAAIMPYVPVATP------NGIPLAAS----SP
K4MUW4_Actias_selene                T--SHKIRRIELR---------------AFSNS---SQLLLQTTYPPPSP
Q1T728_Rhyparobia_maderae           S--ESLMKRISVD-----------------VHS---QKLRLVAV----RG
O76952_Aedes_aegypti                L---GVTKKITID---------------KPPYGAFPDEIVLLGS----QP
Q6X0I2_Solenopsis_invicta           A--SVKQKTVEFDLSKLNDNMTSLPGHLTPYSR---DAQYVVTL-----R
ADE34166.1_Nilaparvata_lugens       F---KGSKVLDLSN--------------LVYSN---SKIFITAV----TG
```

```
D5KXW4_Nilaparvata_lugens              F---KGSKVLDLSN--------------LVYSN---SKIFITAV----TG
BAC02725_Periplaneta_americana         S--QSRMKRLLLD----------------VEDS---DKLPLVAV----RG
ADM34986.1_1_B_tabaci_B                A--SMPVRKINMERFLR-----------SPKDH---YRMFVTAI----IP
CAJ19121_Blattella_germanica           G--ESRMKRLMLD---------------VPKS----QKLKLQGV----RG
H2CLV8_Antheraea_pernyi                T--SHKIRRIELR---------------AFSNS---SQLLLQTTYPPPSP
XP_008180459_Acyrthosiphon_pisum       E--DRASRALDID---------------NWKTD---SNLYLTVA----NG
UPI000252AABD_Apis_florea              T--KQYQKRITLQ---------------TPEDL---DKMQLIGM-----H
UPI0001758603_Tribolium_castaneum      SGDDDYNKKITLD---------------LPDDI---DRMYLVSV----TP
T1KD20_Tetranychus_urticae             ------------------------------------ESLKLCAN------
UPI0002063B8E_Apis_mellifera           T--KQYQKRITLQ---------------TPENV---DKMQLIGM-----H
E2FLQ4_Bombyx_mori                     T--SHKIRRIELR---------------AFSNS---SQLLLQTTYPPPSP
```

EGF

```
Bemisia_tabaci_Asia_1                  DKTTRDHPCQTNNGKCSHFCL--LTSRNP-KHVCSCPDGMKLADN-GQDC
T1E9W2_Anopheles_aquasalis             IR-RQEHPCRHRNAGCSHICV----AAGLYSAACVCPTGMVFNTSLSKVC
P98163_Drosophila_melanogaster         VG-QESHPCQQQNGGCSHICV----GEGPYHSICLCPAGFVYRDAGNRTC
B4Q2Q9_Drosophila_yakuba               IG-LESHPCQLQNGGCSHICV----GEGPYHSICLCPAGFVYRDAGNRTC
B4IGA9_Drosophila_sechellia            VG-QESHPCQQQNGGCSHICV----GEGPYHSICLCPAGFVYRDAGNRTC
K4MUW4_Actias_selene                   HDPLTQHPCHRDN-PCSQVCVPTYSPTNPYSYKCLCSPGLVFSNG---RC
Q1T728_Rhyparobia_maderae              IRTKPRHPCQIDNGHCSHICA-----LSLKRTVCLCPIGMELKHD-GHTC
O76952_Aedes_aegypti                   LQ-RYDHPCMKQNGGCSHICV----PAGMYSSACICPTGMIFSSPKNTTC
Q6X0I2_Solenopsis_invicta              KDDIPKHDCQKNNGNCSHVCL----PSLITSFICACPPGMELSND-NRTC
ADE34166.1_Nilaparvata_lugens          LDSQRNHPCAVDNGNCSHICL--VSPKKDIMRECACPDGMYLKEG-GREC
D5KXW4_Nilaparvata_lugens              LDSQRNHPCAVDNGNCSHICL--VSPKKDIMRECACPDGMYLKEG-GREC
BAC02725_Periplaneta_americana         VRAQPDHPCHKNNGGCSHICA-----LALKHTVCLCPVGMVLNRD-NKTC
ADM34986.1_1_B_tabaci_B                DKTTRDHPCQTNNGKCSHFCL--LTSRNP-KHVCSCPDGMKLADN-GQDC
CAJ19121_Blattella_germanica           IRAKPNHPCQKENGGCSHICA-----LSQKRMVCLCPMGMQLKKN-EKTC
H2CLV8_Antheraea_pernyi                HDPLTQHPCHRDN-PCSQVCVPTHSPTNPYSYKCLCSPGLVFSNG---RC
XP_008180459_Acyrthosiphon_pisum       IHKNIQHPCQQNNGGCSHICL-----LSEKGKVCGCPPGLVLSNN-SLNC
UPI000252AABD_Apis_florea              KTYVKEHLCRKNNGNCSHVCL---LSNNPSSYICACPPDMMLNID-NRKC
UPI0001758603_Tribolium_castaneum      RKIGLS-PCQRSNGGCSHLCL-----QSHKTIVCACPVDMSLDST-NKTC
T1KD20_Tetranychus_urticae             -RDCPFDFYRCINGKCIRDAM-------LCDGKIDCPDG-----GDEANC
UPI0002063B8E_Apis_mellifera           KTYVKEHPCRKNNGNCSHVCL---LSNNPSSYICACPPDMMLNID-NRKC
E2FLQ4_Bombyx_mori                     HDPLTQHPCHRDN-PCSQVCVPTHSPTNPYSYKCLCSPGLVFSNG---RC
```

LDLa

```
Bemisia_tabaci_Asia_1                  EEIAACGAHEYHCTTGE-------CIPMSKKCDRNKDCPY-GEDETF--C
T1E9W2_Anopheles_aquasalis             VRAVDC---ESRCESGE-------CLSAGKRCNGHPDCPD-GSDERN--C
P98163_Drosophila_melanogaster         VEALDC---EFRCHSGE-------CLTMNHRCNGRRDCVD-NSDEMN--C
B4Q2Q9_Drosophila_yakuba               VEALDC---EFRCHSGE-------CLTMNHRCNGRRDCVD-NSDEMN--C
B4IGA9_Drosophila_sechellia            VEALDC---EFRCNSGE-------CLTMNHRCNGRRDCVD-NSDEMN--C
K4MUW4_Actias_selene                   TEVARCSESEIYCHKSNI------CVEKFKRCCGVVDCSR-GEDEEG--C
Q1T728_Rhyparobia_maderae              FHPSICSEDKFKCKEDNL------CIPRDFRCNGRRDCPS-GEDELD--C
O76952_Aedes_aegypti                   IDAIDC---EFKCTSGE-------CLTISKRCNGNKDCAD-GSDEKG--C
Q6X0I2_Solenopsis_invicta              ISHHECSKNEYKCSEHNI------CIQRNQLCDGIENCPN-GEDETSE-C
ADE34166.1_Nilaparvata_lugens          SVREACERGEFECGESSVAGIVRHCIAAKKKCDGNKDCPM-GEDEDRSLC
D5KXW4_Nilaparvata_lugens              SVREACERGEFECGESSVAGIVRHCIAAKKKCDGNKDCPM-GEDEDRSLC
BAC02725_Periplaneta_americana         TTPVHCSGEMFKCKTDNF------CIPGRMRCDGKIDCPNGGEDELN--C
ADM34986.1_1_B_tabaci_B                EEIAACGAHEYHCTTGE-------CIPMSKKCDRNKDCPY-GEDETF--C
CAJ19121_Blattella_germanica           FQPVVCSEDKFKCKSDNL------CIPRNFRCNGRKDCQS-GEDELD--C
H2CLV8_Antheraea_pernyi                MEVARCSESEIYCHKSNI------CVEKHKRCNGVVDCSR-GEDEEG--C
XP_008180459_Acyrthosiphon_pisum       TSLGECKSDEYRCYTGE-------CISSHSRCDFIKDCPR-GDDEDVVKC
UPI000252AABD_Apis_florea              NSQTTCNTGEIKCGEHDK------CIKSYQRCDGIIDCPS-GEDESSSFC
UPI0001758603_Tribolium_castaneum      IKRIDCTSHEFFCSGSNS------CIFKKFRCDGERNCPN-GEDETD--C
T1KD20_Tetranychus_urticae             S----CSGDMFKCPGGP-------CIPASRRCDRDADCPD-ISDELD--C
UPI0002063B8E_Apis_mellifera           NSQTTCNTGEIKCGEHDK------CIKSYQRCDGTIDCPS-GEDESSSFC
E2FLQ4_Bombyx_mori                     TEVARCSESEIYCHKSNI------CVEKHKRCNGVVDCSR-GEDEEG--C
```

LDLa

```
Bemisia_tabaci_Asia_1                  PA-----------QCE-TDQFA-CFDGQKCIDAKDRCNMHFDCHDHSDE-
T1E9W2_Anopheles_aquasalis             QPRHGPLEARVSVQCR-WNEFR-CADGRKCISAKRRCDKVHDCADASDE-
P98163_Drosophila_melanogaster         DEEHRR---KPKVLCS-PNQFA-CHSGEQCVDKERRCDNRKDCHDHSDE-
B4Q2Q9_Drosophila_yakuba               DAEHRR---KPKVHCS-PSQFA-CHSGEQCVDKERRCDNRKDCNDHSDE-
B4IGA9_Drosophila_sechellia            DEEHRR---KPKVLCS-PSQFA-CHSGEQCIDKERRCDNRKDCHDRSDE-
K4MUW4_Actias_selene                   THITKK----PESQCD-PNEIL-CYG--LCVAK----DSPSPCSPGKH--
Q1T728_Rhyparobia_maderae              KSD----------KCS-DTEFS-CKNGQ-CIPGDKLCDDEKDCIDGSDE-
O76952_Aedes_aegypti                   DEAGQP----KQLHCQ-YDEFM-CADKSKCIDQTRRCDEHVDCGDGSDE-
Q6X0I2_Solenopsis_invicta              RIKG---------RCK-ENQFM-CKNGD-CIRLKDRCNSRYDCTDQSDE-
ADE34166.1_Nilaparvata_lugens          GLKDFS-----KLHCL-PGQFA-CLDGSRCIPDALLCNWHADCDDHSDET
D5KXW4_Nilaparvata_lugens              GLKDFS-----KLHCL-PGQFA-CLDGSRCIPDALLCNWHADCDDHSDET
BAC02725_Periplaneta_americana         HKV----------NCR-DDQFV-CHNGQ-CISITKKCDGDSDCRDGSDE-
```

```
ADM34986.1_1_B_tabaci_B          PA-----------QCE-TDQFA-CFDGQKCIDAKDRCNMHFDCHDHSDE-
CAJ19121_Blattella_germanica     EAK----------KCL-DSQFT-CKNGQ-CISIEKLCNGERDCLDGSDE-
H2CLV8_Antheraea_pernyi          THITKQ----PESQCE-PNEIL-CYG--LCVAK----DSPSPCSPGKH--
XP_008180459_Acyrthosiphon_pisum LHYTPN-------SCP-QSQFL-CHDKTKCLDKKHMCDSIKDCSDGSDE-
UPI000252AABD_Apis_florea        EDFHWS-------ICKHQDQFR-CKNGE-CISKSKYCNSHYDCSDRSDE-
UPI0001758603_Tribolium_castaneum KELN---------HCP-WNNFQ-CHDGE-CIDERFKCDYKFDCRDKSDE-
T1KD20_Tetranychus_urticae       PPPDCEIYKQSISFNE-NAEFIRCNRTPICIHPEWICDAQNDCLDWSDE-
UPI0002063B8E_Apis_mellifera     EDFHWS-------TCKHQDQFR-CKNGE-CISKSNYCNSHYDCADRSDE-
E2FLQ4_Bombyx_mori               THITKQ----PESQCE-PNEIL-CYG--LCVAK----DSPSPCSPATHSD
```

LDLa

```
Bemisia_tabaci_Asia_1            ANC------QNVTCDQSY----NFLCR-TGECVSHAVLCNNEWNCKDG--
T1E9W2_Anopheles_aquasalis       ANCDGY--VWHGECRES-----QFTCA-NGLCIDANGRCDGYADCRDG--
P98163_Drosophila_melanogaster   QHCEKF--DKSKKCHVH-----QHGCD-NGKCVDSSLVCDGTNDCGDN--
B4Q2Q9_Drosophila_yakuba         QHCEKF--DKTKKCHVH-----QHSCD-NGKCVDSSLVCDGTNDCGDN--
B4IGA9_Drosophila_sechellia      QHCEKF--DKNKKCHVH-----QHGCD-NGKCVDSSLVCDGTNDCGDN--
K4MUW4_Actias_selene             SAVADLTTLPPLKCDWN-----QFTCKESPVCISRSLLCDGAKDCPDG--
Q1T728_Rhyparobia_maderae        KNC--------QKCSEET----DFQCR-TGECIDILDRCDLVPDCRDA--
O76952_Aedes_aegypti             MKCEGY--DRGTGCHEH-----QHACP-DGMCIDVNTLCDGFPDCLDG--
Q6X0I2_Solenopsis_invicta        QNC------EKPKCKSD-----EFQCKFTETCIPKTKMCDSNPDCDDL--
ADE34166.1_Nilaparvata_lugens    LGCDSSRAPEDGRAQPNIPEVCEFRCSTSNECVSEAQRCDLQPDCSDG--
D5KXW4_Nilaparvata_lugens        LGCDSSRAPEDGRAQPNIPEVCEFRCSTSNECVSEAQRCDLQPDCSDG--
BAC02725_Periplaneta_americana   YYC------FEEECNEDL----QFKCR-TGDCIVKSWYCDGSKDCEDG--
ADM34986.1_1_B_tabaci_B          ANC------QNVTCDQSY----NFLCR-IGECVSHAVLCNNEWNCKDG--
CAJ19121_Blattella_germanica     KNC--------EKCEEAI----QFKCS-SGECVDIHDRCDHYPDCTDG--
H2CLV8_Antheraea_pernyi          SAVADLTTPPPLKCDWN-----QFTCKESPVCISRSLLCDGAKDCPDG--
XP_008180459_Acyrthosiphon_pisum MDC-----LHKHTCDSTT----EYQCT-SGECIQRIFLCDGNPDCING--
UPI000252AABD_Apis_florea        EGC----------VKKEY----EFQCH-EGACISKYLVCNGYNDCTDL--
UPI0001758603_Tribolium_castaneum RNCS----IDAKKCPPG-----HFMCK-SGQCINERLVCDGVKDCLEE--
T1KD20_Tetranychus_urticae       ANCAPKYNNSNGTCDPN-----WFQCK-TGLCIPKAWKCDRDNDCVNENT
UPI0002063B8E_Apis_mellifera     EGC------VKKECDSN-----EFQCH-EGACISKYLVCNGYNDCTDL--
E2FLQ4_Bombyx_mori               LTT-----PPPLKCDWI-----QFSCKESPVCISRSLLCDGAKDCPDG--
```

```
Bemisia_tabaci_Asia_1            ---SDE--ENC-----------------TTSTCPSN-EFRCHSGTCIPKN
T1E9W2_Anopheles_aquasalis       ---SDE--LGCKPIAGSNSSSSNGNGNGTTVTCAPH-MFRCNSGQCLPRS
P98163_Drosophila_melanogaster   ---SDE--LLC----------------EATSRCEPG-MFQCGSGSCIAGS
B4Q2Q9_Drosophila_yakuba         ---SDE--MLC----------------EATSRCEQG-MFQCGSGSCIAGT
B4IGA9_Drosophila_sechellia      ---SDE--LLC----------------EATSRCEPG-MFQCGSGSCIAGS
K4MUW4_Actias_selene             ---SDEGPDNC-----------------DTLACFDT-EFMCASGSCILKT
Q1T728_Rhyparobia_maderae        ---SDE--ENC-----------------EGVTCAEG-HYRCVVGVCIPYT
O76952_Aedes_aegypti             ---SDE--VGC---------TDLTNEKSNATTCGPL-MFRCNMGQCIPKW
Q6X0I2_Solenopsis_invicta        ---SDE--EDC-----------------RKVECTSN-EFKCNNGKCIPNT
ADE34166.1_Nilaparvata_lugens    ---SDE--LGC-----------------DAHLCDKTAQFRCRSGACIAAE
D5KXW4_Nilaparvata_lugens        ---SDE--LGC-----------------DAHLCDKTAQFRCRSGACIAAE
BAC02725_Periplaneta_americana   ---SDE--ENC-----------------EEVTCEPS-AFKCALGQCIPEE
ADM34986.1_1_B_tabaci_B          ---SDE--ENC-----------------TTSTCPSS-EFRCHSGTCIPKN
CAJ19121_Blattella_germanica     ---SDE--SNC-----------------ENVSCPPT-DFKCHIGVCVPKY
H2CLV8_Antheraea_pernyi          ---SDEGPDNC-----------------DTLACFDT-EFMCASGSCILKT
XP_008180459_Acyrthosiphon_pisum ---QDE--LNC-----------------LNQSCNNITEFRCNSGNCIPAT
UPI000252AABD_Apis_florea        ---SDE--LNC-----------------NKHKCDND-SFACEIGTCIPKT
UPI0001758603_Tribolium_castaneum ---EDE--ANC-----------------VSTVCKDY-EFRCQSGACIPKN
T1KD20_Tetranychus_urticae       TVTSDE--QDC------------------TYKCHDD-QFQCNNQDCIPAD
UPI0002063B8E_Apis_mellifera     ---SDE--LNC-----------------NKHKCDND-SFACEIGTCIPKT
E2FLQ4_Bombyx_mori               ---SDEGPDNC-----------------DTLACFDT-EFMCASGSCILKT
```

LDLa

```
Bemisia_tabaci_Asia_1            WVCDLDADCPDQSDE--NNCSFSRKEKCT-E--FLC-QSGMCVAQELVCN
T1E9W2_Anopheles_aquasalis       WECDGTPDCTDSSDE-HSQCHNSEKTTCR-PGYHTCLASGVCIEGSLVCD
P98163_Drosophila_melanogaster   WECDGRIDCSDGSDE-HDKCVH---RSCP-PDMQRC-LLGQCLDRSLVCD
B4Q2Q9_Drosophila_yakuba         WECDGRIDCSDGSDE-HDKCAH---RSCP-PDMHRC-LLGQCLDRSLVCD
B4IGA9_Drosophila_sechellia      WECDGRIDCSDGSDE-HDKCVH---RSCP-PDMLRC-LLGQCLDRSLVCD
K4MUW4_Actias_selene             WKCDGDQDCNDASDE--IDCES---VSCK-PGYYQC-RDRECIELKKRCD
Q1T728_Rhyparobia_maderae        WVCDGQSDCVDGSDE-KD-CSP---ITCD-AGSFSC-NNGRCIDRHLLCN
O76952_Aedes_aegypti             WECDGNPDCTDGSDE-HDKCLT--KTDCG-AGFTKC-ALGHCIEDRLLCD
Q6X0I2_Solenopsis_invicta        FVCDNDNDCEDGEDEAAEKCYS--KIACKMPKMFKC-PNGDCISDSLLCN
ADE34166.1_Nilaparvata_lugens    LECNGEMDCRDASDE-HAKCNH--VRTCS-PNQITC-NNGQCIDKELKCN
D5KXW4_Nilaparvata_lugens        LECNGEMDCRDASDE-HAKCNH--VRTCS-PNQITC-NNGQCIDKELKCN
BAC02725_Periplaneta_americana   WVCDGQSDCVDDTDE--QNCAP---PTCG-PGAFSC-GNGRCIDQTLLCN
ADM34986.1_1_B_tabaci_B          WVCDLDADCPDQSDE--NNCSFSRKEKCT-E--FLC-QSGMCVAQELVCN
CAJ19121_Blattella_germanica     WVCDGEPDCIDGTDE--LNCAP---ITCG-PDLFSC-NNGRCVDKKLVCN
```

```
H2CLV8_Antheraea_pernyi              WKCDGDQVCNDASDE--IDCES---VSCK-PGYYQC-RDRECIELKKRCD
XP_008180459_Acyrthosiphon_pisum     WECDGEVDCFDGSDE-HYSCAT---KKCK-DDQFSC-TNGRCISHKFTCN
UPI000252AABD_Apis_florea            WKCDGEVDCPDGSDE-SETCQR---KKCP-SEMFTC-FNGRCIDLILKCN
UPI0001758603_Tribolium_castaneum    WECDHDYDCPDFSDE-HSGCA-----SCD-ASTFTC-NNGKCIDKSFVCD
T1KD20_Tetranychus_urticae           WKCDGHNDCQDNSDENPEVCSS---KVCP-HDYFNC-TSGQCIPLYWVCD
UPI0002063B8E_Apis_mellifera         WKCDGEVDCPDGSDE-SEICQR---KKCS-SEMFTC-FNGRCIDLILKCN
E2FLQ4_Bombyx_mori                   WKCDGDQDCNDASDE--IDCES---VSCK-PGYYQC-RDRECIELKKRCD
```

LDLa

```
Bemisia_tabaci_Asia_1                GQTECDD--GSDE---FNCDEPVPKTANKEDGFIDN---CDEEKE-----
T1E9W2_Anopheles_aquasalis           GNDDCGD--GSDE---ESCSHHEQQDTI-----------CAHPDKANASL
P98163_Drosophila_melanogaster       GHNDCGD--KSDE---LNCGTDSSTMNIS----------CAED-Q-----
B4Q2Q9_Drosophila_yakuba             GHNDCGD--KSDE---LNCGTDSSTVNIS----------CAED-Q-----
B4IGA9_Drosophila_sechellia          GHNDCGD--KSDE---LNCGTDSSTVNIS----------CAED-Q-----
K4MUW4_Actias_selene                 GHQDCFD--YSDE---EECDEPVAVEEPKIHR-------CAEW-E-----
Q1T728_Rhyparobia_maderae            GDNDCGD--YSDE---MECKRPTMVV-------------CQPT-E-----
O76952_Aedes_aegypti                 GNNDCGD--NSDE---LNCKVELEP--------------CVGLEDDNPTK
Q6X0I2_Solenopsis_invicta            GINDCND--GSDE---VHCLSNVTTHLVN----------CSLN-E-----
ADE34166.1_Nilaparvata_lugens        GRNDCDD--ASDE---QSCPQRPIAHIPVFANTGASSPECDTRFE-----
D5KXW4_Nilaparvata_lugens            GRNDCDD--ASDE---QSCPQRPIAHIPVFANTGASSPECDTRFE-----
BAC02725_Periplaneta_americana       NVDDCGD--RSDE---DPCRKPANEEEERLSVIL-----CKEG-E-----
ADM34986.1_1_B_tabaci_B              GQTECDD--GSDE---FNCDEPVPKTANKEDGFIDN---CDEEKE-----
CAJ19121_Blattella_germanica         HNDDCGD--SSDE---ITCKHASSVV-------------CQTT-E-----
H2CLV8_Antheraea_pernyi              GHQDCFD--YSDE---EECDEPVAVEEPKIHR-------CAEW-E-----
XP_008180459_Acyrthosiphon_pisum     GKDDCGD--SSDE---NGCSSNHAYMTKRVSKV------CNEKTE-----
UPI000252AABD_Apis_florea            GISECED--DSDE---KYCNDKSTNNYVN----------CTAD-E-----
UPI0001758603_Tribolium_castaneum    KENDCSD--NSDE---LSCVMENS---------------CDLS-E-----
T1KD20_Tetranychus_urticae           GEIDCHDANGSDENLPEGCNFQK----------------CKPN-E-----
UPI0002063B8E_Apis_mellifera         GISECED--DSDE---KYCNDKNRNNNIN----------CTAD-E-----
E2FLQ4_Bombyx_mori                   GHQDCFD--YSDE---EECDEPVVVEEPKIHR-------CAER-E-----
```

LDLa

```
Bemisia_tabaci_Asia_1                FMCEPG-----KCINLIFKCNGAKDCENGADE-LNC-IG-CE--Q-FTCN
T1E9W2_Anopheles_aquasalis           FYCTRSS----HCFDASARCNGTAECPHGEDE-TDC-PG-CAHQE-FACA
P98163_Drosophila_melanogaster       YQCTSNLK---ICLPSTVRCNGTTECPRGEDE-ADC-GDVCSIYE-FKCR
B4Q2Q9_Drosophila_yakuba             FQCTSNLK---ICLPSAVRCNGTTECPRGEDE-ADC-GDVCSIYE-FKCR
B4IGA9_Drosophila_sechellia          FQCTSNLK---ICLPSAVRCNGTTECPRGEDE-ADC-GDVCSIYE-FKCR
K4MUW4_Actias_selene                 YSCERNRS---ICLPITARCNMKTDCPGGTDE-IGC-DYRCTPHGMFGCK
Q1T728_Rhyparobia_maderae            IPCLSHNKTVISCVPSSARCNDVAECPLGDDE-RAC-DK-CLDFQ-FRCS
O76952_Aedes_aegypti                 YLCPRSG----KCLDIAVRCNGTAECPDGEDE-AGC-SN-CGLQE-FQCK
Q6X0I2_Solenopsis_invicta            YRCLGTD----ICLPKNVRCDGKNDCPQSDDE-QNC-TY-CFENE-FACD
ADE34166.1_Nilaparvata_lugens        FECERG-----HCIPSSARCNHTSECSNGRDE-LNC-MG-CHHDQ-FQCK
D5KXW4_Nilaparvata_lugens            FECERG-----HCIPSSARCNHTSECSNGRDE-LNC-MG-CHHDQ-FQCK
BAC02725_Periplaneta_americana       YTCHPHGKNVTICLPSSGRCNGTAECPLGDDE-RGC-G--CQDFQ-FTCY
ADM34986.1_1_B_tabaci_B              FMCEPG-----KCINLIFKCNGVKDCENGADE-LNC-IG-CE--Q-FTCN
CAJ19121_Blattella_germanica         ITCTSHNKSVISCVPMSARCNDIPDCPLGDDE-RGC-EK-CMDFQ-FKCN
H2CLV8_Antheraea_pernyi              YSCERNRS---ICLPITARCNMKTDCPGGTDE-IGC-DYRCTPHGMFGCK
XP_008180459_Acyrthosiphon_pisum     FECENTMG---HCIPIKARCNGTSECKHLEDE-LNC-G--CQKSNFFECQ
UPI000252AABD_Apis_florea            YKCFDSD----LCLPKQFRCDGIKNCPKNDDE-RDC-AR-CNEAE-YVCE
UPI0001758603_Tribolium_castaneum    FSCSLHTH---ICLPDSARCNGTSECPHHEDE-QNC-SN-CQVDE-FSCN
T1KD20_Tetranychus_urticae           FSCKNH-----QCILKKFYCDGDDDCGDKSDEPPNCRSSKCLSDQ-FECM
UPI0002063B8E_Apis_mellifera         YKCFDSD----LCIPKRFRCDGIKNCPKNDDE-RDC-AR-CNEAE-YVCE
E2FLQ4_Bombyx_mori                   YSCERNRS---ICLPITARCNMKTDCPGGTNE-IGC-DYRCTPHGMFGCK
```

LDLa

```
Bemisia_tabaci_Asia_1                NGK-CITYDLVCNDDDDCGDSSDERPLNSCPDSKENPAIVPAHIP-NVCH
T1E9W2_Anopheles_aquasalis           NGQ-CIPREWRCDKELDCADGSDEWNCTVTDRGTHESPQH------ILCG
P98163_Drosophila_melanogaster       SGRECIRREFRCDGQKDCGDGSDELSCELEKGHHNQSQIQPWSTSSRSCR
B4Q2Q9_Drosophila_yakuba             SGRQCIRREFRCDGQKDCGDASDELSCELEKGNRNQSQIQPWSTSSRACR
B4IGA9_Drosophila_sechellia          SGRECIRREFRCDGQKDCGDGSDELSCELEKGNHNQSQIQPWSTSSLACR
K4MUW4_Actias_selene                 QQIRCLAMNRVCDGNKECDDGSDETPDACALVNRTSHLYPVMLYPAAECR
Q1T728_Rhyparobia_maderae            NGR-CIPQEWTCDKTDDCDDGSDEDPVLCHEHSTQQST------P-GPCR
O76952_Aedes_aegypti                 SGK-CIRKEWRCDKEVDCDDGSDEVDCVNGTAAEHLEVH-------VACG
Q6X0I2_Solenopsis_invicta            NKR-CIPELWVCDKANDCGDNSDEKNCDGSKRNFIES---------NEC-
ADE34166.1_Nilaparvata_lugens        NER-CIYHTWVCDKKNDCGDNSDEEALCKTRKGNADQSVATKSD-TRCF
D5KXW4_Nilaparvata_lugens            NER-CIYHTWVCDKKNDCGDNSDEEALCKTRKGNADQSVATKSD-TRCF
BAC02725_Periplaneta_americana       NGK-CIPSEWVCDGINDCGDGSDENNARCQLPSSVGTP--------GPCT
ADM34986.1_1_B_tabaci_B              NGK-CITYDLVCNDDDDCGDSSDERPLNSCPDSKENPAIVPAHIP-NVCH
CAJ19121_Blattella_germanica         DGR-CIPFEWTCDGTKDCADGSDENQMHCHSQSVETGT------P-GPCT
H2CLV8_Antheraea_pernyi              QQIRCLAMNRVCDGNKEYDNGSDETPDACALVNRTSHLYPVMLYPAAECR
XP_008180459_Acyrthosiphon_pisum     NKR-CVLKDWLCDKHDDCGDGSDESQKACDMLSEHLSNSVVSS---KDCD
```

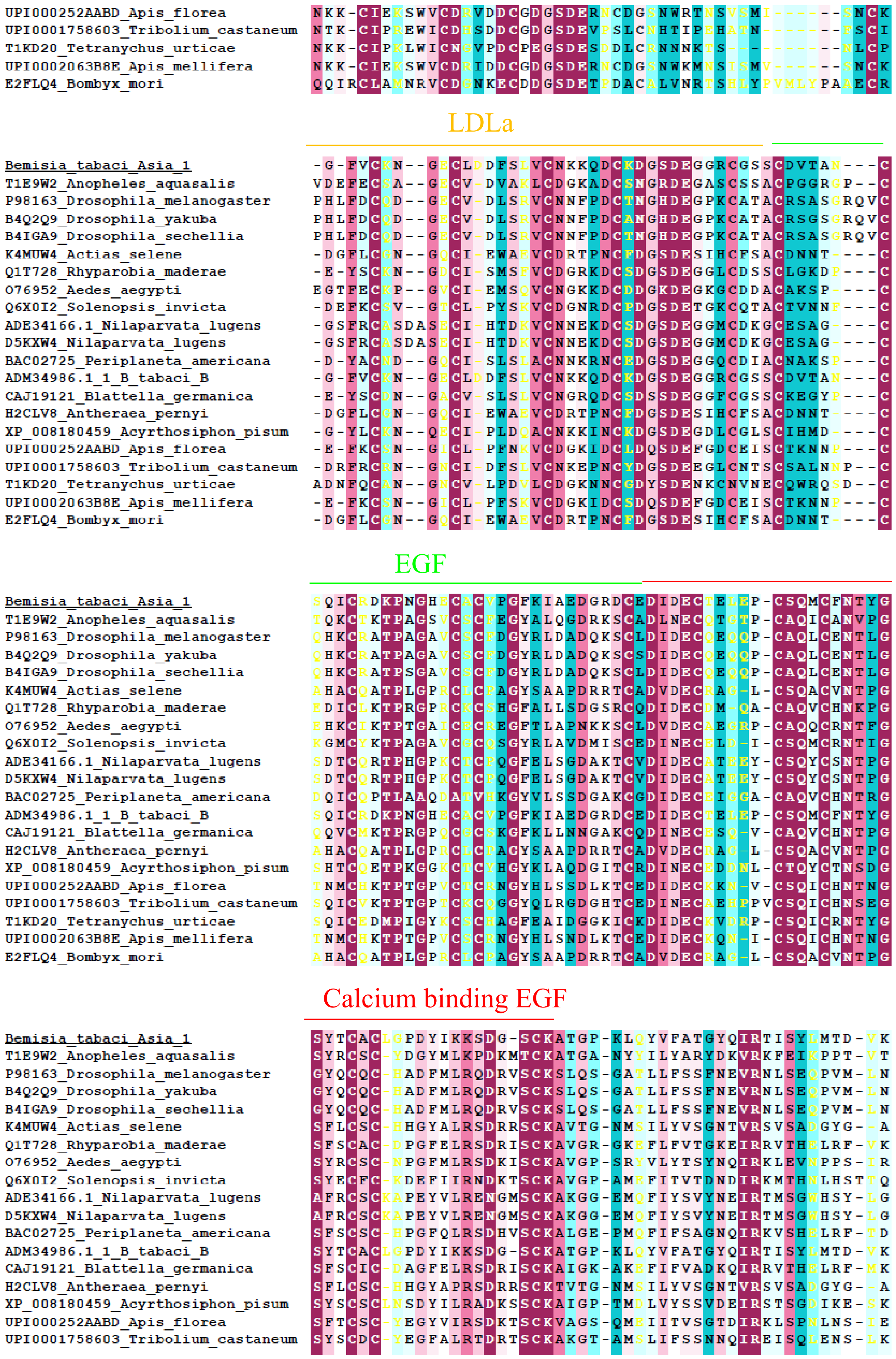

UPI000252AABD_Apis_florea
UPI0001758603_Tribolium_castaneum
T1KD20_Tetranychus_urticae
UPI0002063B8E_Apis_mellifera
E2FLQ4_Bombyx_mori
LDLa
Bemisia_tabaci_Asia_1
T1E9W2_Anopheles_aquasalis
P98163_Drosophila_melanogaster
B4Q2Q9_Drosophila_yakuba
B4IGA9_Drosophila_sechellia
K4MUW4_Actias_selene
Q1T728_Rhyparobia_maderae
O76952_Aedes_aegypti
Q6X0I2_Solenopsis_invicta
ADE34166.1_Nilaparvata_lugens
D5KXW4_Nilaparvata_lugens
BAC02725_Periplaneta_americana
ADM34986.1_1_B_tabaci_B
CAJ19121_Blattella_germanica
H2CLV8_Antheraea_pernyi
XP_008180459_Acyrthosiphon_pisum
UPI000252AABD_Apis_florea
UPI0001758603_Tribolium_castaneum
T1KD20_Tetranychus_urticae
UPI0002063B8E_Apis_mellifera
E2FLQ4_Bombyx_mori
EGF
Bemisia_tabaci_Asia_1
T1E9W2_Anopheles_aquasalis
P98163_Drosophila_melanogaster
B4Q2Q9_Drosophila_yakuba
B4IGA9_Drosophila_sechellia
K4MUW4_Actias_selene
Q1T728_Rhyparobia_maderae
O76952_Aedes_aegypti
Q6X0I2_Solenopsis_invicta
ADE34166.1_Nilaparvata_lugens
D5KXW4_Nilaparvata_lugens
BAC02725_Periplaneta_americana
ADM34986.1_1_B_tabaci_B
CAJ19121_Blattella_germanica
H2CLV8_Antheraea_pernyi
XP_008180459_Acyrthosiphon_pisum
UPI000252AABD_Apis_florea
UPI0001758603_Tribolium_castaneum
T1KD20_Tetranychus_urticae
UPI0002063B8E_Apis_mellifera
E2FLQ4_Bombyx_mori
Calcium binding EGF
Bemisia_tabaci_Asia_1
T1E9W2_Anopheles_aquasalis
P98163_Drosophila_melanogaster
B4Q2Q9_Drosophila_yakuba
B4IGA9_Drosophila_sechellia
K4MUW4_Actias_selene
Q1T728_Rhyparobia_maderae
O76952_Aedes_aegypti
Q6X0I2_Solenopsis_invicta
ADE34166.1_Nilaparvata_lugens
D5KXW4_Nilaparvata_lugens
BAC02725_Periplaneta_americana
ADM34986.1_1_B_tabaci_B
CAJ19121_Blattella_germanica
H2CLV8_Antheraea_pernyi
XP_008180459_Acyrthosiphon_pisum
UPI000252AABD_Apis_florea
UPI0001758603_Tribolium_castaneum

```
T1KD20_Tetranychus_urticae            SYSCSC-VPGYYSANNGSSCKANSDIEPYLLFADRYSIAYSDLKGHN-LR
UPI0002063B8E_Apis_mellifera          SFICSC-YEGYVIRSDKTSCKVAGS-QMEIIIVSDTHIRKLSSNLNS-IE
E2FLQ4_Bombyx_mori                    SFLCSC-HHGYALRSDRRSCKAVTG-NMSILYVSGNTVRSVSADGYG--A


Bemisia_tabaci_Asia_1                 VAYYSADL--EVSGFDVNMRTEHVYWS-SENKGVITKMSLTHRHEPKHF-
T1E9W2_Anopheles_aquasalis            TLARATSS--RITSMDANMHQGKLYYT-AENTSAIFETDLM-GNESRIV-
P98163_Drosophila_melanogaster        VAWSANDS--RITGFDLAMHRQMGYFS-AEDEGIVYQIDL-----QTKVI-
B4Q2Q9_Drosophila_yakuba              VAWSANDS--RITGFDVDMHRQMGYFS-AEDEGIVYQVDL-----QTKLI-
B4IGA9_Drosophila_sechellia           VAWSANDS--RITGFDLDMHRQMGYFS-VEDEGIVYQIDL-----QTKVI-
K4MUW4_Actias_selene                  IEYSDPDL-GDITDLDFNVRTKRLYVT-STESGKLIELNV---THDVVA-
Q1T728_Rhyparobia_maderae             VAYPDNDF--QVTGLDADSQLGMVYWS-VGAANAIFSMSL--IGGKKTQ-
O76952_Aedes_aegypti                  ILMQANGS--RITSMDVDIRRQMLYFT-DEYNPVIYEHDM--ERNTTHV-
Q6X0I2_Solenopsis_invicta             LLFPLMGV--RVSGLDVNAVSDSVYWS-NDEFGTIKKLNI--RTNEIVT-
ADE34166.1_Nilaparvata_lugens         IVHSDPDYRARVAGLTADVARKHVYWT-TTTNDSVFQISM----DNRRM-
D5KXW4_Nilaparvata_lugens             IVHSDPDYRARVAGLTADVARKHVYWT-TTTNDSVFQISM----DNRRM-
BAC02725_Periplaneta_americana        VVYPEAEL--KVTGLDVDSASNEVYWS-TDVTSTIYRLSL--RGGEKAY-
ADM34986.1_1_B_tabaci_B               VAYYSADL--EVSGFDVNMRTEHVYWS-SENKGVITKMSLTHRHEPKHF-
CAJ19121_Blattella_germanica          IAYLDYDF--KVTGLDVDSKLGMVYWS-SDETNTIYRMSL--IGGTKAY-
H2CLV8_Antheraea_pernyi               IEYSDPDL-GDITDLDFNVRTKRLYVT-STESGKLIELNV---THDVVA-
XP_008180459_Acyrthosiphon_pisum      LIFSMPGM--TISSLDIDIRRNLIYWT-SKQAGVLICMDM--QQQHKFY-
UPI000252AABD_Apis_florea             LIYKEVDF--EINGIDVNTREDTIYWS-NEMLGMINKIHV--KTKERKT-
UPI0001758603_Tribolium_castaneum     MLYSEETP--RITSLDVGLTSGAIYFT-VENSNAILKINK--GDTKREY-
T1KD20_Tetranychus_urticae            LKVHNLT---NAIGLDFDWEEKCIYWSEVSQAGSSINKACSKDHNDTLLP
UPI0002063B8E_Apis_mellifera          FIYKEIDF--EINGIDVNTREDTIYWS-NEMLGMINKIHV--KTKERKT-
E2FLQ4_Bombyx_mori                    IEYSDPDL-GDITDLDFNVRTKRLYVT-STESGKLIELNV---THDVVA-
```

LDLb

```
Bemisia_tabaci_Asia_1                 ------ITGLR--RPSELAVDWITHNLYFVQ---ARNTINVCNFHLERCA
T1E9W2_Anopheles_aquasalis            ------LRSVG--KPEKLAVDWVANNVYFIDG--SEPSIKVCSIGRANCA
P98163_Drosophila_melanogaster        ------VRALGLPAPTKLSVDWVTGNVYVLS---GAQEIQACSFVGRMCG
B4Q2Q9_Drosophila_yakuba              ------TRALGLPTPTKLSVDWVTGNVYVLS---GAQEIQACSFEGRMCG
B4IGA9_Drosophila_sechellia           ------VRALGLPAPTKLSVDWATGNVYVLS---GAQDIHACSFVGRMCG
K4MUW4_Actias_selene                  ------VTNVG--RPTRVAVDWVTGNVYFADSTPGASCVRVCDVTRRRCA
Q1T728_Rhyparobia_maderae             ------MKGIG--NPADIAVDWITHNVYYIDKD-AIQTIKVCNLDDQLHA
O76952_Aedes_aegypti                  ------LYNVG--HPEHLAVDWITGNVYFYDR--SEPSIKLCSVQRQLCS
Q6X0I2_Solenopsis_invicta             ------VKIVE--HPQALAVDWITGNVYVNDNS-HLNTIKVCNLEKGKCA
ADE34166.1_Nilaparvata_lugens         ------IRSAHIIRPSRLAIDWITGNVYVVE---AATQITAVNFEKRTHA
D5KXW4_Nilaparvata_lugens             ------IRSAHIIRPSRLAIDWITGNVYVVE---AATQITAVNFEKRTHA
BAC02725_Periplaneta_americana        ------ATGIG--TPGDIAVDWISRNVYYVDKS-TPQAIRACNLDEHRCA
ADM34986.1_1_B_tabaci_B               ------ITGLR--RPSELAVDWITHNLYFVQ---ARNIINVCNFHLERCA
CAJ19121_Blattella_germanica          ------LTGIG--SPTDIALDWITQNVYYVDKD-TIQSIKVCNLDSQQHA
H2CLV8_Antheraea_pernyi               ------VTNVG--RPTRVAVDWVTGNVYFADSTPGASCVRVCDVTRRRCA
XP_008180459_Acyrthosiphon_pisum      ------MTDLL--HPTLVRIDWLTENVYFVQ---NFKDIVVCYLNAKRCA
UPI000252AABD_Apis_florea             ------VTGLG--RPEALAVDWITDNVYFNDND-YTSSIQVCNLEQKKCA
UPI0001758603_Tribolium_castaneum     ------LENVG--QPCKITLDWRTNNIYYFNNAPTTKSIGLCNFNDKTCA
T1KD20_Tetranychus_urticae            PSSVVKIHTVHIQSPDGLAVDWFAKNLYWSDK--GRRTIEVSKLDGSFRK
UPI0002063B8E_Apis_mellifera          ------VTGLG--RPEALAVDWITDNVYFNNND-YSSSIEVCNLEQKKCA
E2FLQ4_Bombyx_mori                    ------VTNVG--RPTRVAVDWVTGNVYFADSTPGASCVRVCDVTRRRCA
```

LDLb

```
Bemisia_tabaci_Asia_1                 QILTAESGLEINSLAVDPVRGVLFWSETSRIVWNMPKSTIRRADMNGKNI
T1E9W2_Anopheles_aquasalis            RLVTFMRQNFLKALCVDPVNST----------------------------
P98163_Drosophila_melanogaster        RIVHVKSPRHVKHLAVDGYHARIFYIVIRTEGYGQTSSEIHMARLDGSRR
B4Q2Q9_Drosophila_yakuba              RIVHVKSPKHVKHLAVDGYHGRIFYIVIRTEGYGQTSSEIHMARLDGSRR
B4IGA9_Drosophila_sechellia           RIAHVKSPRHVKHLAVDGYHARIFYIVIRTEGYGQTSSEIHMARLDGSRR
K4MUW4_Actias_selene                  RLQKIPSDATVKALIVEPASRRMFYC------VQRGHESVVWSASLSGRSA
Q1T728_Rhyparobia_maderae             KVMDVSNGFSASRLAVDPFHGFLFWVEVNKWRIDVPASVLLRADMNGENR
O76952_Aedes_aegypti                  RIITFASQVFVKAVVVDPVNRILFYSLMHFWIFQVPHSIIYRADMDGQNQ
Q6X0I2_Solenopsis_invicta             TLVKIQDKMKVASVIVDSINRWLFWAEISLEA-DHPTSKICRTDMTGADM
ADE34166.1_Nilaparvata_lugens         RLYKSDPAKDIEALAVDPVTRTMFWSE--RLAHKIQFSAIYRADTSGSGL
D5KXW4_Nilaparvata_lugens             RLYKSDPAKDIEALAVDPVTRTMFWSE--RLAHKIQFSAIYRADTSGSGL
BAC02725_Periplaneta_americana        KVLVIEHGFSVPKIAVDPIAGFIFWPEVTKWVFGEPSTDLFRSELTGRHK
ADM34986.1_1_B_tabaci_B               QILTAESGLEINSLAVDPVRGVLFWSETSRIVWNMPKSSIRRADMNGKNI
CAJ19121_Blattella_germanica          TVVHVDSGYSASRIAVDPFAGFIFWIEISTWNIEIPKSILIRADMSGENR
H2CLV8_Antheraea_pernyi               RLQKIPSDATVKALIVEPASRRMFYC------VQRGHESVVWSASLSGRSA
XP_008180459_Acyrthosiphon_pisum      TIYSANIHTTIMAFEIDPRSGVMFWSETIWAVFSTPTTIIRKSDCSGYNV
UPI000252AABD_Apis_florea             KIVSIAGKNRVISIAVEPKKGWLFWSQTSWAFYDRPMSKIYRSNTMGNNA
UPI0001758603_Tribolium_castaneum     NLIPVDTHRQVSALVVDPINNVLFYSLVSWWIFNSPSYIVYKTNLDGTGG
T1KD20_Tetranychus_urticae            ILLKDDLQLP-RAIALDPSEGYMYWTD-----WGENPY-IGKSGMDGSNS
UPI0002063B8E_Apis_mellifera          KIVSIAGKNHVISIAVEPRKGWLFWSQTSWAFYDRPMSKIYRSNTMGNNA
```

```
E2FLQ4_Bombyx_mori                  RLQKIPSDATVKALIVEPASRRMFYC-----VQRGHESVVWSASLSGRSA
```

LDLb

```
Bemisia_tabaci_Asia_1               ETIVSANVSYALDLALDPILNHVYWVDKTLKVIERANYDGT-----RRRV
T1E9W2_Anopheles_aquasalis          --------------------------------------------------
P98163_Drosophila_melanogaster      DMLLQRSESFMTALTTDPHQQLLYFVDQHMRTLERISYRLKTGPMRRPEI
B4Q2Q9_Drosophila_yakuba            DMLLQRGESFMTALTTDPHQQLLYFVDQHTRTLERISYRFKMGPLRRPEI
B4IGA9_Drosophila_sechellia         DMLVQRSESFMTALTTDPHQQLLYFVDQHTRTLERISYRLKTGPMRRPEI
K4MUW4_Actias_selene                LDLLH--VTQCSGLAADSFTRRLYVAETAPPHIMVVDFDGK-----NPKK
Q1T728_Rhyparobia_maderae           MEVASDHMMLVNGIALDFIRKKIYIADEHTNTIECMSYSGS-----DRHL
O76952_Aedes_aegypti                L-VITKDVSHVTSLQVDTENKLLYIADISSRTINALDYEGK----KLRTV
Q6X0I2_Solenopsis_invicta           K-IIASDLGFVRGMTIDHVKSKLYWSDDFYKTVESSNFDGS-----QRKV
ADE34166.1_Nilaparvata_lugens       LEIVSTNLKQVSDIFVDSFHAHIYWVDSQTRKVERASFDGS-----NRQE
D5KXW4_Nilaparvata_lugens           LEIVSTNLKQVSDIFVDSFHAHIYWVDSQTRKVERASFDGS-----NRQE
BAC02725_Periplaneta_americana      TMIETNNLMLVNGLTLDIVRQRIYFADQHKRTIECMDYNGE-----DRHI
ADM34986.1_1_B_tabaci_B             ETIVSANVSYALDLALDPILNHVYWVDKTLKVIERANYDGT-----RRRV
CAJ19121_Blattella_germanica        KTLVNEHMMLVYGIALDVVRKRIFIADEHQNTIESMGYSGE-----DRHV
H2CLV8_Antheraea_pernyi             LDLLH--VTQCSGLAADSFTRRLYVAETAPPHIMVVDFDGK-----NPKK
XP_008180459_Acyrthosiphon_pisum    KTIIHQGLQYVTDLAIDPIKHMVYWVDQVNSTIERSNYDGT-----KITV
UPI000252AABD_Apis_florea           TAIVHLDLGLVLALTIDYARSKLYWSDTHFKNIESSNLDGS-----NRAV
UPI0001758603_Tribolium_castaneum   QELIKTSPGYITGLSYDLYKKELYFISQHQ--LSKIKYDGS-----DRTV
T1KD20_Tetranychus_urticae          FRLINESLGWPNALTIDYITRELFWADAKEDYISVSNLDGS-----RRHI
UPI0002063B8E_Apis_mellifera        TAIVHLDLGLVLALTIDYTRSKLYWSDTHFKNIESSNLDGS-----NRAV
E2FLQ4_Bombyx_mori                  LDLLH--VTQCSGLAADSFTRRLYVAETAPPHIMVVDFDGK-----NPKK
```

```
Bemisia_tabaci_Asia_1               IL---TSKFHPK--SVALFDGSIYWSVES-----------SGSPITKCAL
T1E9W2_Anopheles_aquasalis          --------------------------------------------------
P98163_Drosophila_melanogaster      MLQKSNALMHPS--GLSVYENNAFIVNLG-----------SVE-AVQCAL
B4Q2Q9_Drosophila_yakuba            MLQKSNALMHPS--GLSVYENNAFIVNLG-----------SVE-AVQCAL
B4IGA9_Drosophila_sechellia         MLQKSNALMHPS--GLSVYENNAFIVNLG-----------SVE-AVQCAL
K4MUW4_Actias_selene                ILTERPQLQAPH--ALALFEDHIYYLVG------------DSYRLGRCLL
Q1T728_Rhyparobia_maderae           IV-SSEHVKHPI--NLALFEGTLYWLTAG-----------TGH-ITSYKL
O76952_Aedes_aegypti                IENQNLAVSKPI--GIMIYENQALVLNM------------ASSTVGQCKL
Q6X0I2_Solenopsis_invicta           VL--TLNMNHAL--SISIFEQSLYFLS-------------SDNLLSSCKM
ADE34166.1_Nilaparvata_lugens       VF---TSPAVPT--DITLFEDYMYVLVQADSASENIDMIETGN-VWRCGL
D5KXW4_Nilaparvata_lugens           VF---TSPAVPT--DITLFEDYMYVLVQADSASENIDMIETGN-VWRCGL
BAC02725_Periplaneta_americana      IV-HNEHVQNPI--DMALFEGTLYWLTAG-----------TGQ-LTSYKL
ADM34986.1_1_B_tabaci_B             IL---TSKFHPK--SVALFDGSIYWSVES-----------SGSPITKCAL
CAJ19121_Blattella_germanica        VVDSNEHVQKPI--NLALFEGTLYWLTSG-----------TGQ-LTSYKL
H2CLV8_Antheraea_pernyi             ILTERPQLQAPH--ALALFEDHIYYLVG------------DSYRLGRCLL
XP_008180459_Acyrthosiphon_pisum    LV--NSIEHLPE--KISLYEDKLFWTNHE-----------TGLSIFKCKV
UPI000252AABD_Apis_florea           VL--NTDIHQAM--SINIYEDSLYWLMGG-----------TGI-IRKCKL
UPI0001758603_Tribolium_castaneum   LL---SNLTQSV--GLNFFENHLYFK--------------SGNFMQKCRL
T1KD20_Tetranychus_urticae          IVHKANSRFISRVFAITLFEDYLYWTDW------------ERHTIAMCHK
UPI0002063B8E_Apis_mellifera        VL--NTGIHQAM--SINIYEDFLYWLMGG-----------TGI-IRKCKL
E2FLQ4_Bombyx_mori                  ILTERPQLQAPH--ALALFEDHIYYLVG------------DSYRLGRCLL
```

```
Bemisia_tabaci_Asia_1               QGLSTESYSCNQIPIKVVDPITHFTLMQPALQ--R-NISNACRNM--ECS
T1E9W2_Anopheles_aquasalis          -----------------------------------------CSSR--YCT
P98163_Drosophila_melanogaster      YGSRIC--HKISINVLNAQ---DIVVAGRSRQ--PQKASHPCAHA--HCH
B4Q2Q9_Drosophila_yakuba            YGSRTC--HKISLNVLNAQ---DIVVAGKSRQ--PQKLSHPCGHA--HCH
B4IGA9_Drosophila_sechellia         YGSRIC--HKISLNVLNAQ---DIVVAGRSRQ--PEKASHPCAHA--HCH
K4MUW4_Actias_selene                HGPKNC-----ETYIYRVFEANTFVIRHESIQ--RDDLVNECAGH--DCS
Q1T728_Rhyparobia_maderae           YGPPEMRFNKEQLYSYGTE---HFTILQPSMQ--P-LVANPCANH--TCS
O76952_Aedes_aegypti                YGDFEC-----RLMELNVHNSNQLLIVQESRQ--P-EAENVCDTKQINCS
Q6X0I2_Solenopsis_invicta           YGKRSC----EHVNIGANNVFRLFSILHISRQ--V-PFANPCDAE--YCD
ADE34166.1_Nilaparvata_lugens       YGAAYQKCELFRIHPKHFTVPYHFEIMHPGLQ--L-KGHNDCRNST-DCQ
D5KXW4_Nilaparvata_lugens           YGAAYQKCELFRIHPKHFTVPYHFEIMHPGLQ--L-KGHNDCRNST-DCQ
BAC02725_Periplaneta_americana      YGPHERRIGKLQLYIYSSD---QFTILQQAIQ--P-AAVNPCANH--SCS
ADM34986.1_1_B_tabaci_B             QGLSTESYSCNQIPIKVVDPITHFTLMQPALQ--R-NISNACRNM--ECS
CAJ19121_Blattella_germanica        YGPPERPFDKHQLYSYGTE---HFAILQASMQ--P-IIVNPCANH--TCD
H2CLV8_Antheraea_pernyi             HGPKNC-----ETYIYRVFDANTFVIRHESIQ--RDDLVNECAAH--DCS
XP_008180459_Acyrthosiphon_pisum    LGSENV--HCESVPVQVFATIETFTISQQAKQ--R-NGSNVCIDV--DCD
UPI000252AABD_Apis_florea           YGDKLC----TTISIGTSNINKYFIILHTIRQ--P-IGKNVCEKY--KCN
UPI0001758603_Tribolium_castaneum   YSPITC-----ETFGLKGD---VFAIAQESRQ--PEFGNNPCQNH--TCN
T1KD20_Tetranychus_urticae          YHCNSS-----SKVLSLSERPMDIHIYHPFRQINP-KFENPCNLL--NCS
UPI0002063B8E_Apis_mellifera        YGDKSC----TTIPIGTSNINKYFIILHTIRQ--P-IGKNVCEKY--KCN
E2FLQ4_Bombyx_mori                  HGPKNC-----ETYIYRVFDANTFVIRHESIQ--RDDLVNECAGH--DCS
```

```
Bemisia_tabaci_Asia_1                ---HMC--------------------VLSSTL----PSCI----------
T1E9W2_Anopheles_aquasalis           ---RGV------------------------------QDTA----------
P98163_Drosophila_melanogaster       ---GLC--------------------LQADYG----YECM----------
B4Q2Q9_Drosophila_yakuba             ---GLC--------------------LQADYG----YECM----------
B4IGA9_Drosophila_sechellia          ---GLC--------------------LQADYG----YECM----------
K4MUW4_Actias_selene                 ---NVC--------------------VLEKA-----PVCV----------
Q1T728_Rhyparobia_maderae            ---EMC--------------------VLSPGR---NPSCL----------
O76952_Aedes_aegypti                 ---HVC--------------------VPGADG---HGVCI----------
Q6X0I2_Solenopsis_invicta            ---YMC--------------------VLKKEN----ATCI----------
ADE34166.1_Nilaparvata_lugens        SAGGMC--------------------LLRNHKLRPSAVCV----------
D5KXW4_Nilaparvata_lugens            SAGGMC--------------------LLRNHKLRPSAVCV----------
BAC02725_Periplaneta_americana       ---ELC--------------------VMNPGG---TPSCL----------
ADM34986.1_1_B_tabaci_B              ---HMC--------------------VLSSTL----PSCI----------
CAJ19121_Blattella_germanica         ---EMC--------------------VLSPSG---YPVCL----------
H2CLV8_Antheraea_pernyi              ---NVC--------------------VLEKA-----PVCV----------
XP_008180459_Acyrthosiphon_pisum     ---FIC--------------------TSGSKG----PMCV----------
UPI000252AABD_Apis_florea            ---YMC--------------------VLGNNG----PACI----------
UPI0001758603_Tribolium_castaneum    ---QLC--------------------IPAKPQ----YRCL----------
T1KD20_Tetranychus_urticae           ---GLCLLRPGLTHQAVGVCSCPDDHVLGPNG----RSCISNCSDSQFRC
UPI0002063B8E_Apis_mellifera         ---YMC--------------------VLGNNG----PACI----------
E2FLQ4_Bombyx_mori                   ---NVC--------------------VLEKA-----PVCV----------
```

```
Bemisia_tabaci_Asia_1                ----------------------------------------CR-NGKIVPP
T1E9W2_Anopheles_aquasalis           ----------------------------------------CHLSGE----
P98163_Drosophila_melanogaster       ----------------------------------------CG-N-RLVAE
B4Q2Q9_Drosophila_yakuba             ----------------------------------------CA-N-RLVAE
B4IGA9_Drosophila_sechellia          ----------------------------------------CG-N-RLVAE
K4MUW4_Actias_selene                 ----------------------------------------CD-DGHVRDD
Q1T728_Rhyparobia_maderae            ----------------------------------------CS-DSTTRSM
O76952_Aedes_aegypti                 ----------------------------------------CH-NGERIHG
Q6X0I2_Solenopsis_invicta            ----------------------------------------CS-DGESIES
ADE34166.1_Nilaparvata_lugens        ----------------------------------------CA-DGTRMRR
D5KXW4_Nilaparvata_lugens            ----------------------------------------CA-DGTRMRR
BAC02725_Periplaneta_americana       ----------------------------------------CS-GGQVVEM
ADM34986.1_1_B_tabaci_B              ----------------------------------------CR-NGKIVPP
CAJ19121_Blattella_germanica         ----------------------------------------CS-DGTIVEM
H2CLV8_Antheraea_pernyi              ----------------------------------------CD-DGHVRDD
XP_008180459_Acyrthosiphon_pisum     ----------------------------------------CG-DGSQVEP
UPI000252AABD_Apis_florea            ----------------------------------------CP-NGYSKDS
UPI0001758603_Tribolium_castaneum    ----------------------------------------CE-NGKFVEP
T1KD20_Tetranychus_urticae           KRTMKCISKKWVCDKHDDCKDGSDEPEQCKLSSCETGFFTCR-NGSCIPV
UPI0002063B8E_Apis_mellifera         ----------------------------------------CP-DGYPKDS
E2FLQ4_Bombyx_mori                   ----------------------------------------CD-DGHVRDD
```

```
Bemisia_tabaci_Asia_1                KTACTDSNYMPE--------------------------------------
T1E9W2_Anopheles_aquasalis           --------------------------------------------------
P98163_Drosophila_melanogaster       GERCPHGSGNEVAVLG----------------------------------
B4Q2Q9_Drosophila_yakuba             GERCPHGSGNEVAVLG----------------------------------
B4IGA9_Drosophila_sechellia          GERCPHGSGNEVAVLG----------------------------------
K4MUW4_Actias_selene                 GN-CDPSSKNELPLFN----------------------------------
Q1T728_Rhyparobia_maderae            GEPCPAREEIYERSSI----------------------------------
O76952_Aedes_aegypti                 TDICPQTSNVMTPLVK----------------------------------
Q6X0I2_Solenopsis_invicta            NSTCNIKNDL----------------------------------------
ADE34166.1_Nilaparvata_lugens        NSICEASSATIFDYDD----------------------------------
D5KXW4_Nilaparvata_lugens            NSICEASSATIFDYDD----------------------------------
BAC02725_Periplaneta_americana       GELCPTSEVGEGP-------------------------------------
ADM34986.1_1_B_tabaci_B              KTACTDSNYMPE--------------------------------------
CAJ19121_Blattella_germanica         GKSCP-KEELHDGSRS----------------------------------
H2CLV8_Antheraea_pernyi              GN-CDPSSKNELPLFN----------------------------------
XP_008180459_Acyrthosiphon_pisum     GNICDDAGHRKLPVFK----------------------------------
UPI000252AABD_Apis_florea            KNICSENMNTRLIFNS----------------------------------
UPI0001758603_Tribolium_castaneum    EEDCD--TLMREANDD----------------------------------
T1KD20_Tetranychus_urticae           NQFCDGISQCSDGSDEECGHNLIDRCAECTIEGCKSICLNGGSCYMDEQQ
UPI0002063B8E_Apis_mellifera         KNTCLENMNTKLIFNS----------------------------------
E2FLQ4_Bombyx_mori                   GS-CDSSNKNELPLFN----------------------------------
```

```
Bemisia_tabaci_Asia_1              --------------------------------------------------
T1E9W2_Anopheles_aquasalis         --------------------------------------------------
P98163_Drosophila_melanogaster     --------------------------------------------------
B4Q2Q9_Drosophila_yakuba           --------------------------------------------------
B4IGA9_Drosophila_sechellia        --------------------------------------------------
K4MUW4_Actias_selene               --------------------------------------------------
Q1T728_Rhyparobia_maderae          --------------------------------------------------
O76952_Aedes_aegypti               --------------------------------------------------
Q6X0I2_Solenopsis_invicta          --------------------------------------------------
ADE34166.1_Nilaparvata_lugens      --------------------------------------------------
D5KXW4_Nilaparvata_lugens          --------------------------------------------------
BAC02725_Periplaneta_americana     --------------------------------------------------
ADM34986.1_1_B_tabaci_B            --------------------------------------------------
CAJ19121_Blattella_germanica       --------------------------------------------------
H2CLV8_Antheraea_pernyi            --------------------------------------------------
XP_008180459_Acyrthosiphon_pisum   --------------------------------------------------
UPI000252AABD_Apis_florea          --------------------------------------------------
UPI0001758603_Tribolium_castaneum  --------------------------------------------------
T1KD20_Tetranychus_urticae         AKCNCQNGYNGSRCEYNICNCKNGATCLASGGEGKCICSPGFTGKLCETV
UPI0002063B8E_Apis_mellifera       --------------------------------------------------
E2FLQ4_Bombyx_mori                 --------------------------------------------------

Bemisia_tabaci_Asia_1              -----------------------------THFLETTGTVDG---------
T1E9W2_Anopheles_aquasalis         -----------------------------SWTVR----------------
P98163_Drosophila_melanogaster     -----------------------------A--VNSLELEHE---------
B4Q2Q9_Drosophila_yakuba           -----------------------------A--VNSLELEQE---------
B4IGA9_Drosophila_sechellia        -----------------------------A--VNSLELEQE---------
K4MUW4_Actias_selene               -----------------------------GWTYQDYQRGHR---------
Q1T728_Rhyparobia_maderae          -----------------------------GIDTNTEVMRKK---------
O76952_Aedes_aegypti               -----------------------------THLGEAAPNVHD---------
Q6X0I2_Solenopsis_invicta          ------------------------------KFVESINFSRN---------
ADE34166.1_Nilaparvata_lugens      -----------------------------VTYLTSNSPVQT---------
D5KXW4_Nilaparvata_lugens          -----------------------------VTYLTSNSPVQT---------
BAC02725_Periplaneta_americana     ------------------------------WFEKVTPRGKQ---------
ADM34986.1_1_B_tabaci_B            -----------------------------THFLETTGTVDG---------
CAJ19121_Blattella_germanica       -----------------------------SHGLDSSKESGG---------
H2CLV8_Antheraea_pernyi            -----------------------------GWTYQDYQRGHR---------
XP_008180459_Acyrthosiphon_pisum   -----------------------------DLDYKKN--------------
UPI000252AABD_Apis_florea          -----------------------------SLVTYKNESIRH---------
UPI0001758603_Tribolium_castaneum  -----------------------------GRRVKALHYVNN---------
T1KD20_Tetranychus_urticae         TDPKCDNFICLNGGYCMVKDDLPYCECPAGWKGSKCEKVKTGDEICDHYC
UPI0002063B8E_Apis_mellifera       -----------------------------SLVIYKNESIRH---------
E2FLQ4_Bombyx_mori                 -----------------------------GWTYQDYQRGHR---------

Bemisia_tabaci_Asia_1              --------------------------------------------------
T1E9W2_Anopheles_aquasalis         --------------------------------------------------
P98163_Drosophila_melanogaster     --------------------------------------------------
B4Q2Q9_Drosophila_yakuba           --------------------------------------------------
B4IGA9_Drosophila_sechellia        --------------------------------------------------
K4MUW4_Actias_selene               --------------------------------------------------
Q1T728_Rhyparobia_maderae          --------------------------------------------------
O76952_Aedes_aegypti               --------------------------------------------------
Q6X0I2_Solenopsis_invicta          --------------------------------------------------
ADE34166.1_Nilaparvata_lugens      --------------------------------------------------
D5KXW4_Nilaparvata_lugens          --------------------------------------------------
BAC02725_Periplaneta_americana     --------------------------------------------------
ADM34986.1_1_B_tabaci_B            --------------------------------------------------
CAJ19121_Blattella_germanica       --------------------------------------------------
H2CLV8_Antheraea_pernyi            --------------------------------------------------
XP_008180459_Acyrthosiphon_pisum   --------------------------------------------------
UPI000252AABD_Apis_florea          --------------------------------------------------
UPI0001758603_Tribolium_castaneum  --------------------------------------------------
T1KD20_Tetranychus_urticae         LNSGNCTFTGGHFPPSCRCPAGWIGPRCQIAEGCKGFCLNGGLCKLTSDP
UPI0002063B8E_Apis_mellifera       --------------------------------------------------
E2FLQ4_Bombyx_mori                 --------------------------------------------------
```

Transmembrane region

```
Bemisia_tabaci_Asia_1              -----------------------QSPGYSWSSICATIILVAFIGTTFYAL
T1E9W2_Anopheles_aquasalis         --------------------------------------------------
```

```
P98163_Drosophila_melanogaster       ---------------------HEQNGHFHWLMALFVLAAGSLIAGLGYMY
B4Q2Q9_Drosophila_yakuba             -----------------------QHGHFHWLMALFVLAAGSLIAGLGYMY
B4IGA9_Drosophila_sechellia          ---------------------QEQNGHFHWLMALLVLAAGSLIAGLGYMY
K4MUW4_Actias_selene                 --------------------------ASITVVIAVLVLFLVYIALFVYYH
Q1T728_Rhyparobia_maderae            -------------------QSSEFNAKGFVVGLVILILIAGAVLGAYYYK
O76952_Aedes_aegypti                 -------------------SSDESSTGSTIANVFLVLLILALVGVAGGYI
Q6X0I2_Solenopsis_invicta            ----------------------TRNISGIYSITIIVLLVSVLLLCVYYYY
ADE34166.1_Nilaparvata_lugens        ----------------------SSLGSAFWMFLAFIFVVVPVVGILWFVY
D5KXW4_Nilaparvata_lugens            ----------------------SSLGSAFWMFLAFIFVVVPVVGILWFVY
BAC02725_Periplaneta_americana       -------------GGKSEEMQHSSNVGGIIIAILVIALVVGGVAAVYYYK
ADM34986.1_1_B_tabaci_B              -----------------------QSPGYSWSSICATIILVAFIGTTFYAL
CAJ19121_Blattella_germanica         ------------------TQAAGKSSAGLVMGLIIIAVIIATLLGAYYYK
H2CLV8_Antheraea_pernyi              --------------------------ASITVVIAVLVLFLVYIALFVYYH
XP_008180459_Acyrthosiphon_pisum     ----------------------------MISIYISIFLIVMLLLAMLYYF
UPI000252AABD_Apis_florea            -------------------------QNGTLIGIIITVLICIIIGSAYFYY
UPI0001758603_Tribolium_castaneum    ---------------------YASSNLGPVIAGVMISICLVTFGAVLYYL
T1KD20_Tetranychus_urticae           LKTPYCICPPLSEGKRCEKARSSSTAGPVAMTVITICVVVVTLVFLIMFG
UPI0002063B8E_Apis_mellifera         -------------------------QNGTLIGIIITVLACIIIGSAYFYY
E2FLQ4_Bombyx_mori                   --------------------------ASITVVIAVLVLFLVYIALFVYYH
```

Cytoplasmic region

```
Bemisia_tabaci_Asia_1                FYYYNS-----------KYNMRRLF--PSIHFKNP---AFNLQSKFQANG
T1E9W2_Anopheles_aquasalis           --------------------------------------------------
P98163_Drosophila_melanogaster       YQYRQ------------RGHTDLNI---NMHFQNPLATLGGTKAFLEHER
B4Q2Q9_Drosophila_yakuba             YQYRK------------RGHTDLNI---NMHFQNPLATLSGTKGFMEHER
B4IGA9_Drosophila_sechellia          YQYRQ------------RGHTDLNI---NMHFQNPLATLGGTKAFLDHER
K4MUW4_Actias_selene                 FVYKP------------KRKRSTAY--TEVRFQNS---------------
Q1T728_Rhyparobia_maderae            YGF--------------NKDSKFGF---SMHFSNP---TFGVQS------
O76952_Aedes_aegypti                 YRR--------------RYQHKFDI---GMHFHNP---------------
Q6X0I2_Solenopsis_invicta            QKNKL------------KSKPASNLSCSSIHFQNP---SYDRSDEI----
ADE34166.1_Nilaparvata_lugens        RGGPPSVPLSPPQWMPGFCARRFPF--HTIRFNSK----FGNLDADDTIP
D5KXW4_Nilaparvata_lugens            RGGPPSVPLSPPQWMPGFCARRFPF--HTIRFNSK----FGNLDADDTIP
BAC02725_Periplaneta_americana       RFG--------------YKGPKLNF---SLHFKNP---TFGIKE------
ADM34986.1_1_B_tabaci_B              FYYYNS-----------KYNMRRLF--PSIHFKNP---AFNLQSKFQANG
CAJ19121_Blattella_germanica         YRG--------------KKNSKFGF---SMHFQNP---TFGVRN------
H2CLV8_Antheraea_pernyi              FVYKP------------KRKRSTAY--TEVRFQNS---------------
XP_008180459_Acyrthosiphon_pisum     LFYSK------------NKSLNQLV---SRLFHRPLTT------------
UPI000252AABD_Apis_florea            QKIKP------------NFSKKNNL---SIHFQNP---SYDQRN------
UPI0001758603_Tribolium_castaneum    IKR--------------RNSGTFNV--ISMKFHNP---GYGLH-------
T1KD20_Tetranychus_urticae           LRIRARS--------RAFMHQRIDESAANLEISNP---MFGDDVEDDCYT
UPI0002063B8E_Apis_mellifera         QKIKP------------NFSKKNNL---SIHFQNP---SYDQRN------
E2FLQ4_Bombyx_mori                   FVYKP------------KRKRSTAY--TEVRFQNS---------------
```

```
Bemisia_tabaci_Asia_1                M-----------------------------------TGLASGNHMAHLSS
T1E9W2_Anopheles_aquasalis           --------------------------------------------------
P98163_Drosophila_melanogaster       AEAGVGFTTETGTVSSRGSNDT-FTTTSATSSFAAQQFSVPNALQRLLRP
B4Q2Q9_Drosophila_yakuba             AEAGVGFAPETGTVSSRGSNDTFTTTTTTSSSFVAQQFSVPNALQRLLRP
B4IGA9_Drosophila_sechellia          TEAGVGFATETGTVSSRGSNDT-FTTTSASSSFAAQQFSVPNALQRLLRP
K4MUW4_Actias_selene                 ---------------------------------------SDEAAQLSCSP
Q1T728_Rhyparobia_maderae            ------------------------------------PESHLDTPSHSLVP
O76952_Aedes_aegypti                 -----------------------------------ELSTADAAEVKMFQK
Q6X0I2_Solenopsis_invicta            ------------------------------------EVMLDSMASSELSP
ADE34166.1_Nilaparvata_lugens        ------------------------------------AYSDFQFHPCQLNP
D5KXW4_Nilaparvata_lugens            ------------------------------------AYSDFQFHPCQLNP
BAC02725_Periplaneta_americana       ---------------------------------------SDVAVPQVLVP
ADM34986.1_1_B_tabaci_B              M-----------------------------------TGLASGNHMAHLSS
CAJ19121_Blattella_germanica         ------------------------------------STENPSTPPQGLVP
H2CLV8_Antheraea_pernyi              ---------------------------------------SDEAAQLSCSP
XP_008180459_Acyrthosiphon_pisum     ------------------------------------VIQVIDQKEDEINS
UPI000252AABD_Apis_florea            ------------------------------------EIAPTLNCISGLPP
UPI0001758603_Tribolium_castaneum    ---------------------------------------TDEFDRSILKP
T1KD20_Tetranychus_urticae           ------------------------------------TEHPTQTFSLKSED
UPI0002063B8E_Apis_mellifera         ------------------------------------EIAPTLNCISGLPP
E2FLQ4_Bombyx_mori                   ---------------------------------------SDEAAQLSCSP
```

```
Bemisia_tabaci_Asia_1                KDHHFENPLQESRE-----GEVRIVTPNEITISR-AETSWTSAHLEDSSS
T1E9W2_Anopheles_aquasalis           --------------------------------------------------
P98163_Drosophila_melanogaster       RQSASGDPMAQELLLESPSRESKLHALDGGGAGG-DGDGGRGVGRQVPDI
B4Q2Q9_Drosophila_yakuba             RQAASGDPMAQELLLESP-RESNLHSVDGGAGG-----CGGGVGRQVPDI
```

```
B4IGA9_Drosophila_sechellia          RQSASGDPMAQELLLESP-RESKLHSLDGGGDGC-------GVGRQVPDI
K4MUW4_Actias_selene                 AVQMNGNQLINGNEFVNPLQYVRNVWQQSIRRKP-RPVCTAGLSIAVPNS
Q1T728_Rhyparobia_maderae            GQHQYVNPFDSAGVREHPDGKVVMIPHEKISPAK-LETPTHTAEADEGAE
O76952_Aedes_aegypti                 VPRLNQTTTHNELTLETPPHRPPCQGDPNSSEST-GQNNVTSTALELENM
Q6X0I2_Solenopsis_invicta            GQHEYINPINNKGMKAAE-----------NNAKK-SNQCSEGKNIEEEKQ
ADE34166.1_Nilaparvata_lugens        GEHQYENPIAAMQAEQNSGAAITIKTMNEIDIQM-GEEKNGWMAGGGEGG
D5KXW4_Nilaparvata_lugens            GEHQYENPIAAMQAEQNSGAAITIKTMNEIDIQM-GEEKNGWMAGGGEGG
BAC02725_Periplaneta_americana       GQHQYTNPFDNAEALKQL--EGSVIQESRLKKLA-DH-------IQLEDE
ADM34986.1_1_B_tabaci_B              KDHHFENPLQESRE-----GEVRIVTPNEITISR-AETSWTSAHLEDSSS
CAJ19121_Blattella_germanica         GQHQYTNPFDSSGVLEHP--DGKVLILSQQEKPP-VIIRIPSQSSERTDA
H2CLV8_Antheraea_pernyi              AVQMNGNQLINGNEFVNPLQYVRNVWQQSIRRKP-RPVCTAGLSIAVPNS
XP_008180459_Acyrthosiphon_pisum     RYNVYENPMNEDGLIKDHACGSEIVLNYDSARYV-NA-----NCSEPPLN
UPI000252AABD_Apis_florea            GEHEYVNPII------------------DIQKNQ-NENITQKNEKQMTKI
UPI0001758603_Tribolium_castaneum    GQHEYENPVEFYGSENDNHTNIQVNV------------------------
T1KD20_Tetranychus_urticae           KSKNFSNPMYEFQQTIDEEKRNLLVEKNDMTEFPKDELNGESNGVIGDGL
UPI0002063B8E_Apis_mellifera         GEHEYVNPII------------------DIQKNQ-NENITQKNEKQMTKI
E2FLQ4_Bombyx_mori                   AVQMNGNQLINGNEFVNPLQYVRNVWQQSIRRKS-RPVCTAGLSIAVPNS


Bemisia_tabaci_Asia_1                IETEYADLVVE-----------TNPKANLIS-
T1E9W2_Anopheles_aquasalis           --------------------------------
P98163_Drosophila_melanogaster       LVADMDDDAAKSAGQFGGNYAGNDANARFVS-
B4Q2Q9_Drosophila_yakuba             LVADMDDDAAKSGGQFGGHYAGDDANARFVP-
B4IGA9_Drosophila_sechellia          LVADMDDDAAKSAGQFGGNYAGDDANARFVS-
K4MUW4_Actias_selene                 PQQDFSDTESDLDDRET---KRFILKNKFLN-
Q1T728_Rhyparobia_maderae            MEDDTDQGFIT---------DTDSMKVKLIP-
O76952_Aedes_aegypti                 SDVDSMEDAYD---------CRDDPLQRLIL-
Q6X0I2_Solenopsis_invicta            DALIYFVHNSK---------------------
ADE34166.1_Nilaparvata_lugens        DDSDSSTVEMP--------------EVKLLNL
D5KXW4_Nilaparvata_lugens            DDSDSSTVEMP--------------EVKLLNL
BAC02725_Periplaneta_americana       DAEDYAPDGSD--------------KAPLIH-
ADM34986.1_1_B_tabaci_B              IETEYADLVVE-----------TNPKANLIS-
CAJ19121_Blattella_germanica         ADFEMEDDTSQ--EFVS---DNNDMKAPLIS-
H2CLV8_Antheraea_pernyi              PQQDFSDTESDLDDRET---KRFILKNKFLN-
XP_008180459_Acyrthosiphon_pisum     SFSEYDENEKK--------------KLIMVL-
UPI000252AABD_Apis_florea            LNFDRSDNESE------E---SAYKQDISLI--
UPI0001758603_Tribolium_castaneum    --------------------------------
T1KD20_Tetranychus_urticae           PAFLLSELVVPGHFKTTKEILDTKLSDPLYVG
UPI0002063B8E_Apis_mellifera         LNFDRSDNESE------E---SANKQDISLI--
E2FLQ4_Bombyx_mori                   PQQDFSDTESDLDDRET---KRFIRKNKFLN-
```
